# Impact of agro-industrial by-products on nutritional value and microbiota composition of black soldier fly larvae

**DOI:** 10.3389/fmicb.2026.1766582

**Published:** 2026-02-19

**Authors:** Martina Kieβling, Thorben Sieksmeyer, Christian Hertel, Andreas Juadjur, Kemal Aganovic, Volker Heinz, Kashif ur Rehman

**Affiliations:** 1 German Institute of Food Technologies (DIL e. V.), Quakenbrück, Germany; 2 Institute of Food Quality and Food Safety, University of Veterinary Medicine Hannover, Hannover, Germany; 3 Department of Microbiology, Faculty of Veterinary and Animal Sciences, The Islamia University of Bahawalpur, Bahawalpur, Pakistan

**Keywords:** 16S rRNA gene sequencing, agro-industrial by-product, *Hermetia illucens*, lactic acid bacteria, microbiota

## Abstract

In this study, interrelations between agro-industrial by-products, black soldier fly larvae (BSFL) rearing, and associated microbiota were investigated. Carrot pomace, brewer’s yeast, spent grains, and rape press cake were used as feed substrates, varying in their chemical composition, nutritional value, and microbial load and diversity. Overall, the data did not reveal a consistent or direct relationship between substrate chemical composition and nutrient profile of the BSFL, suggesting that larval development may be influenced more by complex substrate-microbe-larva interactions than by substrate chemistry alone. Using brewer’s yeast, the highest average larval biomass (184.3 mg/larva) and crude protein content (61.1%), as well as high crude fat content (24.3%), were obtained. Fatty acid analyses of BSFL revealed diverse patterns with high saturated (stearic acid, palmitic acid, myristic acid, lauric acid) or unsaturated (oleic acid, linoleic acid) fatty acid contents in BSFL reared on carrot pomace and brewer’s yeast, or on rape press cake, respectively. The composition of the substrate, either nutrient (dietary fibre) or microbiota-wise, markedly influenced the BSFL microbiota. Several species of lactic acid bacteria and *bacilli* were found to be potentially transferred from the substrate to the BSFL microbiota. On the other hand, several taxa of the genera *Actinomyces*, *Morganella*, *Klebsiella*, and *Enterococcus* were identified to belong to the core microbiota of BSFL, independent of the substrate. The study advances our understanding of how substrate selection affects the performance, nutrition, and microbiota of BSFL, providing insight into the possibilities for sustainable waste management and protein production systems.

## 1 Introduction

*Hermetia illucens* (black soldier fly, BSF) is a species of fly that belongs to the family Stratiomyidae in the order Diptera (Sheppard et al., 1994; Ji-bin et al., 2020). This fly was formerly found only in the tropical and warm temperate zones of America but now expanded its range to include many other parts of the planet (Tomberlin and Sheppard, 2002; Diener et al., 2009). The larvae of BSF have been hailed as a potentially useful organism for the management of by-products and organic wastes on account of their remarkable capacity to transform discarded food into usable biomass (Purkayastha and Sarkar, 2023; Rehman et al., 2023; Soomro et al., 2024). Meanwhile, in Europe, the market of BSFL is predicted to reach a value of $2.29 billion and a volume of 4.6 million tonnes by 2033, with compound annual growth rates of 31.3 and 40.6%, respectively (Meticulous Research, 2023). This increase in demand is mostly due to the increased interest in using BSFL in aquaculture, poultry production, and the pet food industry (Smetana et al., 2019). The interest arises from BSFL’s high nutritional content and the ability to partially replace standard feed additives as fish meal or soy products (Rehman et al., 2023).

Industrial by-products are a diverse range of leftover materials produced by various agricultural and industrial activities (Bibi et al., 2023). These by-products are often the result of operations like agricultural harvesting, food processing, and biofuel manufacturing (Gupta et al., 2023), and have considerable economic, environmental, and societal potential. The key issue is to identify effective and sustainable methods for dealing with these by-products (Eixenberger et al., 2022; Colombo et al., 2023), because inadequate management can result in pollution, waste of resources, and missed chances for value creation (Eixenberger et al., 2022). It is critical to develop novel strategies for collecting, processing, and upcycling industrial by-products to maximise their potential while minimising the related management issues. One approach to recycling by-products that are permitted in Europe for the rearing of edible insects is to use them for the rearing of BSFL to produce protein, fat, and macromolecules. These nutrients could be used to feed livestock, pets, and aquaculture (Somroo et al., 2019; Rehman et al., 2022, 2023). BSFL can transform many different types of organic waste, i.e., crop residues, foods, vegetables, soybean curd residues, and pineapple crowns (Rehman et al., 2017a, 2023; Wang et al., 2017; Shao et al., 2024), and animal manure from dairy, chicken and pig (Ji-bin et al., 2020; Rehman et al., 2017b, 2019), into useful insect larval biomass. Compared to more conventional methods of waste management like composting or landfilling, this process offers several advantages, e.g., nutrient recycling, resource utilisation, value-added products, and reduction of environmental impact due to lowering landfill waste and carbon dioxide emissions (Rehman et al., 2017a, 2023; Liu et al., 2020).

As intensive rearing practices grow more common, the requirement for strong quality criteria linked to microbiological safety (feed safety) and the manufacturing of standardised products becomes more apparent (Ma et al., 2018; Soomro et al., 2021; She et al., 2023). These requirements are critical for the continuous growth of insect farming in Europe. Understanding the characteristics and interactions between the microbiota of substrates and BSFL is essential for assuring the safety of BSFL during future processing (Xiang et al., 2022). Furthermore, this information may be used to improve the rearing conditions of BSFL. A potent technique for determining the diversity of microbiota on a genetic level is provided by metagenomics (Singh et al., 2015). Phylogenetic classification of members of the microbiota is often based on marker genes, such as the 16S rRNA gene. However, only a few studies have looked at the microbiota of grown insects in terms of substrate choice (Gold et al., 2022).

In this study, the impact of agro-industrial by-products used as feed substrates on the weight gain and chemical composition of BSFL, as well as the effect of the substrate’s microbiota on the larval microbiota, was investigated. The by-products carrot pomace, brewer’s yeast, spent grains and rape press cake have been selected, as each is regionally available in Lower Saxony, Germany, and each has unique chemical compositions, thus providing different nutritional values for BSFL rearing. Therefore, the objective of this study was to systematically investigate the impact of chemically and microbiologically distinct agro-industrial by-products on (i) BSFL growth and biomass yield, (ii) larval nutritional and fatty acid composition, and (iii) gut microbial abundance, diversity, and composition. We hypothesized that (1) feed substrates with contrasting nutrient profiles would differentially affect larval growth and body composition, (2) the fatty acid composition of BSFL would partially reflect the fatty acid profile of the respective substrates, and (3) substrate-associated bacteria would contribute to the larval gut microbiota, while a core set of bacterial taxa would be consistently present across larvae irrespective of diet. By addressing these hypotheses, this study aims to improve the understanding of diet microbiota-host interactions in BSFL and support the optimised and safe use of agro-industrial by-products in insect-based bioconversion systems.

## 2 Materials and **methods**

### 2.1 Colony maintenance

The larvae of *Hermetia illucens* L. (black soldier fly, BSFL) used in this study were bred at the German Institute of Food Technologies (DIL; Quakenbrück, Germany). The breeding system was installed in a greenhouse at 27 °C with 60–70% relative humidity and 14 h:10 h (light: dark) time ratio. BSF were kept in cages of 1.8 m^3^ with a 1.5 mm mesh size. Commercial laying hen meal (GS Agri, Schneiderkrug, Germany) with addition of 5% (w/w) sunflower oil was used as a feed substrate for the first 6-day growth phase of larvae (Khan et al., 2025).

### 2.2 Feed substrates and their chemical analysis

The following agro-industrial by-products were used as feed substrates: Carrot pomace (AHN GmbH, Warmsen, Germany), brewer’s yeast as well as spent grains (Leiber, Bramsche, Germany), and rape press cake (Brökelmann + Co – Oelmühle GmbH + Co, Hamm, Germany). Commercial laying hen meal (GS Agri, Schneiderkrug, Germany) supplemented with 5% (w/w) sunflower oil was used as control feed. All substrates had no legal limitations to be used as substrates for animal feed in farms, either as a single component or in blends (EC, 2001). Each substrate’s nutritional composition, moisture content, and contents of starch, monosaccharides, disaccharides, and ash were determined according to German standard methods for feed described in §64 LFGB, L00.00-19/3 2004-07 (Bundesamt für Verbraucherschutz und Lebensmittelsicherheit, 2004). All analyses were performed in duplicates. The amount of digestible carbohydrates was calculated as the sum of the contents of starch, monosaccharides, and disaccharides (Dossey et al., 2016). All substrates were packed, kept in vacuum-sealed plastic bags, and stored at 2 °C for a maximum of 4 months. Before use, single substrates were ground to a particle size <1 mm using a lab mill with a 1 mm sieve (GM 200, Retsch, Haan, Germany) (Kieβling et al., 2023).

### 2.3 Experimental design

Six days after hatching, approximately 2,000 larvae of an average weight of 3.3 ± 0.4 mg/larva were placed into each rearing box (32 cm × 25 cm × 10 cm). Each rearing box represented one replicate; thus, approximately 2,000 larvae were used per replicate. The experiment consisted of five groups in total: one control feed group and four experimental groups corresponding to the four substrates. Each group was set up in three replicates in individual rearing boxes. Water was added to the dry substrates to obtain a wet substrate containing 67% moisture. In every box, 2.3 kg (wet weight) of freshly prepared substrate was filled in. The amount of wet substrate was given in surplus and was not exchanged during the rearing period. The BSFL were kept under the same conditions as described for colony maintenance. The feeding period was 11 days. For microbiological analysis, approximately 15 g of larvae were collected manually from each rearing box and placed in sterile stomacher bags. The adhering substrate was removed from the larval surface through manual stripping with a tweezer. Then the bags were sealed and stored at 8 °C till analysis. The remaining larvae per rearing box were used for weight determination and chemical analysis; larvae were frozen at −20 °C (Ma et al., 2018; Kieβling et al., 2023).

### 2.4 Determination of nutritional values

For nutritional values of BSFL, the nutritional composition, moisture content, and contents of starch, monosaccharides, and disaccharides were determined according to the German standard methods for feed described in §64 LFGB, L00.00-19/3 2004-07 (Bundesamt für Verbraucherschutz und Lebensmittelsicherheit, 2004). Each analysis was carried out in duplicate. The total amounts of starch, monosaccharides, and disaccharides were summed up to calculate the amount of total digestible carbohydrates. For the determination of fatty acids of the larvae, proteins and carbohydrates were separated from the lipid fraction. For preparation of fatty acid methyl esters, samples were heated directly with a methanolic sulfuric acid solution after freeze-drying in accordance with ISO 12966-2. In the second processing step (ISO 12966-4), the fatty acid methyl esters were separated by capillary gas chromatography on a highly polar stationary phase depending on their chain length, the degree of saturation, and the geometry and positions of the double bonds [gas chromatography with flame ionisation detector (GC-FID)]. The equipment used consisted of a gas chromatograph GC-2010 plus FID (Shimadzu Europa mbH, Duisburg, Germany) with the separation column Agilent HP 88, 100 m, film thickness 0.20 m, and ID 0.25 mm.

### 2.5 Preparation of first dilutions

The collected larval samples (three replicates per treatment; see 2.3) were homogenised in their sample bags by crushing with a rolling pin. From the homogenised larvae or the previously collected substrate samples (one sample per substrate; see 2.3), 10 g were aseptically transferred to stomacher bags (INTERSCIENCE) and diluted with 90 mL of sterile maximum recovery diluent (Oxoid). Samples were homogenised in a stomacher (BagMixer 400; INTERSCIENCE) for 2 min (first dilution). From these first dilutions, 5 mL were frozen and stored at −20 °C until needed for 16S rRNA gene sequencing. The remaining first dilutions were immediately used to determine the microbial counts.

### 2.6 Microbial counting

Decimal dilutions were prepared, and aliquots of 0.1 mL of the appropriate dilutions were spread and incubated in duplicates using the following parameters: Plate count agar (PCA, Oxoid) incubated at 30 °C for 3 days to count mesophilic aerobic bacteria; Yeast extract glucose chloramphenicol agar (YGC; Oxoid) incubated at 25 °C for 4 days to count yeasts and molds; Violet red bile dextrose agar (VRBD; Oxoid) incubated anaerobically at 30 °C for 2 days in an anaerobic jar (AnaeroGen, Oxoid) to count Enterobacteriaceae. After appropriate incubation, the colony-forming units (CFU) were counted.

### 2.7 Full-length 16S rRNA gene sequencing

DNA was isolated using the DNeasy PowerLyzer PowerSoil kit (Qiagen) according to the manufacturer with some modifications. Briefly, 1.8 mL homogenate were pelleted by centrifugation at 10,000 *g* for 2 min. The pellet was resuspended in 750 μL of PowerBead solution and transferred to a PowerBead tube. Sixty μl of solution C1 were added, and the sample was homogenised in a Bead Ruptor 24 Elite (OMNI International) at 6 m/s for 4 min. For insect samples, an additional Proteinase K treatment was introduced after bead milling. Hundred μg Proteinase K (A&A BIOTECHNOLOGY) were added to the homogenate and incubated overnight at 56 °C and 600 rpm (Rubin et al., 2014). For both feed and insect samples, the DNA isolations were completed using the entire PowerSoil protocol with a final elution in 50 μL of solution C6. DNA quality was examined with a NanoDrop™ One microvolume UV-Vis spectrophotometer (Thermo Scientific), and the concentration was measured with a Qubit 4 fluorometer (Invitrogen) using the 1× dsDNA HS kit (Invitrogen) according to the manufacturer.

PCRs of isolated DNA samples were performed using custom-made barcoded primer pairs in unique combinations targeting the full-length 16S rRNA gene (Supplementary Table 1). The exact PCR composition was as follows: 0.5 μL of 10 μM primer each, 25 μL of 2× KAPA HiFi HotStart ReadyMix (Roche), 25 ng of template DNA and filled up to a final volume of 50 μL with PCR-grade water. In the case of carrot pomace and spent grains, 24 μL of the template DNA were used due to low DNA concentration. PCR cycling parameters were: 1 min at 95 °C, and 35 cycles with 95 °C for 20 s, 55 °C for 30 s and 72 °C for 2 min with a final extension at 72 °C for 5 min. The PCR products were purified with an Ampure XP bead clean-up (BECKMAN COULTER Life Sciences) according to the manufacturer using 30 μL beads. The purified PCR products were then used for the library preparation for the 16 s rRNA gene sequencing according to the Ligation sequencing amplicons protocol (SQK-LSK109; Oxford Nanopore), and sequencing was performed on a MinION sequencing platform according to the manufacturer (Oxford Nanopore).

### 2.8 Data analysis

SigmaPlot statistical software (Systat Software, Windows version 13.0, SigmaPlot, San José, CA, USA) was used to investigate the statistical significance of changes in mean larval live weight, ANOVA (*p* < 0.05) followed by Tukey HSD tests. To evaluate variability, nutritional and fatty acid compositions of the samples were statistically analysed by calculating means and standard deviation with Microsoft Excel. For microbial counts, mean values and standard deviations were calculated and logarithmised (log10) in Microsoft Excel. After sequencing, bases were called in super high accuracy mode using Guppy v6.4.8 (Oxford Nanopore), and demultiplexing was done using Minibar v0.25 (Krehenwinkel et al., 2019) with a barcode edit distance value of 11 and barcode trimming. Taxonomic identification was done using Emu v3.4.4 (Curry et al., 2022) with the prebuilt database (version from 18th August 2022) expanded by plant species likely occurring in the substrates (Supplementary file: List S1). The 16S sequences of the plants were taken from PhytoRef and added manually to the existing database (Decelle et al., 2015). Further data analysis was performed in R v4.2.2 (R Core Team, 2022), where first plant hits and unassigned hits were filtered out. The highest portion of plant and unassigned sequences was filtered out for the rape press cake feed substrate, with mean values at 21.5 and 38.2%, respectively. For the other feed substrates, portions of plant and unassigned sequences being filtered out ranged from 0 to 20.7% and from 1.2 to 34.4%. The larval data set contained no plant sequences, and portions of unassigned sequences ranging from 0.8 to 12.7% were filtered out. Further Shannon indices, the PCoA map based on Bray–Curtis dissimilarity approach and the heatmap were calculated using phyloseq v1.42.0 (McMurdie and Holmes, 2013). Additionally, graphs were produced with ggplot2 v3.4.1 (Villanueva and Chen, 2019).

## 3 Results

### 3.1 Chemical composition of feed substrates

Chemical composition of the agro-industrial by-products carrot pomace, brewer’s yeast, spent grains, and rape press cake used as feed substrates, as well as of the control feed, are listed in Table 1. Substantial differences across all chemical parameters were detected. Based on dry matter, crude protein, crude lipid, digestible carbohydrate, and crude fibre content of the feed substrates varied widely between 7.9 to 47.7%, 2.3 to 19.0%, 4.1 to 48.8%, and 0.5 to 28.4%, respectively. Crude protein was found to be highest in brewer’s yeast (47.7%), whereas crude lipid was highest in rape press cake (19.0%). Remarkably, carrot pomace showed the lowest contents of crude protein (7.9%) and crude lipid (2.3%), however, the highest content in crude fibres (28.4%). Regarding the fatty acid composition, the concentration of saturated, monounsaturated, and polyunsaturated fatty acids varied from 8.2 to 63.0%, 5.5 to 58.6%, and 8.2 to 66.5%, respectively (Table 1).

**Table 1:** Chemical composition of feed substrates used for BSFL rearing (mean; *n* = 2).

Parameter	Unit	Carrot pomace	Brewer’s yeast	Spent grain	Rapeseed press cake	Control feed
Dry matter (DM)	g/100 g	92.7	96.1	93.5	94.9	88.3
Ash	g/100 g DM	5.0	6.7	7.3	6.5	6.7
Crude protein	g/100 g DM	7.9	47.7	26	28.9	17.7
Crude lipid	g/100 g DM	2.3	4.3	11.0	19.0	10.1
Crude fibre	g/100 g DM	28.4	<0.5	17.1	18.0	4.5
Digestible carbohydrate	g/100 g DM	34.3	11.1	4.1	11.4	48.8
Saturated fatty acids	g/100 g lipid	27.9	63	29.8	8.2	17.5
Monounsaturated fatty acids	g/100 g lipid	5.5	23.7	11.6	58.6	24.7
Polyunsaturated fatty acids	g/100 g lipid	66.5	13.3	58.6	8.2	57.7

### 3.2 Nutritional value of BSFL grown on agro-industrial by-products

BSFL grew on all feed substrates, resulting in average individual larval biomasses of 210.5 mg, 184.3 mg, 150.2 mg, 42.2 mg, and 36.5 mg for the control feed, brewer’s yeast, rape press cake, spent grains and carrot pomace, respectively (Supplementary Table 2). As shown in Table 2, the chemical compositions of BSFL biomasses were strongly impaired by the type of feed substrate. Differences were highest in the crude lipid contents (approximately up to 6-fold), ranging from 5.8% for carrot pomace up to 30.3% for rape press cake. For the crude protein content, smaller differences were found (approximately up to 1.4-fold), being highest in the BSFL fed with brewer’s yeast (61.1%). A certain difference was also observed for the ash content, being lowest in the BSFL fed with the brewer’s yeast (5.6%) and highest using the carrot pomace as substrate (14.7%). The pH values of the BSFL biomass were 7.9, 6.6, 7.8, 6.9, and 7.1 when grown on the carrot pomace, brewer’s yeast, spent grains, rape press cake, and control feed, respectively. The data did not reveal a consistent or direct relationship between substrate chemical composition and nutrient profile of the BSFL (Table 2).

**Table 2:** Chemical composition of BSFL grown on various agro-industrial by-products for 11 days (mean ± standard deviation; *n* = 3).

Parameter	Composition of BSFL grown on				
	Carrot pomace	Brewer’s yeast	Spent grain	Rapeseed press cake	Control feed
Crude protein (g/100 g DM)	49.79 ± 1.94	61.10 ± 1.30	44.00 ± 3.11	50.20 ± 0.46	46.53 ± 2.83
Crude lipid (g/100 g DM)	5.79 ± 0.25	24.3 ± 1.48	8.15 ± 0.21	30.30 ± 0.82	23.23 ± 1.96
Ash (g/100 g DM)	14.74 ± 0.32	5.65 ± 0.17	12.20 ± 0.85	7.76 ± 0.23	11.03 ± 0.06
Dry matter (DM, %)	18.00 ± 0.75	33.70 ± 1.10	25.20 ± 1.65	32.53 ± 1.01	29.83 ± 1.26
pH	7.94 ± 0.01	6.59 ± 0.02	7.78 ± 0.06	6.89 ± 0.02	7.06 ± 0.09
Fatty acids (saturated) (g/100 g DM)	3.46 ± 0.30	18.70 ± 1.00	4.47 ± 0.17	9.53 ± 2.08	15.23 ± 1.81
Fatty acids (monounsaturated) (g/100 g DM)	1.24 ± 0.03	4.63 ± 0.59	1.52 ± 0.02	13.70 ± 1.22	3.54 ± 0.28
Fatty acids (polyunsaturated) (g/100 g DM)	1.12 ± 0.05	0.94 ± 0.14	2.17 ± 0.05	7.03 ± 0.69	4.43 ± 0.33

Determination of the compositions of saturated, monounsaturated, and polyunsaturated fatty acids of BSFL biomasses (Tables 2 and 3) showed an impact of the feed substrate (Table 1). Saturated fatty acid (SFA) contents were highest in the BSFL grown on brewer’s yeast (18.70%) and low when BSFL were fed with carrot pomace (3.46%) and spent grains (4.47%). In contrast, monounsaturated fatty acid (MUFA) contents were high in BSFL grown on rape press cake (13.70%), compared to the use of carrot pomace (1.24%) or spent grains as feed substrate. Similarly, polyunsaturated fatty acid (PUFA) contents were found to be highest in BSFL grown on rape press cake (7.03%) and lowest when grown on the brewer’s yeast. As shown in Table 3, lauric acid (C12:0) was by far the most abundant SFA in BSFL, ranging in content from 52.16% when grown in brewer’s yeast compared to rape press cake (18.37%), followed by palmitic acid (C16:0), ranging from 7.07 to 17.03%. Regarding the MUFAs, oleic acid (C18:1) was found in high concentrations, ranging from 11.80 to 40.04%, when the BSFL were grown on brewer’s yeast and rape press cake, respectively. Linoleic acid (C-18:2) was most abundant among the PUFAs and present in high concentrations in BSFL grown on carrot pomace, brewer’s yeast and rape press cake (Tables 2, 3). Moreover, the concentration of alpha-linolenic acid (18:3, *ω*-3) in BSFL also depended on the feed substrate and was found to be highest in BSFL grown on rape press cake (6.53%).

**Table 3:** Fatty acid composition of BSFL grown on agro-industrial by-products for 11 days (mean ± standard deviations).

Fatty acid	Concentration of fatty acid (%) in the BSFL grown on				
	Carrot pomace	Brewer’s yeast	Spent grain	Rapeseed press cake	Control feed
Caprylic acid (C-8:0)	0.28 ± 0.01	<0.1	0.17 ± 0.01	<0.1	<0.1
Capric acid (C-10:0)	1.38 ± 0.10	1.64 ± 0.11	1.07 ± 0.05	0.50 ± 0.11	0.99 ± 0.13
Lauric acid (C-12:0)	27.83 ± 3.51	52.16 ± 2.52	26.40 ± 0.16	18.37 ± 5.29	39.2 ± 2.57
Myristic acid (C-14:0)	5.43 ± 0.26	8.31 ± 0.19	4.82 ± 0.07	3.35 ± 0.79	8.39 ± 0.41
Myristoleic acid (C-14:1)	1.68 ± 0.15	0.55 ± 0.04	0.97 ± 0.09	0.27 ± 0.03	0.21 ± 0.03
Pentadecanoic acid (C-15:0)	1.30 ± 0.12	<0.1	0.49 ± 0.01	0.15 ± 0.03	0.12 ± 0.01
Palmitic acid (C-16-0)	17.03 ± 0.70	11.63 ± 0.59	16.97 ± 0.21	7.07 ± 0.51	13.03 ± 0.21
Palmitoleic acid (C-16:1)	5.28 ± 0.73	6.68 ± 0.55	4.13 ± 0.18	4.89 ± 0.06	2.19 ± 0.15
Heptadecanoic acid (C-17)	1.41 ± 0.16	<0.1	0.41 ± 0.01	0.15 ± 0.01	0.21 ± 0.01
Stearic acid (C-18-0)	3.68 ± 0.20	2.80 ± 0.20	3.33 ± 0.05	1.34 ± 0.09	3.08 ± 0.06
Oleic acid (C-18:1)	14.43 ± 0.38	11.80 ± 1.04	13.23 ± 0.32	40.04 ± 4.21	12.76 ± 0.47
Linoleic acid (C-18:2)	17.40 ± 1.83	3.37 ± 0.28	24.07 ± 0.74	16.77 ± 1.50	18.60 ± 2.43
Linolenic acid, gamma (C-18:3)	0.41 ± 0.12	<0.1	0.78 ± 0.07	<0.1	<0.1
Linolenic acid, alpha (C-18:3)	1.18 ± 0.10	0.36 ± 0.02	1.93 ± 0.05	6.53 ± 0.86	0.51 ± 0.07
Nonadecanoic acid (C-19:0)	0.14 ± 0.02	<0.1	<0.1	<0.1	<0.1
Arachidic acid (C-20:0)	0.46 ± 0.08	0.167 ± 0.01	0.35 ± 0.02	0.19 ± 0.05	0.28 ± 0.01
Behenic acid (C-22:0)	0.41 ± 0.07	0.14 ± 0.01	0.38 ± 0.02	0.14 ± 0.02	0.22 ± 0.03
Docosapentaenoic acid (C-22:5)	<0.1	0.13 ± 0.02	<0.1	<0.1	<0.1
Lignoceric acid (C-24:0)	0.11 ± 0.00	<0.1	0.13 ± 0.02	<0.1	<0.1

### 3.3 Microbial counts of feed substrates and BSFL

Microbial counts of the feed substrates were found to be generally low (<approximately 5.0 log_10_ CFU/g), except for the count of mesophilic aerobic bacteria for spent grains (7.33 log_10_ CFU/g, Table 4). In all feed substrates, counts of Enterobacteriaceae and yeasts were below the limit of quantification, except for the control feed (4.48 and 2.00 log_10_ CFU/g, respectively). In contrast, after rearing, BSFL showed much higher microbial counts than the respective feed substrates (Table 4). Mesophilic aerobic bacterial counts ranged from 8.39 to 9.33 log_10_ CFU/g, and counts of Enterobacteriaceae were found to be higher than 6.0 log_10_ CFU/g. With carrot pomace, the BSFL biomass showed high counts of yeast and molds (>7.0 log_10_ CFU/g). Again, no correlation was found between the microbial loads of the substrates and those of the larvae, independent of the microbial groups.

**Table 4:** Microbial load of feed substrates (one sample per substrate measured in duplicates) used for rearing of BSFL and obtained BSFL biomass (3 technical replicates per substrate, each measured in duplicates; mean ± standard deviations).

Feed substrate	Microbial counts (mean ± standard deviation, log_10_ CFU/g)							
	Mesophilic aerobic bacteria		Enterobacteriaceae		Yeasts		Molds	
	Substrate	BSFL	Substrate	BSFL	Substrate	BSFL	Substrate	BSFL
Carrot pomace	2.85 ± 2.63	9.04 ± 8.62	<1.0^++^	>6.0^*^	<2.0^++^	>7.0^*^	<2.0^++^	>7.0^*^
Brewer’s yeast	3.27 ± 1.85	8.39 ± 7.95	<1.0^++^	>6.0^*^	<2.0^++^	5.24 ± 4.67	<2.0^++^	4.19 ± 3.76
Spent grain	7.33 ± 5.85	9.33 ± 9.01	<1.0^++^	>6.0^*^	<2.0^++^	5.60 ± 5.49	2.70 ± 2.15	5.15 ± 4.94
Rape press cake	3.51 ± 2.85	9.15 ± 8.69	<1.0^++^	>6.0^*^	<2.0^++^	5.79 ± 5.65	<2.0^++^	5.27 ± 4.78
Control feed	5.27 ± 4.33	9.05 ± 8.43	4.48 ± 4.05	>6.0^*^	2.00^+^	6.93 ± 6.17	3.35 ± 3.13	5.19 ± 4.77

^*^, Both duplicates above upper quantification limit; ^++^, both duplicates below lower quantification limit; ^+^, one duplicate below lower quantification limit.

### 3.4 Bacterial diversity of feed substrates and BSFL

**Figure 1:**
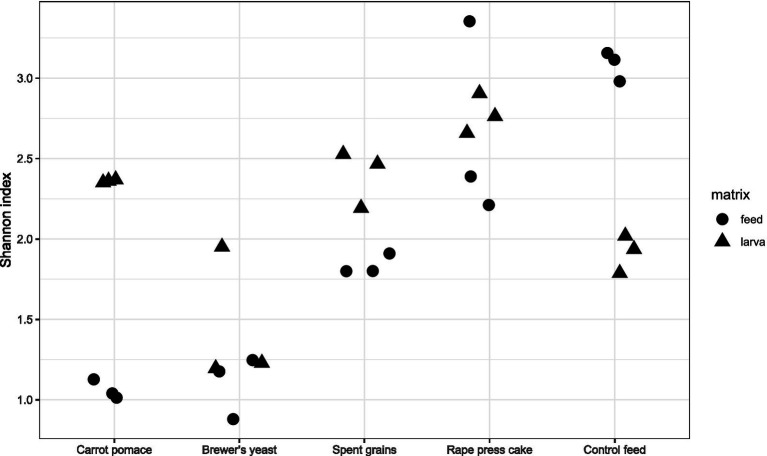
Shannon index used to estimate the combined bacterial and archaeal alpha diversity of feed substrates (dots) and their corresponding black soldier fly larvae (triangles) at bacteria genus level.

**Figure 2:**
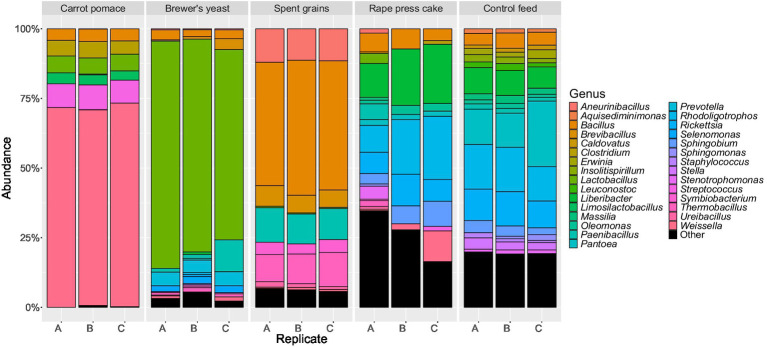
Relative abundance of the overall most prevalent bacteria and archaea genera in the feed substrates for black soldier fly larvae rearing.

**Figure 3:**
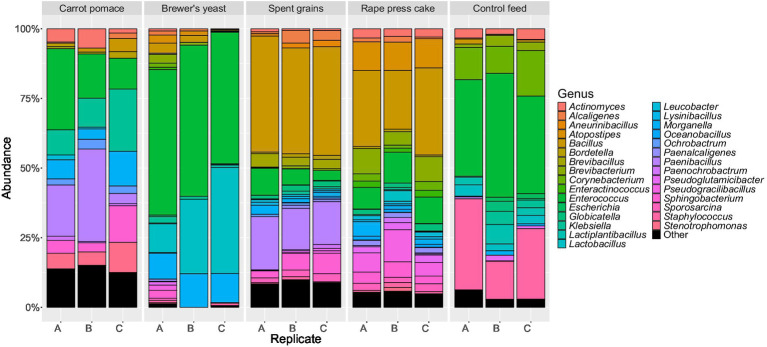
Relative abundance of the overall most prevalent bacteria and archaea genera in the black soldier fly larvae raised on different feed substrates.

Alpha diversity at genus level was measured using the Shannon diversity index (Figure 1). BSFL reared on brewer’s yeast, spent grains and rape press cake had diversity levels in ascending order which are similar to the levels of the corresponding feed substrates (Figure 1). In contrast, BSFL reared on carrot pomace and control feed had intermediate diversity levels, while the feed substrate carrot pomace showed relatively low diversity, comparable to brewer’s yeast, and the control feed even higher diversity like the outlier of rape press cake. The findings were also reflected by the bacterial abundances at the genus level. The control and rape press cake feed harboured numerous different genera (>20) in comparable abundances (Figure 2), while the corresponding BSFL mainly contained *Enterococcus* (30–45%), *Staphylococcus* (14–29%), and *Corynebacterium* (10–16%) and *Bacillus* (21–31%), *Pseudogracilibacillus* (5–12%), *Atopostipes* (10%), and *Brevibacterium* (5–9%), respectively (Figure 3). In contrast, the carrot pomace feed (Figure 2) is mostly dominated by *Weissella* (70–73%,), while the corresponding BSFL (Figure 3) harboured a more diverse microbiota consisting among others of *Paenibacillus* (4–33%), *Enterococcus* (11–29%), *Klebsiella* (9–22%), and *Sphingobacterium* (3–13%). The other feed substrates were mainly dominated by one up to a couple bacterial genera, namely *Lactobacillus* (68–82%) in brewer’s yeast or *Bacillus* (44–49%), *Paenibacillus* (11%), *Aneurinibacillus* (11%), *Thermobacillus* (10–12%), and *Brevibacillus* (6%) in spent grains (Figure 2). The first three of these genera were also found in high relative abundances in the corresponding larvae (Figure 3), suggesting the presence of similar taxa in both feed substrates and larvae, which may reflect potential transfer and/or selective enrichment. In brewer’s yeast-fed larvae, *Lactobacillus* accounted for 10–38%, while in spent grains-fed larvae, *Bacillus* (38–42%) and *Paenibacillus* (15–19%) were dominant. Furthermore, the genus *Enterococcus* was consistently detected at high relative abundances in larvae regardless of the feed substrate (11–54%).

**Figure 4:**
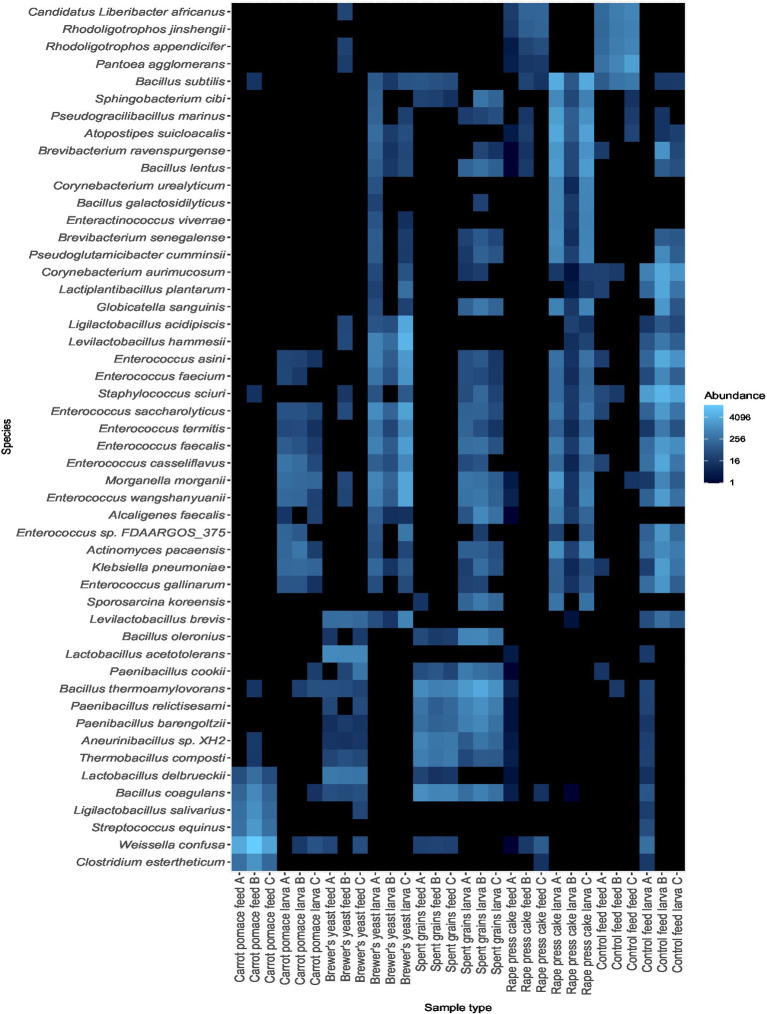
The heatmap shows the relative abundance of the 50 most abundant bacterial species of the feed substrates and their corresponding larvae.

Further evidence for shared bacterial taxa between feed substrates and corresponding larvae in the case of brewer’s yeast and spent grains is provided by the heat map (Figure 4), which shows the relative abundance of the 50 most abundant species. For brewer’s yeast, *Levilactobacillus brevis* was detected in both the feed substrate and larvae, suggesting potential transfer or selective enrichment. This pattern was more pronounced for spent grains, where numerous *Bacillus*, *Paenibacillus*, *Thermobacillus*, *Aneurinibacillus*, and *Brevibacillus* species were present in both the feed substrate and corresponding larvae. Notably, around 50% of those bacterial species are also found in the brewer’s yeast feed substrate, though in lower abundances, but not in the corresponding larvae (Figure 4). To a lesser extent, *Bacillus subtilis* might also be transferred from the feed substrate, rape press cake and control feed to the corresponding larvae. Contrarily, it was also found in spent grains but not in the corresponding larvae and in brewer’s yeast-fed larvae but not in the substrate. Besides these findings, it becomes apparent that the microbiota of different reared larvae is much more similar to each other than to the corresponding feed substrates (Figure 4). This is supported by the presence of *Actinomyces pacaensis*, *Klebsiella pneumoniae*, *Morganella morganii*, and several *Enterococcus* species in the microbiota of all larvae (Figure 4). This bacterial microbiota conformity of the different larvae treatments is further supported by their beta diversity, which was measured using the Bray-Curtis method at the genus level (Supplementary Figure 1). Besides the feed substrate, spent grains and the corresponding larvae, all other feed substrates and likewise all other larvae were grouped, indicating that their microbiota are more similar to each other than to the corresponding larvae or feed substrate (Supplementary Figure 1).

## 4 Discussion

The study’s findings permit insights into how agro-industrial by-products can affect the nutritional and microbiological composition of BSFL when used as feed substrates. The differences in chemical and microbiota composition among feed substrates and their subsequent impact on the composition of BSFL highlight the complex connection between the type of feed and larval development, nutritional profile, and microbiota. An unexpected finding was the non-appearance of a distinct link between the chemical content of the feed substrates (Table 1) and the corresponding BSFL (Table 2). For example, brewer’s yeast, having the highest protein content, revealed BSFL with the highest protein content but also with high lipid content, although the substrate contained less lipids (Table 1). Our observations may be explained by various causes, one of which is the larvae’s metabolic adaptability (Liu et al., 2017; Seyedalmoosavi et al., 2022). This adaptability enables them to regulate the absorption and utilisation of nutrients based on the specific elements present in the substrate (Seyedalmoosavi et al., 2022). In addition, the existence of non-nutritional elements, such as anti-nutritional compounds, in the substances could hinder the absorption of nutrients (Soni et al., 2022). This adds to the complexity of the connection between the feed and the composition of larvae. For instance, the substantial amount of dietary fibre included in carrot pomace may have hindered the larvae’s capacity to effectively use the nutrients that were accessible, leading to reduced growth and buildup of nutrients. It was previously reported that fibres in the BSFL substrate reduce the BSFL larvae development and have effects on the larvae gut health (Tschirner and Simon, 2015; Gold et al., 2018; Ji-bin et al., 2020; Rehman et al., 2023). Our data indicates that as well, since larvae reared on carrot pomace also have the lowest biomass.

The data indicate a significant association between the fatty acid compositions of the substrates (Table 1) and those of the BSFL that were raised on the substrates (Tables 2 and 3). For example, using substrates that contain a greater amount of saturated fatty acids, like brewer’s yeast (63 g/100 g), led to the production of larvae with higher levels of saturated fatty acids (18.7 g/100 g). In contrast, substrates that contain high amounts of polyunsaturated fatty acids (PUFAs), such as carrot pomace (66.49 g/100 g), resulted in larvae with reduced PUFA levels (1.12 g/100 g). These results are not consistent with those of other studies showing that larval fatty acids are significantly manipulated with substrates and that there is a positive correlation between the fatty acid concentration in the substrate and the concentration in BSF (Riekkinen et al., 2022). However, the present study suggests that the larvae selectively assimilate or modify fatty acids during their metabolic processes. The data indicates that although there is a certain degree of correlation, the larvae do not exactly replicate the fatty acid composition of the substrate. The BSFL is known to possess a highly active *de novo* lipogenesis pathway, enabling the synthesis of medium-chain saturated fatty acids, particularly lauric acid (C12:0), from non-lipid carbon sources such as carbohydrates and amino acids (Zhu et al., 2019). Protein-rich and readily digestible substrates, such as brewer’s yeast, may enhance the availability of acetyl-CoA and reduce equivalents, thereby stimulating fatty acid synthase activity and favouring the biosynthesis of lauric acid (Olzhausen et al., 2021). In contrast, lipid-rich substrates like rape press cake predominantly supply long-chain fatty acids (e.g., oleic and linoleic acids), which may be preferentially incorporated into larval lipids and concurrently downregulate endogenous fatty acid synthesis mechanisms (Mahmoud et al., 2026). This metabolic regulation explains the higher lauric acid accumulation observed in larvae fed a low-fat, high-protein substrate and is consistent with previous studies reporting limited capacity to directly tailor BSFL lauric acid content through dietary lipid manipulation alone (Ewald et al., 2020; Li et al., 2022).

The gut microbiome of BSFL has been described as comprising a relatively stable set of predominant bacterial taxa that persist across different feeding regimes and are thought to contribute to general metabolic functions involved in substrate degradation (Klammsteiner et al., 2020). In the present study, taxa belonging to the genera *Actinomyces*, *Morganella*, *Klebsiella*, and *Enterococcus* (Figures 3, 4) were consistently detected in all BSFL samples, irrespective of the feed substrate, and were therefore considered part of a putative core microbiota defined by their ubiquitous presence in all larvae samples. This observation is in line with previous studies reporting these genera as recurrent members of the BSFL gut microbiome (Klammsteiner et al., 2020; Yang et al., 2021; IJdema et al., 2022). It should be noted that some of these genera include opportunistic or potentially pathogenic species like *M. morganii* and *K. pneumonia,* which can cause diseases in livestock and humans (Falagas et al., 2006; Zhao et al., 2012; Effah et al., 2020), and were also detected in our study (Figure 4). However, it could also be shown that they might even be beneficial for the larvae (IJdema et al., 2022; Ravoitytė et al., 2025). Additionally, several common post-harvest processing methods like pasteurisation or simply freezing, drying and/or heating are normally applied, which contribute to the inactivation of such potential pathogens (Ravoitytė et al., 2025) and thus, ensuring food and/or feed safety demanded by the corresponding authorities. Several studies have demonstrated that such processing methods effectively lower microbial loads in insect biomass to levels compliant with food and feed safety standards (Larouche et al., 2019; Alagappan et al., 2025; Ravoitytė et al., 2025). Moreover, the strength of the core microbiota of the BSFL against extrinsic disturbances was supported by our Shannon index alpha diversity data. Despite large variances in the diversity of the substrate microbiota, the diversity of the BSFL microbiota remained in a comparable range, except for brewer’s yeast, where both substrate and BSFL microbiota exhibited reduced diversity (Figure 1). This exception indicates that external factors like nutrients can shape the microbiome. The reduced diversity might be explained by the substrates’ fibre content, as brewer’s yeast had the lowest fibre concentration (Table 1). Dietary fibres have a major impact on the diversity and richness of the gut microbiome, as demonstrated repeatedly in human studies (Cronin et al., 2021), and in a recent study on BSFL (Reyer et al., 2025). They provide numerous substrates for fermentation reactions carried out by various species of microorganisms (Cronin et al., 2021). The impact of fibre is further supported by the data of the control feed, having the second lowest fibre content (Table 1), resulting in the second lowest diversity of the BSFL microbiota (Figure 1).

Moreover, our data indicates that the composition of the feed substrate microbiota might be an even stronger factor than the substrates’ nutritional composition for shaping the BSFL’s microbiome. Certain bacteria like lactic acid bacteria species, e. g. *Levilactobacillus brevis* in the case of brewer’s yeast, different *bacilli* species belonging to the genera *Bacillus*, *Brevibacillus* and *Paenibacillus* in the case of spent grains, *Bacillus subtilis* in the case of rape press cake, or *Weissella confusa* in the case of carrot pomace seem to be transferable between feed substrate and BSFL (Figure 4). The selective nature of such a transfer, where only certain but not all bacteria of the feed substrate are transmitted to the BSFL microbiome, could be mediated by larvae’s microbiome through nutrient competition, niche occupation or immune priming (Engel and Moran, 2013). In the latter case, commensal bacteria could induce immune priming events resulting in constant activation or alteration of the immune system, not only towards recurrent colonisation of commensal bacteria but also against potential invaders (Engel and Moran, 2013). In addition, the immune system may also further contribute to the selectivity of the transfer by direct recognition of potential invaders. The protective nature of the commensal microbiome and the immune system are most likely also the reason in our study, why the microbial load of the BSFL was not drastically influenced by the feeding substrate (Table 4), neither through differing nutrient profiles (Table 1) nor differing microbial loads (Table 4). Those protective measures can also counteract against the transfer of pathogens from the feed substrate to the BSFL. For instance, Gorrens et al. (2021) could show in inoculation trials with *Staphylococcus aureus* added to the feed substrate that the larvae reduced counts below the detection limit (2.0 log CFU/g) and significantly decreased by at least 3.0 log CFU/g in the substrate. Such reducing effects in inoculated substrates were also shown for *Salmonella* sp. (Erickson et al., 2004; Lalander et al., 2015; Lopes et al., 2020; Grisendi et al., 2022). However, low levels (2.0 log CFU/g) of *Salmonella* sp. were detectable in the larvae at the end of the experiment (Erickson et al., 2004; Grisendi et al., 2022). These examples further underline the selective nature of the transfer of bacteria from the substrate to the BSFL, and together with our findings, they raise the question of why, in some cases, the larvae’s biological barriers are great hurdles for the transfer of microbes, while in some other cases, they are not. It further needs to be investigated whether the transferred bacteria in our study, and likewise the *Salmonella* sp. from other studies, are only transient or if they can permanently establish themselves as members of the BSFL microbiome, since no “starvation” or “purging” step prior to sampling to empty the gut contents was undergone. Therefore, it could be that the bacteria detected may simply be transient passengers from substrate residue remaining in the gut rather than true colonisers.

## 5 Conclusion

The findings of this research indicate that there is a substantial amount of difference in the chemical composition of agro-industrial by-products that were utilised as feed substrates and BSFL. Brewer’s yeast was found to be an extremely nutrient-dense feed that had the greatest crude protein content, which resulted in a considerable increase in the amount of protein present in the BSFL. On the other hand, carrot pomace brought about the highest levels of crude fibre and carbohydrates, despite the fact that it included a relatively low amount of protein and lipids. Furthermore, the study reveals the varied fatty acid profiles of BSFL, which exhibit significant differences in saturated, monounsaturated, and polyunsaturated fatty acids depending on the feed substrate. It is important to note that larvae that were fed brewer’s yeast had the highest concentration of lauric acid, whereas larvae that were fed rape press cake had the highest concentrations of oleic and linoleic acids. It becomes apparent that while all larvae shared a core microbiome, the composition of the feed substrate, either nutrient or microbiota-wise markedly shaped the BSFL microbiome. The dietary fibre content, on the one hand, significantly influenced the diversity of the larval microbiome, and on the other hand, particular bacterial species were selectively transferred from the substrates to the larvae. Such transfer of microorganisms from substrates to BSFL might be used as a tool to introduce beneficial microorganisms to the BSFL to promote insect growth, health or nutritional profile. However, the mechanisms that cause the selectivity of transfer and whether transferred taxa remain only temporarily or establish themselves permanently as members of the BSFL microbiome still need to be investigated in more detail.

## Data Availability

The datasets presented in this study can be found in online repositories. The names of the repository/repositories and accession number(s) can be found in the article/Supplementary material.
