# Supplementary material for: Impact of agro-industrial by-products on nutritional value and microbiota composition of black soldier fly larvae
Source: Front Microbiol. 2026 Feb 19;17:1766582. doi: 10.3389/fmicb.2026.1766582 (PMC12960147; doi:10.3389/fmicb.2026.1766582)
Supplement: Supplementary file 1 [file Data_Sheet_1.pdf]

Supplementary Material

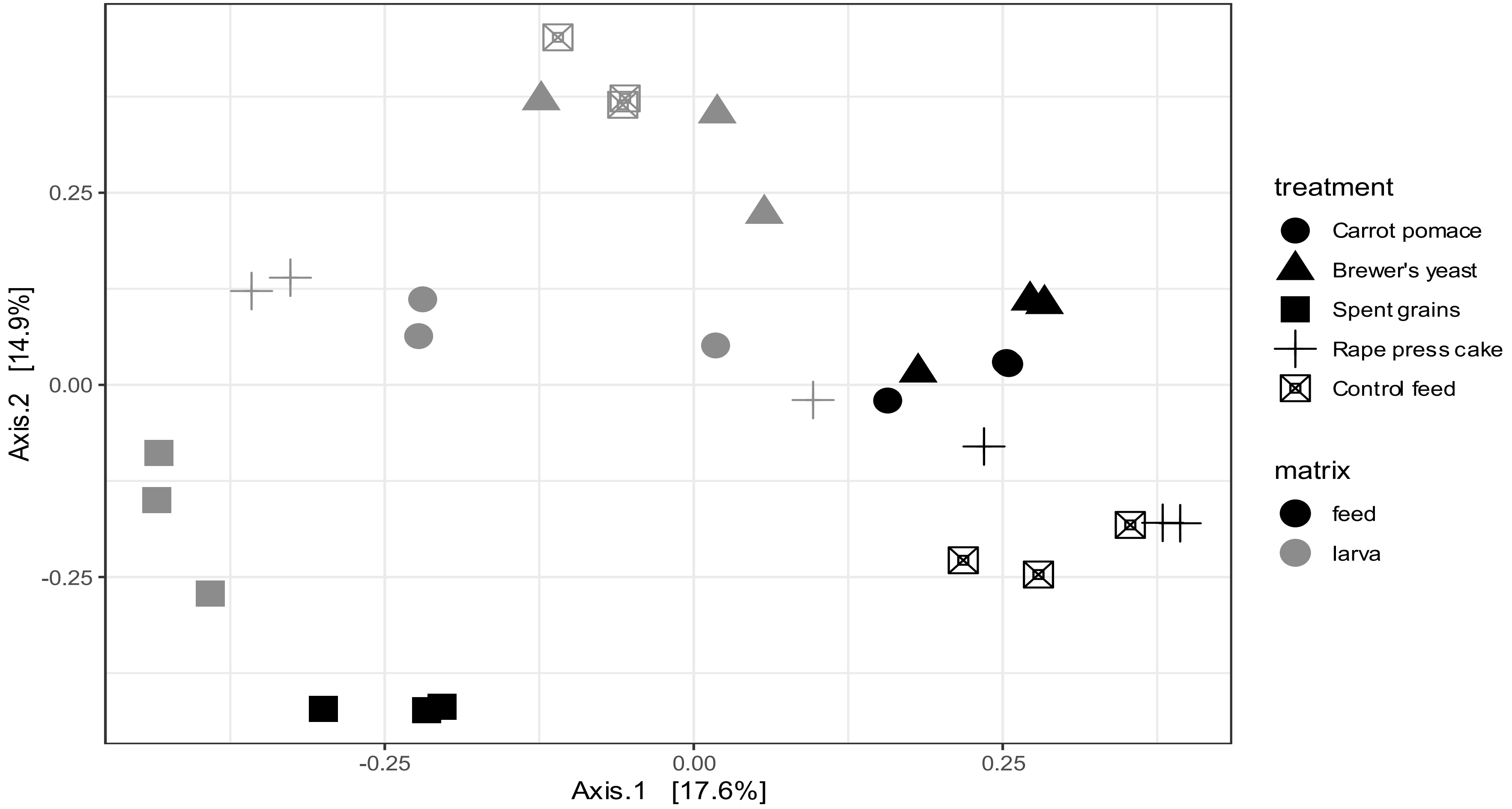

Supplementary Figure 1. PCoA map based on Bray-Curtis dissimilarity approach (beta diversity) comparing the feed substrates (black) and their corresponding black soldier fly larvae (grey) at bacteria genus level.

Supplementary Table 1. Barcoded primer sequences targeting the full-length 16S rRNA gene (27F and 1492R). Forward primers are indicated by _F and reverse primers by _R at the end of the name tag. Barcode sequences are marked in grey.

Primer	5´-Sequence-3´
NB01_F	AAGGTTAACACAAAGACACCGACAACTTTCTTCAGCACCTAGAGTTTGATCMTGGCTCAG
NB02_F	AAGGTTAAACAGACGACTACAAACGGAATCGACAGCACCTAGAGTTTGATCMTGGCTCAG
NB03_F	AAGGTTAACCTGGTAACTGGGACACAAGACTCCAGCACCTAGAGTTTGATCMTGGCTCAG
NB04_F	AAGGTTAATAGGGAAACACGATAGAATCCGAACAGCACCTAGAGTTTGATCMTGGCTCAG
NB05_F	AAGGTTAAAAGGTTACACAAACCCTGGACAAGCAGCACCTAGAGTTTGATCMTGGCTCAG
NB06_F	AAGGTTAAGACTACTTTCTGCCTTTGCGAGAACAGCACCTAGAGTTTGATCMTGGCTCAG
NB07_F	AAGGTTAAAAGGATTCATTCCCACGGTAACACCAGCACCTAGAGTTTGATCMTGGCTCAG
NB08_F	AAGGTTAAACGTAACTTGGTTTGTTCCCTGAACAGCACCTAGAGTTTGATCMTGGCTCAG
NB01_R	GGTGCTGTTCGGATTCTATCGTGTTTCCCTATTAACCTTAGCAATCGGTTACCTTGTTACGACTT
NB02_R	GGTGCTGCTTGTCCAGGGTTTGTGTAACCTTTTAACCTTAGCAATCGGTTACCTTGTTACGACTT
NB03_R	GGTGCTGTTCTCGCAAAGGCAGAAAGTAGTCTTAACCTTAGCAATCGGTTACCTTGTTACGACTT
NB04_R	GGTGCTGGTGTTACCGTGGGAATGAATCCTTTTAACCTTAGCAATCGGTTACCTTGTTACGACTT

Supplementary Table 2. Black soldier fly larvae biomass development on various agro-industrial by-products (mean ± standard error; n=3). Mean values followed by the same letter in the same column do not vary significantly ($P < 0.05$).

**Parameters**	**Black soldier fly larvae**				
	Carrot pomace	Brewer's yeast	Spent grains	Rape press cake	Control feed
Larval weight (mg)	$36.5 \pm 4.3^{d,e}$	$184.3 \pm 10.5^{a,b}$	42.2 ± 2.3^{d}	$150.2 \pm 16.4^{b,c}$	210.5 ± 20.1^{a}

List Supplementary Table 1. Plant 16S sequences taken from PhytoRef and added to the standard Emu database.Supplementary

>3888:emu_db:49244 ["3888:ncbi:49244 ['NC_014057.48227.49715 Pisum sativum 16S ribosomal RNA, chloroplast']"]

GCTCATGGAGAGTTTGATCCTGGCTCAGGATGAACGCTGGCGGCATGCTTTACAC
ATGCAAGTCGGACGGGAAGTGGTGTTTCCAGTGGCGAACGGGTGAGTAACGCGT
AAGAACCTGCCCTTGGGAGGGGGACAACAGCTGGAAACGGCTGCTAATACCCCG
TAGGCTGAGGAGCGAAAGGAGGAATCCGCCCAAGGAGGGGCTCGCGTCTGATTA
GCTAGTTGGTGAGGTAATAGCTTACCAAGGCGATGATCAGTAGCTGGTCCGAGAG
GATGATCAGCCACACTGGGACTGAGACAAGGTCCAGACTCCTACGGGAGGCAGC
AGTGGGGAATTTTCCGCAATGGGCGAAAGCCTGACGGAGCAATGCCGCGTGGAG
GTAGAGGCCCCTGGGTCATGAACTTCTTTTCCCGGAGAAGAAAAAATGACGGTAT
CCGGGGAATAAGCATCGGCTAACTCTGTGCCAGCAGCCGCGGTAAGACAGAGGA
TGCAAGCGTTATCCGGAATGATTGGGCGTAAAGCGTCTGTAGGTGGCTTTTTAAG
TTCGCTGTCAAATACCAGGGCTCAACCCTGGACAGGTGGTGAAAACTACTAAGCT
AGAGTACGGTAGGGGCAGAGGGAATTTCCGGTGGAGCGATGAAATGCGTAGAGA
TCGGAAGGAACACCAACGGCGAAAGCACTCTGCTGGGCCGACACTGACACTGAG
AGACGAAAGCTAGGGGAGCGAATGGGATTAGAGACCCCAGTAGTCCTAGCCGTA
AACGATGGATACTAAGTGGCTGTGCGTATCGACCCGTGCAACGCTGTAGCTAACG
CGTTAAGTATCCCGCCTGGGGAGTACGTTCGCAAGAATGAAACTCAAAGGAATTG
ACGGGGGCCCGCACAAGCGGTGGAGCATGTGGTTTAATTCGATGCAAAGCGAAG
AACCTTACCAGGGCTTGACATGCGCGAATCCTCTTGAAAGAGAGGAGTGCCTTCG
GGAATGCGACACAGGTGGTGCATGGCTGTCGTCAGCTCGTGCCGTGAGGTGTTGG
GTTAAGTCCCGCAACGAGCGCAACCCTCGTGTTTAGTTGCCAACGTTTAGTTTGG
AACTCTGAACAGACTGCCGGTGATAAGCCGGAGGAAGGTGAGGATGACGTCAAG
TCATCATGCCCCTTATGCCCTGGGCTACACACGTGCTACAATGGACCGGACAAAG
GATCGCGACCCCGCGAGGGTGAGCTAACTTCAAAAACCTGTCCTCAGTTCGGATT
GTAGGCTGCAACTCGCCTACATGAAGCCGGAATCGCTAGTAATCGCCGGTCAGCC
ATACGGCGGTGAATTCGTTCCCGGGCCTTGTACACACCGCCCGTCACACTATGGG
AGCTGGCCATGCCCGAAGTCATTACCTTAACCGCAAGGAGGGGGATGCCGAAGG
CAGGGCTAGTGACTGGAGTGAAGTCGTAACAAGGTAGCCGTACTGGAAGGTGCG
ACTGGATCACCTCCTTT

>3888:emu_db:49245 ["3888:ncbi:49245 ['HM029370.48227.49715_U Pisum sativum 16S ribosomal RNA, chloroplast']"]

GCTCATGGAGAGTTTGATCCTGGCTCAGGATGAACGCTGGCGGCATGCTTTACAC
ATGCAAGTCGGACGGGAAGTGGTGTTTCCAGTGGCGAACGGGTGAGTAACGCGT
AAGAACCTGCCCTTGGGAGGGGGACAACAGCTGGAAACGGCTGCTAATACCCCG
TAGGCTGAGGAGCGAAAGGAGGAATCCGCCCAAGGAGGGGCTCGCGTCTGATTA
GCTAGTTGGTGAGGTAATAGCTTACCAAGGCGATGATCAGTAGCTGGTCCGAGAG
GATGATCAGCCACACTGGGACTGAGACAAGGTCCAGACTCCTACGGGAGGCAGC
AGTGGGGAATTTTCCGCAATGGGCGAAAGCCTGACGGAGCAATGCCGCGTGGAG
GTAGAGGCCCCTGGGTCATGAACTTCTTTTCCCGGAGAAGAAAAAATGACGGTAT
CCGGGGAATAAGCATCGGCTAACTCTGTGCCAGCAGCCGCGGTAAGACAGAGGA
TGCAAGCGTTATCCGGAATGATTGGGCGTAAAGCGTCTGTAGGTGGCTTTTTAAG
TTCGCTGTCAAATACCAGGGCTCAACCCTGGACAGGTGGTGAAAACTACTAAGCT
AGAGTACGGTAGGGGCAGAGGGAATTTCCGGTGGAGCGATGAAATGCGTAGAGA
TCGGAAGGAACACCAACGGCGAAAGCACTCTGCTGGGCCGACACTGACACTGAG

AGACGAAAGCTAGGGGAGCGAATGGGATTAGAGACCCCAGTAGTCCTAGCCGTA
AACGATGGATACTAAGTGGCTGTGCGTATCGACCCGTGCAACGCTGTAGCTAACG
CGTTAAGTATCCCGCCTGGGGAGTACGTTCGCAAGAATGAAACTCAAAGGAATTG
ACGGGGGCCCGCACAAGCGGTGGAGCATGTGGTTTAATTCGATGCAAAGCGAAG
AACCTTACCAGGGCTTGACATGCGCGAATCCTCTTGAAAGAGAGGAGTGCCTTCG
GGAATGCGACACAGGTGGTGCATGGCTGTCGTCAGCTCGTGCCGTGAGGTGTTGG
GTTAAGTCCCGCAACGAGCGCAACCCTCGTGTTTAGTTGCCAACGTTTAGTTTGG
AACTCTGAACAGACTGCCGGTGATAAGCCGGAGGAAGGTGAGGATGACGTCAAG
TCATCATGCCCCTTATGCCCTGGGCTACACACGTGCTACAATGGACCGGACAAAG
GATCGCGACCCCGCGAGGGTGAGCTAACTTCAAAAACCTGTCCTCAGTTCGGATT
GTAGGCTGCAACTCGCCTACATGAAGCCGGAATCGCTAGTAATCGCCGGTCAGCC
ATACGGCGGTGAATTCGTTCCCGGGCCTTGTACACACCGCCCGTCACACTATGGG
AGCTGGCCATGCCCGAAGTCATTACCTTAACCGCAAGGAGGGGGATGCCGAAGG
CAGGGCTAGTGACTGGAGTGAAGTCGTAACAAGGTAGCCGTACTGGAAGGTGCG
ACTGGATCACCTCCTTT

>3888:emu_db:49246 ["3888:ncbi:49246 ['X51598.472.1961_U Pisum sativum 16S ribosomal RNA, chloroplast']"]

GAAGCTCATGGAGAGTTTGATCCTGGCTCAGGATGAACGCTGGCGGCATGCTTTA
CACATGCAAGTCGGACGGGAAGTGGTGTTTCCAGTGGCGAACGGGTGAGTAACG
CGTAAGAACCTGCCCTTGGGAGGGGGACAACAGCTGGAAACGGCTGCTAATACC
CCGTAGGCTGAGGAGCGAAAGGAGGAATCCGCCCAAGGAGGGGCTCGCGTCTGA
TTAGCTAGTTGGTGAGGTAATAGCTTACCAAGGCGATGATCAGTAGCTGGTCCGA
GAGGATGATCAGCCACACTGGGACTGAGACAAGGTCCAGACTCCTACGGGAGGC
AGCAGTGGGGAATTTTCCGCAATGGGCGAAAGCCTGACGGAGCAATGCCGCGTG
GAGGTAGAGGCCCCTGGGTCATGAACTTCTTTTCCCGGAGAAGAAAAAATGACG
GTATCCGGGGAATAAGCATCGGCTAACTCTGTGCCAGCAGCCGCGGTAAGACAG
AGGATGCAAGCGTTATCCGGAATGATTGGGCGTAAAGCGTCTGTAGGTGGCTTTT
TAAGTTCGCTGTCAAATACCAGGGCTCAACCCTGGACAGGTGGTGAAAACTACTA
AGCTAGAGTACGGTAGGGGCAGAGGGAATTTCCGGTGGAGCGATGAAATGCGTA
GAGATCGGAAGGAACACCAACGGCGAAAGCACTCTGCTGGGCCGACACTGACAC
TGAGAGACGAAAGCTAGGGGAGCGAATGGGATTAGAGACCCCAGTAGTCCTAGC
CGTAAACGATGGATACTAAGTGGCTGTGCGTATCGACCCGTGCAACGCTGTAGCT
AACGCGTTAAGTATCCCGCCTGGGGAGTACGTTCGCAAGAATGAAACTCAAAGG
AATTGACGGGGGCCCGCACAAGCGGTGGAGCATGTGGTTTAATTCGATGCAAAG
CGAAGAACCTTACCAGGGCTTGACATGCGCGAATCCTCTTGAAAGAGAGGAGTG
CCTTCGGGAATGCGACACAGGTGGTGCATGGCTGTCGTCAGCTCGTGCCGTGAGG
TGTTGGGTTAAGTCCCGCAACGAGCGCAACCCTCGTGTTTAGTTGCCAACGTTTA
GTTTGGAACTCTGAACAGACTGCCGGTGATAAGCCGGAGGAAGGTGAGGATGAC
GTCAAGTCATCATGCCCCTTATGCCCTGGGCTACACACGTGCTACAATGGACCGG
ACAAAGGATCGCGACCCCGCGAGGGTGAGCTAACTTCAAAAACCTGTCCTCAGTT
CGGATTGTAGGCTGCAACTCGCCTACATGAAGCCGGAATCGCTAGTAATCGCCGG
TCAGCCATACGGCGGTGAATTCGTTCCCGGGCCTTGTACACACCGCCCGTCACAC
TATGGGAGCTGGCCATGCCCGAAGTCATTACCTTAACCGCAAGGAGGGGGATGC
CGAAGGCAGGGCTAGTGACTGGAGTGAAGTCGTAACAAGGTAGCCGTACTGGAA
GGTGCGACTGGATCACCTCCT

>3888:emu_db:49247 ["3888:ncbi:49247 ['X55033.717.2227_U Pisum sativum 16S ribosomal RNA, chloroplast']"]

GAAGCTATAAGTAATGCAACTAGGAAGCTCATGGAGAGTTTGATCCTGGCTCAGG
ATGAACGCTGGCGGCATGCTTTACACATGCAAGTCGGACGGGAAGTGGTGTTTCC
AGTGGCGAACGGGTGAGTAACGCGTAAGAACCTGCCCTTGGGAGGGGGACAACA
GCTGGAAACGGCTGCTAATACCCCGTAGGCTGAGGAGCGAAAGGAGGAATCCGC
CCAAGGAGGGGCTCGCGTCTGATTAGCTAGTTGGTGAGGTAATAGCTTACCAAGG
CGATGATCAGTAGCTGGTCCGAGAGGATGATCAGCCACACTGGGACTGAGACAA
GGTCCAGACTCCTACGGGAGGCAGCAGTGGGGAATTTTCCGCAATGGGCGAAAG
CCTGACGGAGCAATGCCGCGTGGAGGTAGAGGCCCCTGGGTCATGAACTTCTTTT
CCCGGAGAAGAAAAAATGACGGTATCCGGGGAATAAGCATCGGCTAACTCTGTG
CCAGCAGCCGCGGTAAGACAGAGGATGCAAGCGTTATCCGGAATGATTGGGCGT
AAAGCGTCTGTAGGTGGCTTTTTAAGTTCGCTGTCAAATACCAGGGCTCAACCCT
GGACAGGTGGTGAAAACTACTAAGCTAGAGTACGGTAGGGGCAGAGGGAATTTC
CGGTGGAGCGATGAAATGCGTAGAGATCGGAAGGAACACCAACGGCGAAAGAA
CTCTGCTGGGCCGACACTGACACTGAGAGACGAAAGCTAGGGGAGCGAATGGGA
TTAGAGACCCCAGTAGTCCTAGCCGTAAACGATGGATACTAAGTGGCTGTGCGAT
ATCGACCCGTGCAACGCTGTACTAACGCGTTAAGTATCCCGCCTGGGGAGTACGT
TCGCAAGAATGAAACTCAAAGGAATTGACGGGGGCCGCACAAGCGGTGGAGCAT
GTGGTTTAATTCGATGCAAAGCGAAGAACCTTACCAGGGCTTGACATGCGCGAAT
CCTCTTGAAAGAGAGGAGTGCCTTCGGGAATGCGACACAGGTGGTGCATGGCTGT
CGTCAGCTCGTGCCGTGAGGTGTTGGGTTAAGTCCCGCAACGAGCGCAACCCTCG
TGTTTAGTTGCCAACGTTTAGTTTGGAACTCTGAACAGACTGCCGGTGATAAGCC
GGAGGAAGGTGAGGATGACGTCAAGTCATCATGCCCCTTATGCCCTGGGCTACAC
ACTGCTACAATGGACCGGACAAAGGATCGCGACCCCGCGAGGGTGAGCTAACTT
CAAAAACCTGTCCTCAGTTCGGATTGTAGGCTGCAACTCGCCTACATGAAGCCGG
AATCGCTAGTAATCGCCGGTCAGCCATACGGCGGTGAATTCGTTCCCGGGCCTTG
TACACACCGCCCGTCACACTATGGGAGCTGGCCATGCCCGAAGTCATTACCTTAA
CCGCAAGGAGGGGGATGCCGAAGGCAGGGCTAGTGACTGGAGTGAAGTCGTAAC
AAGGTAGCCGTACTGGAAGGTGCGACTGGATCACCTCCT

>3888:emu_db:49248 ["3888:ncbi:49248 ['M37430.751.2216_U Pisum sativum 16S ribosomal RNA, chloroplast']"]

AGAGTTTGATCCTGGCTCAGGATGAACGCTGGCGGCATGCTTTACACATGCAAGT
CGGACGGGAAGTGGTGTTTCCAGTGGCGAACGGGTGAGTAACGCGTAAGAACCT
GCCCTTGGGAGGGGGACAACAGCTGGAAACGGCTGCTAATACCCCGTAGGCTGA
GGAGCGAAAGGAGGAATCCGCCCAAGGAGGGGCTCGCGTCTGATTAGCTAGTTG
GTGAGGTAATAGCTTACCAAGGCGATGATCAGTAGCTGGTCCGAGAGGATGATC
AGCCACACTGGGACTGAGACAAGGTCCAGACTCCTACGGGAGGCAGCAGTGGGG
AATTTTCCGCAATGGGCGAAAGCCTGACGGAGCAATGCCGCGTGGAGGTAGAGG
CCCCTGGGTCATGAACTTCTTTTCCCGGAGAAGAAAAAATGACGGTATCCGGGGA
ATAAGCATCGGCTAACTCTGTGCCAGCAGCCGCGGTAAGACAGAGGATGCAAGC
GTTATCCGGAATGATTGGGCGTAAAGCGTCTGTAGGTGGCTTTTTAAGTTCGCTGT
CAAATACCAGGGCTCAACCCTGGACAGGTGGTGAAAACTACTAAGCTAGAGTAC
GGTAGGGGCAGAGGGAATTTCCGGTGGAGCGATGAAATGCGTAGAGATCGGAAG
GAACACCAACGGCGAAAGAACTCTGCTGGGCCGACACTGACACTGAGAGACGAA
AGCTAGGGGAGCGAATGGGATTAGAGACCCCAGTAGTCCTAGCCGTAAACGATG
GATACTAAGTGGCTGTGCGATATCGACCCGTGCAACGCTGTACTAACGCGTTAAG
TATCCCGCCTGGGGAGTACGTTCGCAAGAATGAAACTCAAAGGAATTGACGGGG
GCCGCACAAGCGGTGGAGCATGTGGTTTAATTCGATGCAAAGCGAAGAACCTTA
CCAGGGCTTGACATGCGCGAATCCTCTTGAAAGAGAGGAGTGCCTTCGGGAATGC
GACACAGGTGGTGCATGGCTGTCGTCAGCTCGTGCCGTGAGGTGTTGGGTTAAGT

CCCGCAACGAGCGCAACCCTCGTGTTTAGTTGCCAACGTTTAGTTTGGAACTCTG
AACAGACTGCCGGTGATAAGCCGGAGGAAGGTGAGGATGACGTCAAGTCATCAT
GCCCCTTATGCCCTGGGCTACACACTGCTACAATGGACCGGACAAAGGATCGCGA
CCCCGCGAGGGTGAGCTAACTTCAAAAACCTGTCCTCAGTTCGGATTGTAGGCTG
CAACTCGCCTACATGAAGCCGGAATCGCTAGTAATCGCCGGTCAGCCATACGGCG
GTGAATTCGTTCCCGGGCCTTGTACACACCGCCCGTCACACTATGGGAGCTGGCC
ATGCCCGAAGTCATTACCTTAACCGCAAGGAGGGGGATGCCGAAGGCAGGGCTA
GTGACTGGAGTGAAGTCGTAACAAGGTAGCCGTACTGGAAGGTGCGACTG

>3847:emu_db:49249 ["3847:ncbi:49249 ['X06428_Soybean_Streptophyta Glycine max 16S ribosomal RNA, chloroplast']"]

TCTCATGGAGAGTTCGATCCTGGCTCAGGATGAACGCTGGCGGCATGCCTTACAC
ATGCAAGTCGGACGGGAAGTGGTGTTTCCAGTGGCGGACGGGTGAGTAACGCGT
AAGAACCTACCCTTGGGAGGGGAACAACAGCTGGAAACGGCTGCTAATACCCCG
TAGGCTGAGGAGCAAAAGGAGAATCCGCCCGAGGAGGGGCTCGCGTCTGATTAG
CTAGTTGGTGAGGCATAGCTTACCAAGGCGATGATCAGTAGCTGGTCCGAGAGAT
GATCAGCCACACTGGGACTGAGACGAGGCCCAGACTCTTACGGGAGGCAGCAGT
GGGGAATTTTCCGCAATGGCGAAAGCTGACGGAGCAATGCCGCGTGAAGGTAGA
AGGCCTACGGTCATGAACTTCTTTTCCCGGAGAAGAAGCAATGACGGTGATCCGG
GGAATAAGCATCGGCTAACTCTGTGCCAGCGGCCGCGGTAAGACAGAGGATGCA
AGCGTTATCCGGAATGATTGGGCGAAAGGTCTGTAGGTGGCTTTTTAAGTTCGCC
GTCAAATCCCAGGGCTCAACCCTGGACAGGCGGTGGAAACTACCAAGCTGGAGT
ACGGTAGGGGCAGAGGGAATTTCCGGTGGAGCGGTGAAATGCGTAGAGATCGGA
AAGAACACCAACGGCGAAAGCACTCTGCTGGGCCGACACTGACACTGAGAGACG
AAACTTAGGGGAGCGAATGGGATTAGATACCCCAGTAGTCCTAGCCGTAAACGA
TGGATACTAGGCGCTGTGCGTATCGACCCGTGCAATGCTGTACTAACGCGTTAAG
TATCCCGCCTGGGGAGTACGTTCGCAAGAATGAAACTCAAAGGAATTGACGGGG
GCCGCACAAGCGGTGGAGCATGTGGTTTAATTCGATGCAAAGCGAAGAACCTTA
CCAGGGCTTGACATGCCGCGAATCCTCTTGAAAGAGAGGGGTGCTTCGGGAACG
CGGACACAGGTGGTGCATGGCTGTCGTCAGCTCGTGCCGTAAGGTGTTGGGTTAA
GTCCCGCAACGAGCGCAACCCTCGTGTTTAGTTGCCAACATTTAGTTTGGAACCC
TGAGCAGACTGCCGGTGATAAGCCGGAGGAAGGTGAGGATGACGTCGAAGTCAT
CATGCCCCTTATGCCCTGGGCGACACACGTGCTACAATGGACAAAGGATCGCGAT
CCCGCGAGGTGAGCTAACTCCAAAAACCCGTCCTCAGTTCGGATTGTAGGCTGCA
ACTCGCCTGCATGAAGCCGGAATCGCTAGTAATCGCCGGTCAGCCATACGGCGGT
GAATTCGTTCCCGGGCCTTGTACACACCGCCCGTCACACTATGGGAGCTGGCCAT
GCCGAAGTCGTTACCTTAACCGCAAGAGGGGGATGCCGAAGGGGCTAGTGACTG
GAGTGAAGTCGTAACAAGGTAGCCGTACTGGAAGGTGCGGCTGGATCACCTCCTT

>3847:emu_db:49250 ["3847:ncbi:49250 ['NC007942_91214122_Glycine_max_Streptophyta Glycine max 16S ribosomal RNA, chloroplast']"]

TCTCATGGAGAGTTCGATCCTGGCTCAGGATGAACGCTGGCGGCATGCCTTACAC
ATGCAAGTCGGACGGGAAGTGGTGTTTCCAGTGGCGGACGGGTGAGTAACGCGT
AAGAACCTACCCTTGGGAGGGGAACAACAGCTGGAAACGGCTGCTAATACCCCG
TAGGCTGAGGAGCAAAAGGAGGAATCCGCCCGAGGAGGGGCTCGCGTCTGATTA
GCTAGTTGGTGAGGCAATAGCTTACCAAGGCGATGATCAGTAGCTGGTCCGAGA
GGATGATCAGCCACACTGGGACTGAGACACGGCCCAGACTCCTACGGGAGGCAG
CAGTGGGGAATTTTCCGCAATGGGCGAAAGCCTGACGGAGCAATGCCGCGTGAA
GGTAGAAGGCCTACGGGTCATGAACTTCTTTTCCCGGAGAAGAAGCAATGACGGT

ATCCGGGGAATAAGCATCGGCTAACTCTGTGCCAGCAGCCGCGGTAAGACAGAG
GATGCAAGCGTTATCCGGAATGATTGGGCGTAAAGCGTCTGTAGGTGGCTTTTA
AGTTCGCCGTCAAATCCCAGGGCTCAACCCTGGACAGGCGGTGGAAACTACCAA
GCTGGAGTACGGTAGGGGCAGAGGGAATTTCCGGTGGAGCGGTGAAATGCGTAG
AGATCGGAAAGAACACCAACGGCGAAAGCACTCTGCTGGGCCGACACTGACACT
GAGAGACGAAAGCTAGGGGAGCGAATGGGATTAGATACCCCAGTAGTCCTAGCC
GTAAACGATGGATACTAGGCGCTGTGCGTATCGACCCGTGCAATGCTGTAGCTAA
CGCGTTAAGTATCCCGCCTGGGGAGTACGTTCGCAAGAATGAAACTCAAAGGAA
TTGACGGGGGCCCGCACAAGCGGTGGAGCATGTGGTTTAATTCGATGCAAAGCG
AAGAACCTTACCAGGGCTTGACATGCCGCGAATCCTCTTGAAAGAGAGGGGTGC
CTTCGGGAACGCGGACACAGGTGGTGCATGGCTGTCGTCAGCTCGTGCCGTAAGG
TGTTGGGTTAAGTCCCGCAACGAGCGCAACCCTCGTGTTTAGTTGCCAACATTTA
GTTTGGAACCCTGAGCAGACTGCCGGTGATAAGCCGGAGGAAGGTGAGGATGAC
GTCAAGTCATCATGCCCCTTATGCCCTGGGCGACACACGTGCTACAATGGACGGG
ACAAAGGATCGCGATCCCGCGAGGGTGAGCTAACTCCAAAAACCCGTCCTCAGTT
CGGATTGTAGGCTGCAACTCGCCTGCATGAAGCCGGAATCGCTAGTAATCGCCGG
TCAGCCATACGGCGGTGAATTCGTTCCCGGGCCTTGTACACACCGCCCGTCACAC
TATGGGAGCTGGCCATGCCCGAAGTCGTTACCTTAACCGCAAGGAGGGGGATGC
CGAAGGCAGGGCTAGTGACTGGAGTGAAGTCGTAACAAGGTAGCCGTACTGGAA
GGTGCGGCTGGATCACCTCCTTT

>3847:emu_db:49251 ["3847:ncbi:49251 ['NC_007942.99602.101092 Glycine max 16S ribosomal RNA, chloroplast']"]

TCTCATGGAGAGTTCGATCCTGGCTCAGGATGAACGCTGGCGGCATGCCTTACAC
ATGCAAGTCGGACGGGAAGTGGTGTTTCCAGTGGCGGACGGGTGAGTAACGCGT
AAGAACCTACCCTTGGGAGGGGAACAACAGCTGGAAACGGCTGCTAATACCCCG
TAGGCTGAGGAGCAAAAGGAGGAATCCGCCCGAGGAGGGGCTCGCGTCTGATTA
GCTAGTTGGTGAGGCAATAGCTTACCAAGGCGATGATCAGTAGCTGGTCCGAGA
GGATGATCAGCCACACTGGGACTGAGACACGGCCCAGACTCCTACGGGAGGCAG
CAGTGGGGAATTTTCCGCAATGGGCGAAAGCCTGACGGAGCAATGCCGCGTGAA
GGTAGAAGGCCTACGGGTCATGAACTTCTTTTCCCGGAGAAGAAGCAATGACGGT
ATCCGGGGAATAAGCATCGGCTAACTCTGTGCCAGCAGCCGCGGTAAGACAGAG
GATGCAAGCGTTATCCGGAATGATTGGGCGTAAAGCGTCTGTAGGTGGCTTTTA
AGTTCGCCGTCAAATCCCAGGGCTCAACCCTGGACAGGCGGTGGAAACTACCAA
GCTGGAGTACGGTAGGGGCAGAGGGAATTTCCGGTGGAGCGGTGAAATGCGTAG
AGATCGGAAAGAACACCAACGGCGAAAGCACTCTGCTGGGCCGACACTGACACT
GAGAGACGAAAGCTAGGGGAGCGAATGGGATTAGATACCCCAGTAGTCCTAGCC
GTAAACGATGGATACTAGGCGCTGTGCGTATCGACCCGTGCAATGCTGTAGCTAA
CGCGTTAAGTATCCCGCCTGGGGAGTACGTTCGCAAGAATGAAACTCAAAGGAA
TTGACGGGGGCCCGCACAAGCGGTGGAGCATGTGGTTTAATTCGATGCAAAGCG
AAGAACCTTACCAGGGCTTGACATGCCGCGAATCCTCTTGAAAGAGAGGGGTGC
CTTCGGGAACGCGGACACAGGTGGTGCATGGCTGTCGTCAGCTCGTGCCGTAAGG
TGTTGGGTTAAGTCCCGCAACGAGCGCAACCCTCGTGTTTAGTTGCCAACATTTA
GTTTGGAACCCTGAGCAGACTGCCGGTGATAAGCCGGAGGAAGGTGAGGATGAC
GTCAAGTCATCATGCCCCTTATGCCCTGGGCGACACACGTGCTACAATGGACGGG
ACAAAGGATCGCGATCCCGCGAGGGTGAGCTAACTCCAAAAACCCGTCCTCAGTT
CGGATTGTAGGCTGCAACTCGCCTGCATGAAGCCGGAATCGCTAGTAATCGCCGG
TCAGCCATACGGCGGTGAATTCGTTCCCGGGCCTTGTACACACCGCCCGTCACAC
TATGGGAGCTGGCCATGCCCGAAGTCGTTACCTTAACCGCAAGGAGGGGGATGC

CGAAGGCAGGGCTAGTGACTGGAGTGAAGTCGTAACAAGGTAGCCGTACTGGAA
GGTGCGGCTGGATCACCTCCTTT

>3847:emu_db:49252 ["3847:ncbi:49252 ['NC_007942.134302.135792 Glycine max 16S ribosomal RNA, chloroplast']"]

TCTCATGGAGAGTTCGATCCTGGCTCAGGATGAACGCTGGCGGCATGCCTTACAC
ATGCAAGTCGGACGGGAAGTGGTGTTTCCAGTGGCGGACGGGTGAGTAACGCGT
AAGAACCTACCCTTGGGAGGGGAACAACAGCTGGAAACGGCTGCTAATACCCCG
TAGGCTGAGGAGCAAAAGGAGGAATCCGCCCGAGGAGGGGCTCGCGTCTGATTA
GCTAGTTGGTGAGGCAATAGCTTACCAAGGCGATGATCAGTAGCTGGTCCGAGA
GGATGATCAGCCACACTGGGACTGAGACACGGCCCAGACTCCTACGGGAGGCAG
CAGTGGGGAATTTTCCGCAATGGGCGAAAGCCTGACGGAGCAATGCCGCGTGAA
GGTAGAAGGCCTACGGGTCATGAACTTCTTTTCCCGGAGAAGAAGCAATGACGGT
ATCCGGGGAATAAGCATCGGCTAACTCTGTGCCAGCAGCCGCGGTAAGACAGAG
GATGCAAGCGTTATCCGGAATGATTGGGCGTAAAGCGTCTGTAGGTGGCTTTTTA
AGTTCGCCGTCAAATCCCAGGGCTCAACCCTGGACAGGCGGTGGAAACTACCAA
GCTGGAGTACGGTAGGGGCAGAGGGAATTTCCGGTGGAGCGGTGAAATGCGTAG
AGATCGGAAAGAACACCAACGGCGAAAGCACTCTGCTGGGCCGACACTGACACT
GAGAGACGAAAGCTAGGGGAGCGAATGGGATTAGATACCCCAGTAGTCCTAGCC
GTAAACGATGGATACTAGGCGCTGTGCGTATCGACCCGTGCAATGCTGTAGCTAA
CGCGTTAAGTATCCCGCCTGGGGAGTACGTTCGCAAGAATGAAACTCAAAGGAA
TTGACGGGGGCCCGCACAAGCGGTGGAGCATGTGGTTTAATTCGATGCAAAGCG
AAGAACCTTACCAGGGCTTGACATGCCGCGAATCCTCTTGAAAGAGAGGGGTGC
CTTCGGGAACGCGGACACAGGTGGTGCATGGCTGTCGTCAGCTCGTGCCGTAAGG
TGTTGGGTTAAGTCCCGCAACGAGCGCAACCCTCGTGTTTAGTTGCCAACATTTA
GTTTGGAACCCTGAGCAGACTGCCGGTGATAAGCCGGAGGAAGGTGAGGATGAC
GTCAAGTCATCATGCCCCTTATGCCCTGGGCGACACACGTGCTACAATGGACGGG
ACAAAGGATCGCGATCCCGCGAGGGTGAGCTAACTCCAAAAACCCGTCCTCAGTT
CGGATTGTAGGCTGCAACTCGCCTGCATGAAGCCGGAATCGCTAGTAATCGCCGG
TCAGCCATACGGCGGTGAATTCGTTCCCGGGCCTTGTACACACCGCCCGTCACAC
TATGGGAGCTGGCCATGCCCGAAGTCGTTACCTTAACCGCAAGGAGGGGGATGC
CGAAGGCAGGGCTAGTGACTGGAGTGAAGTCGTAACAAGGTAGCCGTACTGGAA
GGTGCGGCTGGATCACCTCCTTT

>3847:emu_db:49253 ["3847:ncbi:49253 ['M21402.1.1277_U Glycine max 16S ribosomal RNA, chloroplast']"]

GAATTCGTTCCCGGGCCTTGTACACACCGCCCGTCACACTATGGGAGCTGGCCAT
GCCGAAGTCGTTACCTTAACCGCAAGAGGGGGATGCCGAAGGCAGGCTAGTGAC
TGGAGTGAAGTCGTAACAAGGTAGCCGTACTGGAAGGTGCGGCTGGATCACCTC
CTTTTCAGGGAGAGCTAATGCTTGTTGGGTAGTTTAGTTTGACACTGCTTCACACC
CAAAAAGAAGCGAGTTATGTCTGAGTCAAATTTGGAGATGGAAGTCTTCTTTCGT
TTCTCGATGGTGAAGTAAGACTAAACTCATGAGCTTATTATCCTAGGTCGGAACA
AGTTGATAGGAGCTACTTTTTTCACCCCCATCCATGTCGCCACACGGGGGCGACA
TGGATGGGGGTGAAAAAAGGAAAGAGAGGGATGGGGTTTCTCTTGCTTTTGGCA
TAGCGGGCCCCGGCGGGAGGCCCGCACGACGGGCTATTAGCTCAGTGGTAGAGC
GCGCCCCTGATAATTGCGTTCACGGGCGAGGTCTCTGGTTCAAGTCCAAGATGGC
CCAGCTGCGTCAAGGAAAAGAATAGAAAACTGACTTGACTCCTTCATGCATGCTC
CACTCGGCTCGGGGGGATATAGCTCAGTTGGTAGAGCTCCGCTCTTGCAATTGGG
CCTGTGGCGGATGTCAGCGGTTCGAGTCCGCTTATCTCCAACTCGTGAACTTAGTC

GATACAAAGCTATATGATAGCACTTCCATTTTTCCGATTCGGCAGTTTGGTCTATG
CTATGATTTATCATTCATGGACGTTGATAAGATCCTTCCATCTAGCAGCACCTTAG
GATGCATAGCCTTAAAGTTAAGGGCGAGGTTCAAACGAAGAAAGGCTTATGGTG
GATACCTAGGCACCCAGAGACGAGGAAGGGCGTAGTAAGCGACGAAATGCTTCG
GGGAGTTGAAAATAAGCGTAGATCCGGGGATTCCCGATATAGGTCAACCTTTCGA
ACTGCTGCTGAATCCACGGGCAGGCAAGAGACAACTTGGTGAACTGAAACATCTT
AGTAGCCAGAGGAAAAGAAAGCAAAAGCGATTCCCGTAGTAGCGGCGAGCGAA
ATGGGAGCAGCCTAAACCGTGAAAACGGGGTTGTGGGAGAGCTATACAAGTGTC
GTGCTGCTAGGCGAAGCAGCAGAGAATGCTGCACCCTAGATGGCGAGAGTCCAG
TAGCCGAAAGCATCACTGCTTACGCTCTGACCCGAGTAGCATAGGGCACGTGGAA
TCCCGTGTGAATCAGCAA

>3847:emu_db:49254 ["3847:ncbi:49254 ['ACUP01002633.86307.87624_U Glycine max 16S ribosomal RNA, chloroplast']"]

ATTTAGTTCTATTACTTAATTATTACAATTAGTTTATTATATGGATAATAAGGACT
GAAGAGTGGTTTCATTGCTCAATTAGTTGTTACAACAGAAAGAGAAAAATAAAC
AAAATCTTTAATTATTTATATAATTTTTATAGTTTCTAAACTTATCTATTTTAGTCT
TTATATTTAATAAGTAGATTTTTTAGTTTTTGAAGTTTACATTTTAATTTTCATAAA
ATCTTTGTTATTAAAATTTTTTAATTCACTTATTAAAATTTTAATTCCCAAAAGGC
CCATGTTTATGGAATAAGCATTGGTTAACTCTATGTGAGCAGTTGTGATAAGATA
AAGGATGCAAGTGTTATCCGGAATGATTGGGCGTAAAGCGTCTGTAGGTGACTTT
TTAAGTTTGCTGTCAAATCCCAGGGCTCAACCTTGGACAGGTGGTGAAAACTACC
AAGTTGGAGTACGGTAGGGGTAGAGGAAATTTCCGGTGGAGCGGTGAAATGCGT
AGAGATCGAAAAGAACACCAATGGCGAAAGCACTCTGTTGGGCCGACACTGACA
ATGAGAGACAAAAGCTAGGGGAACGAATGGGATTAGATACCCCAGTAGTCCTAG
CCGTAAACGATGGATACTAGGCGCTATGTGTATCAACCCGTGCAATGTTGTAGCT
AACGCGTTAAGTATCCCGCTTGGGGAGAACGTTCACAAGAATGAAACTCAAAGG
AATTGATGGGGGGCCGCACAAGCGTTGGAGCATGTTGTTTTAATTCGATGCAAAG
CAAAGAACTTTACCCTGGCTTGATATGTTGTGAATCCTCTTGAAAAAAAGGGGTG
CCTTCGGGGACCTAGACACAGGTGGTGCATGACTATCATCAGCTCGTGCCATAAG
GTGTTGGGTTAAGTTCGCAACGAGCGCAACCCTCATGTTTAGTTGCCAACATTGA
GTTTAGAACCCTGAGCAGACTGTCAGTGATAAGCCGGAGGAAGGTGAGGATGAC
ATCAAGTCATCATGCCCCTTATGACACGTGCTACACTGGATGTGACAAAGGATCA
TGATCCCGCAAGGGTGAACTAGCTCCAAAAACTCGTCCTTAGTTCGGATTATAGA
CTGCAACTCACCTGCATGAAGCCGAAATCGCTAGTAATCGCCAGTCAGTCATACG
GCGACGAATTCATTCCCGAGGCCTTGTACACACCGCCCGTCATACTATGGGAGCT
GACCATGCCAGAAGTCGTTACCTTAACCGCAAGGAGGGGGATGCCAAAGGCAAG
GTTAGTGACTGAAGTGAAGTCGTAATAAGATAGTCGTACTGAAAGGTGTGGTTG

>3847:emu_db:49255 ["3847:ncbi:49255 ['X07675.10.1479_U Glycine max 16S ribosomal RNA, chloroplast']"]

TCTCATGGAGAGTTCGATCCTGGCTCAGGATGAACGCTGGCGGCATGCCTTACAC
ATGCAAGTCGGACGGGAAGTGGTGTTTCCAGTGGCGGACGGGTGAGTAACGCGT
AAGAACCTACCCTTGGGAGGGGAACAACAGCTGGAAACGGCTGCTAATACCCCG
TAGGCTGAGGAGCAAAAGGAGAATCCGCCCGAGGAGGGGCTCGCGTCTGATTAG
CTAGTTGGTGAGGCATAGCTTACCAAGGCGATGATCAGTAGCTGGTCCGAGAGAT
GATCAGCCACACTGGGACTGAGACGAGGCCCAGACTCTTACGGGAGGCAGCAGT
GGGGAATTTTCCGCAATGGCGAAAGCTGACGGAGCAATGCCGCGTGAAGGTAGA
AGGCCTACGGTCATGAACTTCTTTTCCCGGAGAAGAAGCAATGACGGTGATCCGG

GGAATAAGCATCGGCTAACTCTGTGCCAGCGGCCGCGGTAAGACAGAGGATGCA
AGCGTTATCCGGAATGATTGGGCGAAAGGTCTGTAGGTGGCTTTTTAAGTTCGCC
GTCAAATCCCAGGGCTCAACCCTGGACAGGCGGTGGAAACTACCAAGCTGGAGT
ACGGTAGGGGCAGAGGGAATTTCCGGTGGAGCGGTGAAATGCGTAGAGATCGGA
AAGAACACCAACGGCGAAAGCACTCTGCTGGGCCGACACTGACACTGAGAGACG
AAACTTAGGGGAGCGAATGGGATTAGATACCCCAGTAGTCCTAGCCGTAAACGA
TGGATACTAGGCGCTGTGCGTATCGACCCGTGCAATGCTGTACTAACGCGTTAAG
TATCCCGCCTGGGGAGTACGTTCGCAAGAATGAAACTCAAAGGAATTGACGGGG
GCCGCACAAGCGGTGGAGCATGTGGTTTAATTCGATGCAAAGCGAAGAACCTTA
CCAGGGCTTGACATGCCGCGAATCCTCTTGAAAGAGAGGGGTGCTTCGGGAACG
CGGACACAGGTGGTGCATGGCTGTCGTCAGCTCGTGCCGTAAGGTGTTGGGTTAA
GTCCCGCAACGAGCGCAACCCTCGTGTTTAGTTGCCAACATTTAGTTTGGAACCC
TGAGCAGACTGCCGGTGATAAGCCGGAGGAAGGTGAGGATGACGTCGAAGTCAT
CATGCCCCTTATGCCCTGGGCGACACACGTGCTACAATGGACAAAGGATCGCGAT
CCCGCGAGGTGAGCTAACTCCAAAAACCCGTCCTCAGTTCGGATTGTAGGCTGCA
ACTCGCCTGCATGAAGCCGGAATCGCTAGTAATCGCCGGTCAGCCATACGGCGGT
GAATTCGTTCCCGGGCCTTGTACACACCGCCCGTCACACTATGGGAGCTGGCCAT
GCCGAAGTCGTTACCTTAACCGCAAGAGGGGGATGCCGAAGGGGCTAGTGACTG
GAGTGAAGTCGTAACAAGGTAGCCGTACTGGAAGGTGCGGCTGGATCACCTCCTT

>3847:emu_db:49256 ["3847:ncbi:49256 ['DQ317523.99602.101092_U Glycine max 16S ribosomal RNA, chloroplast']"]

TCTCATGGAGAGTTCGATCCTGGCTCAGGATGAACGCTGGCGGCATGCCTTACAC
ATGCAAGTCGGACGGAAGTGGTGTTTCCAGTGGCGGACGGGTGAGTAACGCGT
AAGAACCTACCCTTGGGAGGGGAACAACAGCTGGAAACGGCTGCTAATACCCCG
TAGGCTGAGGAGCAAAAGGAGGAATCCGCCCGAGGAGGGGCTCGCGTCTGATTA
GCTAGTTGGTGAGGCAATAGCTTACCAAGGCGATGATCAGTAGCTGGTCCGAGA
GGATGATCAGCCACACTGGGACTGAGACACGGCCCAGACTCCTACGGGAGGCAG
CAGTGGGGAATTTTCCGCAATGGGCGAAAGCCTGACGGAGCAATGCCGCGTGAA
GGTAGAAGGCCTACGGGTCATGAACTTCTTTTCCCGGAGAAGAAGCAATGACGGT
ATCCGGGGAATAAGCATCGGCTAACTCTGTGCCAGCAGCCGCGGTAAGACAGAG
GATGCAAGCGTTATCCGGAATGATTGGGCGTAAAGCGTCTGTAGGTGGCTTTTTA
AGTTCGCCGTCAAATCCCAGGGCTCAACCCTGGACAGGCGGTGGAAACTACCAA
GCTGGAGTACGGTAGGGGCAGAGGGAATTTCCGGTGGAGCGGTGAAATGCGTAG
AGATCGGAAAGAACACCAACGGCGAAAGCACTCTGCTGGGCCGACACTGACACT
GAGAGACGAAAGCTAGGGGAGCGAATGGGATTAGATACCCCAGTAGTCCTAGCC
GTAAACGATGGATACTAGGCGCTGTGCGTATCGACCCGTGCAATGCTGTAGCTAA
CGCGTTAAGTATCCCGCCTGGGGAGTACGTTCGCAAGAATGAAACTCAAAGGAA
TTGACGGGGGCCCGCACAAGCGGTGGAGCATGTGGTTTAATTCGATGCAAAGCG
AAGAACCTTACCAGGGCTTGACATGCCGCGAATCCTCTTGAAAGAGAGGGGTGC
CTTCGGGAACGCGGACACAGGTGGTGCATGGCTGTCGTCAGCTCGTGCCGTAAGG
TGTTGGGTTAAGTCCCGCAACGAGCGCAACCCTCGTGTTTAGTTGCCAACATTTA
GTTTGGAACCCTGAGCAGACTGCCGGTGATAAGCCGGAGGAAGGTGAGGATGAC
GTCAAGTCATCATGCCCCTTATGCCCTGGGCGACACACGTGCTACAATGGACGGG
ACAAAGGATCGCGATCCCGCGAGGGTGAGCTAACTCCAAAAACCCGTCCTCAGTT
CGGATTGTAGGCTGCAACTCGCCTGCATGAAGCCGGAATCGCTAGTAATCGCCGG
TCAGCCATACGGCGGTGAATTCGTTCCCGGGCCTTGTACACACCGCCCGTCACAC
TATGGGAGCTGGCCATGCCCGAAGTCGTTACCTTAACCGCAAGGAGGGGGATGC
CGAAGGCAGGGCTAGTGACTGGAGTGAAGTCGTAACAAGGTAGCCGTACTGGAA
GGTGCGGCTGGATCACCTCCTTT

>3847:emu_db:49257 ["3847:ncbi:49257 ['DQ317523.134302.135792_U Glycine max 16S ribosomal RNA, chloroplast']"]

TCTCATGGAGAGTTCGATCCTGGCTCAGGATGAACGCTGGCGGCATGCCTTACAC
ATGCAAGTCGGACGGGAAGTGGTGTTTCCAGTGGCGGACGGGTGAGTAACGCGT
AAGAACCTACCCTTGGGAGGGGAACAACAGCTGGAAACGGCTGCTAATACCCCG
TAGGCTGAGGAGCAAAAGGAGGAATCCGCCCGAGGAGGGGCTCGCGTCTGATTA
GCTAGTTGGTGAGGCAATAGCTTACCAAGGCGATGATCAGTAGCTGGTCCGAGA
GGATGATCAGCCACACTGGGACTGAGACACGGCCCAGACTCCTACGGGAGGCAG
CAGTGGGGAATTTTCCGCAATGGGCGAAAGCCTGACGGAGCAATGCCGCGTGAA
GGTAGAAGGCCTACGGGTCATGAACTTCTTTTCCCGGAGAAGAAGCAATGACGGT
ATCCGGGGAATAAGCATCGGCTAACTCTGTGCCAGCAGCCGCGGTAAGACAGAG
GATGCAAGCGTTATCCGGAATGATTGGGCGTAAAGCGTCTGTAGGTGGCTTTTTA
AGTTCGCCGTCAAATCCCAGGGCTCAACCCTGGACAGGCGGTGGAAACTACCAA
GCTGGAGTACGGTAGGGGCAGAGGGAATTTCCGGTGGAGCGGTGAAATGCGTAG
AGATCGGAAAGAACACCAACGGCGAAAGCACTCTGCTGGGCCGACACTGACACT
GAGAGACGAAAGCTAGGGGAGCGAATGGGATTAGATACCCCAGTAGTCCTAGCC
GTAAACGATGGATACTAGGCGCTGTGCGTATCGACCCGTGCAATGCTGTAGCTAA
CGCGTTAAGTATCCCGCCTGGGGAGTACGTTCGCAAGAATGAAACTCAAAGGAA
TTGACGGGGGCCCGCACAAGCGGTGGAGCATGTGGTTTAATTCGATGCAAAGCG
AAGAACCTTACCAGGGCTTGACATGCCGCGAATCCTCTTGAAAGAGAGGGGTGC
CTTCGGGAACGCGGACACAGGTGGTGCATGGCTGTCGTCAGCTCGTGCCGTAAGG
TGTTGGGTTAAGTCCCGCAACGAGCGCAACCCTCGTGTTTAGTTGCCAACATTTA
GTTTGGAACCCTGAGCAGACTGCCGGTGATAAGCCGGAGGAAGGTGAGGATGAC
GTCAAGTCATCATGCCCCTTATGCCCTGGGCGACACACGTGCTACAATGGACGGG
ACAAAGGATCGCGATCCCGCGAGGGTGAGCTAACTCCAAAAACCCGTCCTCAGTT
CGGATTGTAGGCTGCAACTCGCCTGCATGAAGCCGGAATCGCTAGTAATCGCCGG
TCAGCCATACGGCGGTGAATTCGTTCCCGGGCCTTGTACACACCGCCCGTCACAC
TATGGGAGCTGGCCATGCCCGAAGTCGTTACCTTAACCGCAAGGAGGGGGATGC
CGAAGGCAGGGCTAGTGACTGGAGTGAAGTCGTAACAAGGTAGCCGTACTGGAA
GGTGCGGCTGGATCACCTCCTTT

>3847:emu_db:49258 ["3847:ncbi:49258 ['CQ828061.450.1763_U Glycine max 16S ribosomal RNA, chloroplast']"]

AGAGTTCGATCCTGGCTCAGGATGAACGCTGGCGGCATGCCTTACACATGCAAGT
CGGACGGGAAGTGGTGTTTCCAGTGGCGGACGGGTGAGTAACGCGTAAGAACCT
ACCCTTGGGAGGGGAACAACAGCTGGAAACGGCTGCTAATACCCCGTAGGCTGA
GGAGCAAAAGGAGGAATCCGCCCGAGGAGGGGCTCGCGTCTGATTAGCTAGTTG
GTGAGGCAATAGCTTACCAAGGCGATGATCAGTAGCTGGTCCGAGAGGATGATC
AGCCACACTGGGACTGAGACACGGCCCAGACTCCTACGGGAGGCAGCAGTGGGG
AATTTTCCGCAATGGGCGAAAGCCTGACGGAGCAATGCCGCGTGAAGGTAGAAG
GCCTACGGGTCATGAACTTCTTTTCCCGGAGAAGAAGCAATGACGGTATCCGGGG
AATAAGCATCGGCTAACTCTGTGCCAGCAGCCGCGGTAAGACAGAGGATGCAAG
CGTTATCCGGAATGATTGGGCGTAAAGCGTCTGTAGGTGGCTTTTTAAGTTCGCC
GTCAAATCCCAGGGCTCAACCCTGGACAGGCGGTGGAAACTACCAAGCTGGAGT
ACGGTAGGGGCAGAGGGAATTTCCGGTGGAGCGGTGAAATGCGTAGAGATCGGA
AAGAACACCAACGGCGAAAGCACTCTGCTGGGCCGACACTGACACTGAGAGACG
AAAGCTAGGGGAGCGAATGGGATTAGATACCCCAGTAGTCCTAGCCGTAAACGA
TGGATACTAGGCGCTGTGCGTATCGACCCGTGCAATGCTGTAGCTAACGCGTTAA
GTATCCCGCCTGGGGAGTACGTTCGCAAGAATGAAACTCAAAGGAATTGACGGG

GGCCCGCACAAGCGGTGGAGCATGTGGTTTAATTCGATGCAAAGCGAAGAACCT
TACCAGGGCTTGACATGCCGCGAATCCTCTTGAAAGAGAGGGGTGCCTTCGGGAA
CGCGGACACAGGTGGTGCATGGCTGTCGTCAGCTCGTGCCGTAAGGTGTTGGGTT
AAGTCCCGCAACGAGCGCAACCCTCGTGTTTAGTTGCCAACATTTAGTTTGGAAC
CCTGAGCAGACTGCCGGTGATAAGCCGGAGGAAGGTGAGGATGACGTCAAGTCA
TCATGCCCCTTATGCCCTGGGCGACACACGTGCTACAATGGACGGGACAAAGGAT
CGCGATCCCGCGAGGGTGAGCTAACTCCAAAAACCCGTCCTCAGTTCGGATTGTA
GGCTGCAACTCGCCTGCATGAAGCCGGAATCGCTAGTAATCGCCGGTCAGCCATA
CGGCGGTG

>4232:emu_db:49259 ["4232:ncbi:49259 ['NC_007977.99224.100714 Helianthus annuus 16S ribosomal RNA, chloroplast']"]

TCTCATGGAGAGTTCGATCCTGGCTCAGGATGAACGCTGGCGGCATGCTTAACAC
ATGCAAGTCGGACGGGAAGTGGTGTTTCCAGTGGCGGACGGGTGAGTAACGCGT
AAGAACCTGCCCTTGGGAGGGGAACAACAGCTGGAAACGGCTGCTAATACCCCG
TAGGCTGAGGAGCAAAAGGAGGAATCCGCCCGAGGAGGGGCTCGCGTCTGATTA
GCTAGTTGGTGAGGTAATAGCTTACCAAGGCGATGATCAGTAGCTGGTCCGAGAG
GATGATCAGCCACACTGGGACTGAGACACGGCCCAGACTCCTACGGGAGGCAGC
AGTGGGGAATTTTCCGCAATGGGCGAAAGCCTGACGGAGCAATGCCGCGTGGAG
GTAGAAGGCCCACGGGTCGTGAACTTCTTTTCCCGGAGAAGAAGCAATGACGGT
ATCTGGGGAATAAGCATCGGCTAACTCTGTGCCAGCAGCCGCGGTAATACAGAG
GATGCAAGCGTTATCCGGAATGATTGGGCGTAAAGCGTCTGTAGGTGGCTTTTTA
AGTCCGCCGTCAAATCCCAGGGCTCAACTCTGGACAGGCGGTGGAAACTACCAA
GCTGGAGTACGGTAGGGGCAGAGGGAATTTCCGGTGGAGCGGTGAAATGCGCAG
AGATCGGAAAGAACACCAACGGCGAAAGCACTCTGCTGGGCCGACACTGACACT
GAGAGACGAAAGCTAGGGGAGCGAATGGGATTAGATACCCCAGTAGTCCTAGCC
GTAAACGATGGATACTAGGCGCTGTGCGTATCGACCCGTGCAGTGCTGTAGCTAA
CGCGTTAAGTATCCCGCCTGGGGAGTACGTTCGCAAGAATGAAACTCAAAGGAA
TTGACGGGGGCCCGCACAAGCGGTGGAGCATGTGGTTTAATTCGATGCAAAGCG
AAGAACCTTACCAGGGCTTGACATGCCGCGAATCCTCTTGAAAGAGAGGGGTGC
CTTCGGGAACGCGGACACAGGTGGTGCATGGCTGTCGTCAGCTCGTGCCGTAAGG
TGTTGGGTTAAGTCCCGCAACGAGCGCAACCCTCGTGTTTAGTTGCCATCATTGA
GTTTGGAACCCTGAACAGACTGCCGGTGATAAGCCGGAGGAAGGTGAGGATGAC
GTCAAGTCATCATGCCCCTTATGCCCTGGGCGACACACGTGCTACAATGGCCGGG
ACAAAGGGTCGCGATCCCGCGAGGGTGAGCTAACTCCAAAAACCCGTCCTCAGTT
CGGATTGCAGGCTGCAACTCGCCTGCATGAAGCCGGAATCGCTAGTAATCGCCGG
TCAGCCATACGGCGGTGAATCCGTTCCCGGGCCTTGTACACACCGCCCGTCACAC
TATGGGAGCTGGCCATGCCCGAAGTCGTTACCTTAACCGCAAGGAGGGGGATGC
CGAAGGCAGGGCTAGTGACTGGAGTGAAGTCGTAACAAGGTAGCCGTACTGGAA
GGTGCGGCTGGATCACCTCCTTT

>4232:emu_db:49260 ["4232:ncbi:49260 ['NC_007977.133921.135411 Helianthus annuus 16S ribosomal RNA, chloroplast']"]

TCTCATGGAGAGTTCGATCCTGGCTCAGGATGAACGCTGGCGGCATGCTTAACAC
ATGCAAGTCGGACGGGAAGTGGTGTTTCCAGTGGCGGACGGGTGAGTAACGCGT
AAGAACCTGCCCTTGGGAGGGGAACAACAGCTGGAAACGGCTGCTAATACCCCG
TAGGCTGAGGAGCAAAAGGAGGAATCCGCCCGAGGAGGGGCTCGCGTCTGATTA
GCTAGTTGGTGAGGTAATAGCTTACCAAGGCGATGATCAGTAGCTGGTCCGAGAG
GATGATCAGCCACACTGGGACTGAGACACGGCCCAGACTCCTACGGGAGGCAGC

AGTGGGGAATTTTCCGCAATGGGCGAAAGCCTGACGGAGCAATGCCGCGTGGAG
GTAGAAGGCCCACGGGTCGTGAACTTCTTTTCCCGGAGAAGAAGCAATGACGGT
ATCTGGGGAATAAGCATCGGCTAACTCTGTGCCAGCAGCCGCGGTAATACAGAG
GATGCAAGCGTTATCCGGAATGATTGGGCGTAAAGCGTCTGTAGGTGGCTTTTTA
AGTCCGCCGTCAAATCCCAGGGCTCAACTCTGGACAGGCGGTGGAAACTACCAA
GCTGGAGTACGGTAGGGGCAGAGGGAATTTCCGGTGGAGCGGTGAAATGCGCAG
AGATCGGAAAGAACACCAACGGCGAAAGCACTCTGCTGGGCCGACACTGACACT
GAGAGACGAAAGCTAGGGGAGCGAATGGGATTAGATACCCCAGTAGTCCTAGCC
GTAAACGATGGATACTAGGCGCTGTGCGTATCGACCCGTGCAGTGCTGTAGCTAA
CGCGTTAAGTATCCCGCCTGGGGAGTACGTTCGCAAGAATGAAACTCAAAGGAA
TTGACGGGGGCCCGCACAAGCGGTGGAGCATGTGGTTTAATTCGATGCAAAGCG
AAGAACCTTACCAGGGCTTGACATGCCGCGAATCCTCTTGAAAGAGAGGGGTGC
CTTCGGGAACGCGGACACAGGTGGTGCATGGCTGTCGTCAGCTCGTGCCGTAAGG
TGTTGGGTTAAGTCCCGCAACGAGCGCAACCCTCGTGTTTAGTTGCCATCATTGA
GTTTGGAACCCTGAACAGACTGCCGGTGATAAGCCGGAGGAAGGTGAGGATGAC
GTCAAGTCATCATGCCCCTTATGCCCTGGGCGACACACGTGCTACAATGGCCGGG
ACAAAGGGTCGCGATCCCGCGAGGGTGAGCTAACTCCAAAAACCCGTCCTCAGTT
CGGATTGCAGGCTGCAACTCGCCTGCATGAAGCCGGAATCGCTAGTAATCGCCGG
TCAGCCATACGGCGGTGAATCCGTTCCCGGGCCTTGTACACACCGCCCGTCACAC
TATGGGAGCTGGCCATGCCCGAAGTCGTTACCTTAACCGCAAGGAGGGGGATGC
CGAAGGCAGGGCTAGTGACTGGAGTGAAGTCGTAACAAGGTAGCCGTACTGGAA
GGTGCGGCTGGATCACCTCCTTT

>4232:emu_db:49261 ["4232:ncbi:49261 ['DQ383815.99224.100714_U Helianthus annuus 16S ribosomal RNA, chloroplast']"]

TCTCATGGAGAGTTCGATCCTGGCTCAGGATGAACGCTGGCGGCATGCTTAACAC
ATGCAAGTCGGACGGGAAGTGGTGTTTCCAGTGGCGGACGGGTGAGTAACGCGT
AAGAACCTGCCCTTGGGAGGGGAACAACAGCTGGAAACGGCTGCTAATACCCCG
TAGGCTGAGGAGCAAAAGGAGGAATCCGCCCGAGGAGGGGCTCGCGTCTGATTA
GCTAGTTGGTGAGGTAATAGCTTACCAAGGCGATGATCAGTAGCTGGTCCGAGAG
GATGATCAGCCACACTGGGACTGAGACACGGCCCAGACTCCTACGGGAGGCAGC
AGTGGGGAATTTTCCGCAATGGGCGAAAGCCTGACGGAGCAATGCCGCGTGGAG
GTAGAAGGCCCACGGGTCGTGAACTTCTTTTCCCGGAGAAGAAGCAATGACGGT
ATCTGGGGAATAAGCATCGGCTAACTCTGTGCCAGCAGCCGCGGTAATACAGAG
GATGCAAGCGTTATCCGGAATGATTGGGCGTAAAGCGTCTGTAGGTGGCTTTTTA
AGTCCGCCGTCAAATCCCAGGGCTCAACTCTGGACAGGCGGTGGAAACTACCAA
GCTGGAGTACGGTAGGGGCAGAGGGAATTTCCGGTGGAGCGGTGAAATGCGCAG
AGATCGGAAAGAACACCAACGGCGAAAGCACTCTGCTGGGCCGACACTGACACT
GAGAGACGAAAGCTAGGGGAGCGAATGGGATTAGATACCCCAGTAGTCCTAGCC
GTAAACGATGGATACTAGGCGCTGTGCGTATCGACCCGTGCAGTGCTGTAGCTAA
CGCGTTAAGTATCCCGCCTGGGGAGTACGTTCGCAAGAATGAAACTCAAAGGAA
TTGACGGGGGCCCGCACAAGCGGTGGAGCATGTGGTTTAATTCGATGCAAAGCG
AAGAACCTTACCAGGGCTTGACATGCCGCGAATCCTCTTGAAAGAGAGGGGTGC
CTTCGGGAACGCGGACACAGGTGGTGCATGGCTGTCGTCAGCTCGTGCCGTAAGG
TGTTGGGTTAAGTCCCGCAACGAGCGCAACCCTCGTGTTTAGTTGCCATCATTGA
GTTTGGAACCCTGAACAGACTGCCGGTGATAAGCCGGAGGAAGGTGAGGATGAC
GTCAAGTCATCATGCCCCTTATGCCCTGGGCGACACACGTGCTACAATGGCCGGG
ACAAAGGGTCGCGATCCCGCGAGGGTGAGCTAACTCCAAAAACCCGTCCTCAGTT
CGGATTGCAGGCTGCAACTCGCCTGCATGAAGCCGGAATCGCTAGTAATCGCCGG
TCAGCCATACGGCGGTGAATCCGTTCCCGGGCCTTGTACACACCGCCCGTCACAC

TATGGGAGCTGGCCATGCCCGAAGTCGTTACCTTAACCGCAAGGAGGGGGATGC
CGAAGGCAGGGCTAGTGACTGGAGTGAAGTCGTAACAAGGTAGCCGTACTGGAA
GGTGCGGCTGGATCACCTCCTTT

>4232:emu_db:49262 ["4232:ncbi:49262 ['DQ383815.133921.135411_U Helianthus annuus 16S ribosomal RNA, chloroplast']"]

TCTCATGGAGAGTTCGATCCTGGCTCAGGATGAACGCTGGCGGCATGCTTAACAC
ATGCAAGTCGGACGGGAAGTGGTGTTTCCAGTGGCGGACGGGTGAGTAACGCGT
AAGAACCTGCCCTTGGGAGGGGAACAACAGCTGGAAACGGCTGCTAATACCCCG
TAGGCTGAGGAGCAAAAGGAGGAATCCGCCCGAGGAGGGGCTCGCGTCTGATTA
GCTAGTTGGTGAGGTAATAGCTTACCAAGGCGATGATCAGTAGCTGGTCCGAGAG
GATGATCAGCCACACTGGGACTGAGACACGGCCCAGACTCCTACGGGAGGCAGC
AGTGGGGAATTTTCCGCAATGGGCGAAAGCCTGACGGAGCAATGCCGCGTGGAG
GTAGAAGGCCCACGGGTCGTGAACTTCTTTTCCCGGAGAAGAAGCAATGACGGT
ATCTGGGGAATAAGCATCGGCTAACTCTGTGCCAGCAGCCGCGGTAATACAGAG
GATGCAAGCGTTATCCGGAATGATTGGGCGTAAAGCGTCTGTAGGTGGCTTTTTA
AGTCCGCCGTCAAATCCCAGGGCTCAACTCTGGACAGGCGGTGGAAACTACCAA
GCTGGAGTACGGTAGGGGCAGAGGGAATTTCCGGTGGAGCGGTGAAATGCGCAG
AGATCGGAAAGAACACCAACGGCGAAAGCACTCTGCTGGGCCGACACTGACACT
GAGAGACGAAAGCTAGGGGAGCGAATGGGATTAGATACCCCAGTAGTCCTAGCC
GTAAACGATGGATACTAGGCGCTGTGCGTATCGACCCGTGCAGTGCTGTAGCTAA
CGCGTTAAGTATCCCGCCTGGGGAGTACGTTCGCAAGAATGAAACTCAAAGGAA
TTGACGGGGGCCCGCACAAGCGGTGGAGCATGTGGTTTAATTCGATGCAAAGCG
AAGAACCTTACCAGGGCTTGACATGCCGCGAATCCTCTTGAAAGAGAGGGGTGC
CTTCGGGAACGCGGACACAGGTGGTGCATGGCTGTCGTCAGCTCGTGCCGTAAGG
TGTTGGGTTAAGTCCCGCAACGAGCGCAACCCTCGTGTTTAGTTGCCATCATTGA
GTTTGGAACCCTGAACAGACTGCCGGTGATAAGCCGGAGGAAGGTGAGGATGAC
GTCAAGTCATCATGCCCCTTATGCCCTGGGCGACACACGTGCTACAATGGCCGGG
ACAAAGGGTCGCGATCCCGCGAGGGTGAGCTAACTCCAAAAACCCGTCCTCAGTT
CGGATTGCAGGCTGCAACTCGCCTGCATGAAGCCGGAATCGCTAGTAATCGCCGG
TCAGCCATACGGCGGTGAATCCGTTCCCGGGCCTTGTACACACCGCCCGTCACAC
TATGGGAGCTGGCCATGCCCGAAGTCGTTACCTTAACCGCAAGGAGGGGGATGC
CGAAGGCAGGGCTAGTGACTGGAGTGAAGTCGTAACAAGGTAGCCGTACTGGAA
GGTGCGGCTGGATCACCTCCTTT

>4565:emu_db:49263 ["4565:ncbi:49263 ['AC232255.22276.23745_U Triticum aestivum 16S ribosomal RNA, chloroplast']"]

AGAGTTCGATCCTGGCTCAGGATGAACGCTGGCGGCATGCTTAACACATGCAAGT
CGAACGGGAAGTGGTGTTTCCAGTGGCGAACGGGTGAGTAACGCGTAAGAACCT
GCCCTTGGGAGGGGAACAACAACTGGAAACGGTTGCTAATACCCCGTAGGCTGA
GGAGCAAAAGGAGAAATCCGCCCAAGGAGGGGCTCGCGTCTGATTAGCTAGTTG
GTGAGGTAATAGCTTACCAAGGCGATGATCAGTAGCTGGTCCGAGAGGATGATC
AGCCACACTGGGACTGAGACACGGCCCAGACTCCTACGGGAGGCAGCAGTGGGG
AATTTTCCGCAATGGGCGAAAGCCTGACGGAGCAATGCCGCGTGGAGGTGGAAG
GCCTACGGGTCGTCAACTTCTTTTCTCGGAGAAGAAACAATGACGGTATCTGAGG
AATAAGCATCGGCTAACTCTGTGCCAGCAGCCGCGGTAAGACAGAGGATGCAAG
CGTTATCCGGAATGATTGGGCGTAAAGCGTCTGTAGGTGGCTTTTCAAGTCCGCC
GTCAAATCCCAGGGCTCAACCCTGGACAGGCGGTGGAAACTACCAAGCTGGAGT
ACGGTAGGGGCAGAGGGAATTTCCGGTGGAGCGGTGAAATGCATTGAGATCGGA

AAGAACACCAACGGCGAAAGCACTCTGCTGGGCCGACACTGACACTGAGAGACG
AAAGCTAGGGGAGCAAATGGGATTAGAGACCCCAGTAGTCCTAGCCGTAAACGA
TGGATACTAGGTGCTGTGCGACTCGACCCGTGCAGTGCTGTAGCTAACGCGTTAA
GTATCCCGCCTGGGGAGTACGTTCGCAAGAATGAAACTCAAAGGAATTGACGGG
GGCCCGCACAAGCGGTGGAGCATGTGGTTTAATTCGATGCAAAGCGAAGAACCT
TACCAGGGCTTGACATGCCGCGAATCCTCTTGAAAGAGAGGGGTGCCCTCGGGA
ACGCGGACACAGGTGGTGCATGGCTGTCGTCAGCTCGTGCCGTAAGGTGTTGGGT
TAAGTCTCGCAACGAGCGCAACCCTCGTGTTTAGTTGCCACTATGAGTTTGGAAC
CCTGAACAGACCGCCGGTGTTAAGCCGGAGGAAGGAGAGGATGAGGCCAAGTCA
TCATGCCCCTTATGCCCTGGGCGACACACGTGCTACAATGGGCGGGACAAAGGGT
CGCGATCTCGCGAGGGTGAGCTAACTCCAAAAACCCGTCCTCAGTTCGGATTGCA
GGCTGCAACTCGCCTGCATGAAGCAGGAATCGCTAGTAATCGCCGGTCAGCCATA
CGGCGGTGAATCCGTTCCCGGGCCTTGTACACACCGCCCGTCACACTATAGGAGC
TGGCCATGTTTGAAGTCATTACCCTTAACCGTAAGGAGGGGGATGCCTAAGGCTA
GGCTTGCGACTGGAGTGAAGTCGTAACAAGGTAGCCGTACTGGAAGGTGCGGCT
G

>4565:emu_db:49264 ["4565:ncbi:49264 ['AB042240.91051.92542_U Triticum aestivum 16S ribosomal RNA, chloroplast']"]

ATCTCATGGAGAGTTCGATCCTGGCTCAGGATGAACGCTGGCGGCATGCTTAACA
CATGCAAGTCGAACGGGAAGTGGTGTTTCCAGTGGCGAACGGGTGAGTAACGCG
TAAGAACCTGCCCTTGGGAGGGGAACAACAACTGGAAACGGTTGCTAATACCCC
GTAGGCTGAGGAGCAAAAGGAGAAATCCGCCCAAGGAGGGGCTCGCGTCTGATT
AGCTAGTTGGTGAGGTAATAGCTTACCAAGGCGATGATCAGTAGCTGGTCCGAGA
GGATGATCAGCCACACTGGGACTGAGACACGGCCCAGACTCCTACGGGAGGCAG
CAGTGGGGAATTTTCCGCAATGGGCGAAAGCCTGACGGAGCAATGCCGCGTGGA
GGTGGAAGGCCTACGGGTCGTCAACTTCTTTTCTCGGAGAAGAAACAATGACGGT
ATCTGAGGAATAAGCATCGGCTAACTCTGTGCCAGCAGCCGCGGTAAGACAGAG
GATGCAAGCGTTATCCGGAATGATTGGGCGTAAAGCGTCTGTAGGTGGCTTTTCA
AGTCCGCCGTCAAATCCCAGGGCTCAACCCTGGACAGGCGGTGGAAACTACCAA
GCTGGAGTACGGTAGGGGCAGAGGGAATTTCCGGTGGAGCGGTGAAATGCATTG
AGATCGGAAAGAACACCAACGGCGAAAGCACTCTGCTGGGCCGACACTGACACT
GAGAGACGAAAGCTAGGGGAGCAAATGGGATTAGAGACCCCAGTAGTCCTAGCC
GTAAACGATGGATACTAGGTGCTGTGCGACTCGACCCGTGCAGTGCTGTAGCTAA
CGCGTTAAGTATCCCGCCTGGGGAGTACGTTCGCAAGAATGAAACTCAAAGGAA
TTGACGGGGGCCCGCACAAGCGGTGGAGCATGTGGTTTAATTCGATGCAAAGCG
AAGAACCTTACCAGGGCTTGACATGCCGCGAATCCTCTTGAAAGAGAGGGGTGC
CCTCGGGAACGCGGACACAGGTGGTGCATGGCTGTCGTCAGCTCGTGCCGTAAGG
TGTTGGGTTAAGTCTCGCAACGAGCGCAACCCTCGTGTTTAGTTGCCACTATGAG
TTTGGAACCCTGAACAGACCGCCGGTGTTAAGCCGGAGGAAGGAGAGGATGAGG
CCAAGTCATCATGCCCCTTATGCCCTGGGCGACACACGTGCTACAATGGGCGGGA
CAAAGGGTCGCGATCTCGCGAGGGTGAGCTAACTCCAAAAACCCGTCCTCAGTTC
GGATTGCAGGCTGCAACTCGCCTGCATGAAGCAGGAATCGCTAGTAATCGCCGGT
CAGCCATACGGCGGTGAATCCGTTCCCGGGCCTTGTACACACCGCCCGTCACACT
ATAGGAGCTGGCCATGTTTGAAGTCATTACCCTTAACCGTAAGGAGGGGGATGCC
TAAGGCTAGGCTTGCGACTGGAGTGAAGTCGTAACAAGGTAGCCGTACTGGAAG
GTGCGGCTGGATCACCTCCTTT

>4565:emu_db:49265 ["4565:ncbi:49265 ['AJ239003.1.1482_U Triticum aestivum 16S ribosomal RNA, chloroplast']"]

GAGTTTGATCCTGGCTCAGGATGAACGCTGGCGGCATGCTTAACACATGCAAGTC
GAACGGGAAGTGGTGTTTCCAGTGGCGAACGGGTGAGTAACGCGTAAGAACCTG
CCCTTGGGAGGGGAACAACAACTGGAAACGGTTGCTAATACCCCGTAGGCTGAG
GAGCAAAAGGAGAAATCCGCCCAAGGAGGGGCTCGCGTCTGATTAGCTAGTTGG
TGAGGTAATAGCTTACCAAGGCGATGATCAGTAGCTGGTCCGAGAGGATGATCA
GCCACACTGGGACTGAGACACGGCCCAGACTCCTACGGGAGGCAGCAGTGGGGA
ATTTTCCGCAATGGGCGAAAGCCTGACGGAGCAATGCCGCGTGGAGGTGGAAGG
CCTACGGGTCGTCAACTTCTTTTCTCGGAGAAGAAACAATGACGGTATCTGAGGA
ATAAGCATCGGCTAACTCTGTGCCAGCAGCCGCGGTAAGACAGAGGATGCAAGC
GTTATCCGGAATGATTGGGCGTAAAGCGTCTGTAGGTGGCTTTTCAAGTCCGCCG
TCAAATCCCAGGGCTCAACCCTGGACAGGCGGTGGAAACTACCAAGCTGGAGTA
CGGTAGGGGCAGAGGGAATTTCCGGTGGAGCGGTGAAATGCATTGAGATCGGAA
AGAACACCAACGGCGAAAGCACTCTGCTGGGCCGACACTGACACTGAGAGACGA
AAGCTAGGGGAGCAAATGGGATTAGAGACCCCAGTAGTCCTAGCCGTAAACGAT
GGATACTAGGTGCTGTGCGACTCGACCCGTGCAGTGCTGTAGCTAACGCGTTAAG
TATCCCGCCTGGGGAGTACGTTCGCAAGAATGAAACTCAAAGGAATTGACGGGG
GCCCGCACAAGCGGTGGAGCATGTGGTTTAATTCGATGCAAAGCGAAGAACCTT
ACCAGGGCTTGACATGCCGCGAATCCTCTTGAAAGAGAGGGGTGCCCTCGGGAA
CGCGGACACAGGTGGTGCATGGCTGTCGTCAGCTCGTGCCGTAAGGTGTTGGGTT
AAGTCTCGCAACGAGCGCAACCCTTGTGTTTAGTTGCCACTATGAGTTTGGAACC
CTGAACAGACCGCCGGTGTTAAGCCGGAGGAAGGAGAGGATGAGGCCAAGTCAT
CATGCCCCTTATGCCCTGGGCGACACACGTGCTACAATGGGCGGGACAAAGGGTC
GCGATCTCGCGAGGGTGAGCTAACTCCAAAAACCCGTCCTCAGTTCGGATTGCAG
GCTGCAACTCGCCTGCATGAAGCAGGAATCGCTAGTAATCGCCGGTCAGCCATAC
GGCGGTGAATCCGTTCCCGGGCCTTGTACACACCGCCCGTCACACTATAGGAGCT
GGCCATGTTTGAAGTCATTACCCTTAACCGTAAGGAGGGGGATGCCTAAGGCTAG
GCTTGCGACTGGAGTGAAGTCGTAACAAGGTAGCCGTACTGGAAGGTGCGGCTG
GATCACCTCCTTT

>4565:emu_db:49266 ["4565:ncbi:49266 ['AB042240.122352.123842_U Triticum aestivum 16S ribosomal RNA, chloroplast']"]

TCTCATGGAGAGTTCGATCCTGGCTCAGGATGAACGCTGGCGGCATGCTTAACAC
ATGCAAGTCGAACGGGAAGTGGTGTTTCCAGTGGCGAACGGGTGAGTAACGCGT
AAGAACCTGCCCTTGGGAGGGGAACAACAACTGGAAACGGTTGCTAATACCCCG
TAGGCTGAGGAGCAAAAGGAGAAATCCGCCCAAGGAGGGGCTCGCGTCTGATTA
GCTAGTTGGTGAGGTAATAGCTTACCAAGGCGATGATCAGTAGCTGGTCCGAGAG
GATGATCAGCCACACTGGGACTGAGACACGGCCCAGACTCCTACGGGAGGCAGC
AGTGGGGAATTTTCCGCAATGGGCGAAAGCCTGACGGAGCAATGCCGCGTGGAG
GTGGAAGGCCTACGGGTCGTCAACTTCTTTTCTCGGAGAAGAAACAATGACGGTA
TCTGAGGAATAAGCATCGGCTAACTCTGTGCCAGCAGCCGCGGTAAGACAGAGG
ATGCAAGCGTTATCCGGAATGATTGGGCGTAAAGCGTCTGTAGGTGGCTTTTCAA
GTCCGCCGTCAAATCCCAGGGCTCAACCCTGGACAGGCGGTGGAAACTACCAAG
CTGGAGTACGGTAGGGGCAGAGGGAATTTCCGGTGGAGCGGTGAAATGCATTGA
GATCGGAAAGAACACCAACGGCGAAAGCACTCTGCTGGGCCGACACTGACACTG
AGAGACGAAAGCTAGGGGAGCAAATGGGATTAGAGACCCCAGTAGTCCTAGCCG
TAAACGATGGATACTAGGTGCTGTGCGACTCGACCCGTGCAGTGCTGTAGCTAAC
GCGTTAAGTATCCCGCCTGGGGAGTACGTTCGCAAGAATGAAACTCAAAGGAATT
GACGGGGGCCCGCACAAGCGGTGGAGCATGTGGTTTAATTCGATGCAAAGCGAA
GAACCTTACCAGGGCTTGACATGCCGCGAATCCTCTTGAAAGAGAGGGGTGCCCT
CGGGAACGCGGACACAGGTGGTGCATGGCTGTCGTCAGCTCGTGCCGTAAGGTGT

TGGGTTAAGTCTCGCAACGAGCGCAACCCTCGTGTTTAGTTGCCACTATGAGTTT
GGAACCCTGAACAGACCGCCGGTGTTAAGCCGGAGGAAGGAGAGGATGAGGCCA
AGTCATCATGCCCCTTATGCCCTGGGCGACACACGTGCTACAATGGGCGGGACAA
AGGGTCGCGATCTCGCGAGGGTGAGCTAACTCCAAAAACCCGTCCTCAGTTCGGA
TTGCAGGCTGCAACTCGCCTGCATGAAGCAGGAATCGCTAGTAATCGCCGGTCAG
CCATACGGCGGTGAATCCGTTCCCGGGCCTTGTACACACCGCCCGTCACACTATA
GGAGCTGGCCATGTTTGAAGTCATTACCCTTAACCGTAAGGAGGGGGATGCCTAA
GGCTAGGCTTGCGACTGGAGTGAAGTCGTAACAAGGTAGCCGTACTGGAAGGTG
CGGCTGGATCACCTCCTTT

>4565:emu_db:49267 ["4565:ncbi:49267 ['KC912694.91545.93036_U Triticum aestivum 16S ribosomal RNA, chloroplast']"]

ATCTCATGGAGAGTTCGATCCTGGCTCAGGATGAACGCTGGCGGCATGCTTAACA
CATGCAAGTCGAACGGGAAGTGGTGTTTCCAGTGGCGAACGGGTGAGTAACGCG
TAAGAACCTGCCCTTGGGAGGGGAACAACAACTGGAAACGGTTGCTAATACCCC
GTAGGCTGAGGAGCAAAAGGAGAAATCCGCCCAAGGAGGGGCTCGCGTCTGATT
AGCTAGTTGGTGAGGTAATAGCTTACCAAGGCGATGATCAGTAGCTGGTCCGAGA
GGATGATCAGCCACACTGGGACTGAGACACGGCCCAGACTCCTACGGGAGGCAG
CAGTGGGGAATTTTCCGCAATGGGCGAAAGCCTGACGGAGCAATGCCGCGTGGA
GGTGGAAGGCCTACGGGTCGTCAACTTCTTTTCTCGGAGAAGAAACAATGACGGT
ATCTGAGGAATAAGCATCGGCTAACTCTGTGCCAGCAGCCGCGGTAAGACAGAG
GATGCAAGCGTTATCCGGAATGATTGGGCGTAAAGCGTCTGTAGGTGGCTTTTCA
AGTCCGCCGTCAAATCCCAGGGCTCAACCCTGGACAGGCGGTGGAAACTACCAA
GCTGGAGTACGGTAGGGGCAGAGGGAATTTCCGGTGGAGCGGTGAAATGCATTG
AGATCGGAAAGAACACCAACGGCGAAAGCACTCTGCTGGGCCGACACTGACACT
GAGAGACGAAAGCTAGGGGAGCAAATGGGATTAGAGACCCCAGTAGTCCTAGCC
GTAAACGATGGATACTAGGTGCTGTGCGACTCGACCCGTGCAGTGCTGTAGCTAA
CGCGTTAAGTATCCCGCCTGGGGAGTACGTTCGCAAGAATGAAACTCAAAGGAA
TTGACGGGGGCCCGCACAAGCGGTGGAGCATGTGGTTTAATTCGATGCAAAGCG
AAGAACCTTACCAGGGCTTGACATGCCGCGAATCCTCTTGAAAGAGAGGGGTGC
CCTCGGGAACGCGGACACAGGTGGTGCATGGCTGTCGTCAGCTCGTGCCGTAAGG
TGTTGGGTTAAGTCTCGCAACGAGCGCAACCCTCGTGTTTAGTTGCCACTATGAG
TTTGGAACCCTGAACAGACCGCCGGTGTTAAGCCGGAGGAAGGAGAGGATGAGG
CCAAGTCATCATGCCCCTTATGCCCTGGGCGACACACGTGCTACAATGGGCGGGA
CAAAGGGTCGCGATCTCGCGAGGGTGAGCTAACTCCAAAAACCCGTCCTCAGTTC
GGATTGCAGGCTGCAACTCGCCTGCATGAAGCAGGAATCGCTAGTAATCGCCGGT
CAGCCATACGGCGGTGAATCCGTTCCCGGGCCTTGTACACACCGCCCGTCACACT
ATAGGAGCTGGCCATGTTTGAAGTCATTACCCTTAACCGTAAGGAGGGGGATGCC
TAAGGCTAGGCTTGCGACTGGAGTGAAGTCGTAACAAGGTAGCCGTACTGGAAG
GTGCGGCTGGATCACCTCCTTT

>4565:emu_db:49268 ["4565:ncbi:49268 ['NC_002762.122352.123842_U Triticum aestivum 16S ribosomal RNA, chloroplast']"]

TCTCATGGAGAGTTCGATCCTGGCTCAGGATGAACGCTGGCGGCATGCTTAACAC
ATGCAAGTCGAACGGGAAGTGGTGTTTCCAGTGGCGAACGGGTGAGTAACGCGT
AAGAACCTGCCCTTGGGAGGGGAACAACAACTGGAAACGGTTGCTAATACCCCG
TAGGCTGAGGAGCAAAAGGAGAAATCCGCCCAAGGAGGGGCTCGCGTCTGATTA
GCTAGTTGGTGAGGTAATAGCTTACCAAGGCGATGATCAGTAGCTGGTCCGAGAG
GATGATCAGCCACACTGGGACTGAGACACGGCCCAGACTCCTACGGGAGGCAGC

AGTGGGGAATTTTCCGCAATGGGCGAAAGCCTGACGGAGCAATGCCGCGTGGAG
GTGGAAGGCCTACGGGTCGTCAACTTCTTTTCTCGGAGAAGAAACAATGACGGTA
TCTGAGGAATAAGCATCGGCTAACTCTGTGCCAGCAGCCGCGGTAAGACAGAGG
ATGCAAGCGTTATCCGGAATGATTGGGCGTAAAGCGTCTGTAGGTGGCTTTTCAA
GTCCGCCGTCAAATCCCAGGGCTCAACCCTGGACAGGCGGTGGAAACTACCAAG
CTGGAGTACGGTAGGGGCAGAGGGAATTTCCGGTGGAGCGGTGAAATGCATTGA
GATCGGAAAGAACACCAACGGCGAAAGCACTCTGCTGGGCCGACACTGACACTG
AGAGACGAAAGCTAGGGGAGCAAATGGGATTAGAGACCCCAGTAGTCCTAGCCG
TAAACGATGGATACTAGGTGCTGTGCGACTCGACCCGTGCAGTGCTGTAGCTAAC
GCGTTAAGTATCCCGCCTGGGGAGTACGTTCGCAAGAATGAAACTCAAAGGAATT
GACGGGGGCCCGCACAAGCGGTGGAGCATGTGGTTTAATTCGATGCAAAGCGAA
GAACCTTACCAGGGCTTGACATGCCGCGAATCCTCTTGAAAGAGAGGGGTGCCCT
CGGGAACGCGGACACAGGTGGTGCATGGCTGTCGTCAGCTCGTGCCGTAAGGTGT
TGGGTTAAGTCTCGCAACGAGCGCAACCCTCGTGTTTAGTTGCCACTATGAGTTT
GGAACCCTGAACAGACCGCCGGTGTTAAGCCGGAGGAAGGAGAGGATGAGGCCA
AGTCATCATGCCCCTTATGCCCTGGGCGACACACGTGCTACAATGGGCGGGACAA
AGGGTCGCGATCTCGCGAGGGTGAGCTAACTCCAAAAACCCGTCCTCAGTTCGGA
TTGCAGGCTGCAACTCGCCTGCATGAAGCAGGAATCGCTAGTAATCGCCGGTCAG
CCATACGGCGGTGAATCCGTTCCCGGGCCTTGTACACACCGCCCGTCACACTATA
GGAGCTGGCCATGTTTGAAGTCATTACCCTTAACCGTAAGGAGGGGGATGCCTAA
GGCTAGGCTTGCGACTGGAGTGAAGTCGTAACAAGGTAGCCGTACTGGAAGGTG
CGGCTGGATCACCTCCTTT

>4565:emu_db:49269 ["4565:ncbi:49269 ['NC_002762.91051.92542_U Triticum aestivum 16S ribosomal RNA, chloroplast']"]

ATCTCATGGAGAGTTCGATCCTGGCTCAGGATGAACGCTGGCGGCATGCTTAACA
CATGCAAGTCGAACGGGAAGTGGTGTTTCCAGTGGCGAACGGGTGAGTAACGCG
TAAGAACCTGCCCTTGGGAGGGGAACAACAACTGGAAACGGTTGCTAATACCCC
GTAGGCTGAGGAGCAAAAGGAGAAATCCGCCCAAGGAGGGGCTCGCGTCTGATT
AGCTAGTTGGTGAGGTAATAGCTTACCAAGGCGATGATCAGTAGCTGGTCCGAGA
GGATGATCAGCCACACTGGGACTGAGACACGGCCCAGACTCCTACGGGAGGCAG
CAGTGGGGAATTTTCCGCAATGGGCGAAAGCCTGACGGAGCAATGCCGCGTGGA
GGTGGAAGGCCTACGGGTCGTCAACTTCTTTTCTCGGAGAAGAAACAATGACGGT
ATCTGAGGAATAAGCATCGGCTAACTCTGTGCCAGCAGCCGCGGTAAGACAGAG
GATGCAAGCGTTATCCGGAATGATTGGGCGTAAAGCGTCTGTAGGTGGCTTTTCA
AGTCCGCCGTCAAATCCCAGGGCTCAACCCTGGACAGGCGGTGGAAACTACCAA
GCTGGAGTACGGTAGGGGCAGAGGGAATTTCCGGTGGAGCGGTGAAATGCATTG
AGATCGGAAAGAACACCAACGGCGAAAGCACTCTGCTGGGCCGACACTGACACT
GAGAGACGAAAGCTAGGGGAGCAAATGGGATTAGAGACCCCAGTAGTCCTAGCC
GTAAACGATGGATACTAGGTGCTGTGCGACTCGACCCGTGCAGTGCTGTAGCTAA
CGCGTTAAGTATCCCGCCTGGGGAGTACGTTCGCAAGAATGAAACTCAAAGGAA
TTGACGGGGGCCCGCACAAGCGGTGGAGCATGTGGTTTAATTCGATGCAAAGCG
AAGAACCTTACCAGGGCTTGACATGCCGCGAATCCTCTTGAAAGAGAGGGGTGC
CCTCGGGAACGCGGACACAGGTGGTGCATGGCTGTCGTCAGCTCGTGCCGTAAGG
TGTTGGGTTAAGTCTCGCAACGAGCGCAACCCTCGTGTTTAGTTGCCACTATGAG
TTTGGAACCCTGAACAGACCGCCGGTGTTAAGCCGGAGGAAGGAGAGGATGAGG
CCAAGTCATCATGCCCCTTATGCCCTGGGCGACACACGTGCTACAATGGGCGGGA
CAAAGGGTCGCGATCTCGCGAGGGTGAGCTAACTCCAAAAACCCGTCCTCAGTTC
GGATTGCAGGCTGCAACTCGCCTGCATGAAGCAGGAATCGCTAGTAATCGCCGGT
CAGCCATACGGCGGTGAATCCGTTCCCGGGCCTTGTACACACCGCCCGTCACACT

ATAGGAGCTGGCCATGTTTGAAGTCATTACCCTTAACCGTAAGGAGGGGGATGCC
TAAGGCTAGGCTTGCGACTGGAGTGAAGTCGTAACAAGGTAGCCGTACTGGAAG
GTGCGGCTGGATCACCTCCTTT

>4565:emu_db:49270 ["4565:ncbi:49270 ['JV885546.3.1452 Triticum aestivum 16S ribosomal RNA, chloroplast']"]

AGAGTTCGATCCTGGCTCAGGATGAACGCTGGCGGCATGCTTAACACATGCAAGT
CGAACGGGAAGTGGTGTTTCCAGTGGCGAACGGGTGAGTAACGCGTAAGAACCT
GCCCTTGGGAGGGGAACAACAACTGGAAACGGTTGCTAATACCCCGTAGGCTGA
GGAGCAAAAGGAGAAATCCGCCCAAGGAGGGGCTCGCGTCTGATTAGCTAGTTG
GTGAGGTAATAGCTTACCAAGGCGATGATCAGTAGCTGGTCCGAGAGGATGATC
AGCCACACTGGGACTGAGACACGGCCCAGACTCCTACGGGAGGCAGCAGTGGGG
AATTTTCCGCAATGGGCGAAAGCCTGACGGAGCAATGCCGCGTGGAGGTGGAAG
GCCTACGGGTCGTCAACTTCTTTTCTCGGAGAAGAAACAATGACGGTATCTGAGG
AATAAGCATCGGCTAACTCTGTGCCAGCAGCCGCGGTAAGACAGAGGATGCAAG
CGTTATCCGGAATGATTGGGCGTAAAGCGTCTGTAGGTGGCTTTTCAAGTCCGCC
GTCAAATCCCAGGGCTCAACCCTGGACAGGCGGTGGAAACTACCAAGCTGGAGT
ACGGTAGGGGCAGAGGGAATTTCCGGTGGAGCGGTGAAATGCATTGAGATCGGA
AAGAACACCAACGGCGAAAGCACTCTGCTGGGCCGACACTGACACTGAGAGACG
AAAGCTAGGGGAGCAAATGGGATTAGAGACCCCAGTAGTCCTAGCCGTAAACGA
TGGATACTAGGTGCTGTGCGACTCGACCCGTGCAGTGCTGTAGCTAACGCGTTAA
GTATCCCGCCTGGGGAGTACGTTCGCAAGAATGAAACTCAAAGGAATTGACGGG
GGCCCGCACAAGCGGTGGAGCATGTGGTTTAATTCGATGCAAAGCGAAGAACCT
TACCAGGGCTTGACATGCCGCGAATCCTCTTGAAAGAGAGGGGTGCCCTCGGGA
ACGCGGACACAGGTGGTGCATGGCTGTCGTCAGCTCGTGCCGTAAGGTGTTGGGT
TAAGTCTCGCAACGAGCGCAACCCTCGTGTTTAGTTGCCACTATGAGTTTGGAAC
CCTGAACAGACCGCCGGTGTTAAGCCGGAGGAAGGAGAGGATGAGGCCAAGTCA
TCATGCCCCTTATGCCCTGGGCGACACACGTGCTACAATGGGCGGGACAAAGGGT
CGCGATCTCGCGAGGGTGAGCTAACTCCAAAAACCCGTCCTCAGTTCGGATTGCA
GGCTGCAACTCGCCTGCATGAAGCAGGAATCGCTAGTAATCGCCGGTCAGCCATA
CGGCGGTGAATCCGTTCCCGGGCCTTGTACACACCGCCCGTCACACTATAGGAGC
TGGCCATGTTTGAAGTCATTACCCTTAACCGTAAGGAGGGGGATGCCTAAGGCTA
GGCTTGCGACTGGAGTGAAGTCGTAACAAGGTAGC

>4565:emu_db:49271 ["4565:ncbi:49271 ['JP829337.2569.4303 Triticum aestivum 16S ribosomal RNA, chloroplast']"]

AGAGTTCGATCCTGGCTCAGGATGAACGCTGGCGGCATGCTTAACACATGCAAGT
CGAACGGGAAGTGGTGTTTCCAGTGGCGAACGGGTGAGTAACGCGTAAGAACCT
GCCCTTGGGAGGGGAACAACAACTGGAAACGGTTGCTAATACCCCGTAGGCTGA
GGAGCAAAAGGAGAAATCCGCCCAAGGAGGGGCTCGCGTCTGATTAGCTAGTTG
GTGAGGTAATAGCTTACCAAGGCGATGATCAGTAGCTGGTCCGAGAGGATGATC
AGCCACACTGGGACTGAGACACGGCCCAGACTCCTACGGGAGGCAGCAGTGGGG
AATTTTCCGCAATGGGCGAAAGCCTGACGGAGCAATGCCGCGTGGAGGTGGAAG
GCCTACGGGTCGTCAACTTCTTTTCTCGGAGAAGAAACAATGACGGTATCTGAGG
AATAAGCATCGGCTAACTCTGTGCCAGCAGCCGCGGTAAGACAGAGGATGCAAG
CGKTATCCGGAATGATTGGGCGTAAAGCGTCTGTAGGYGGCTTTTCAAGTCCGCC
GTCAAATCCCAGGGCTCAACCCTGGACAGGCGGTGGAAACTACCAAGCTGGAGT
ACGGTAGGGGCAGAGGGAATTTCCGGTGGAGCGGKGAAATGCATTGAGATCGGA
AAGAACACCAACGGCGAAAGCACTCTGCTGGGCCGACACTGACACTGAGAGACG

AAAGCYAGGGGAGCAAATGGGATTAGAGACMCMAGTAGTCCTAGCCGTAAACG
ATGGATACTAGGGGCKGKGCGACTCGMCMCGTGCAGTKCTGTAGCTAACGCGTT
AAGKATCCCGCCTGGGGAGTACGTTCGCAAGAATGAAACTCAAAGGAATTGACG
GGGGCCCGCAGGGAATTTCCGGTGGAGCGGTGAAATGCATTGAGATCGGAAAGA
ACACCAACGGCGAAAGCACTCTGCTGGGCCGACACTGACACTGAGAGACGAAAG
CTAGGGGAGCAAATGGGATTAGAGACCCCAGTAGTCCTAGCCGTAAACGATGGA
TACTAGGTGCTGTGCGACTCGACCCGTGCAGTGCTGTAGCTAACGCGTTAAGTAT
CCCGCCTGGGGAGTACGTTCGCAAGAATGAAACTCAAAGGAATTGACGGGGGCC
CGCACAAGCGGTGGAGCATGTGGTTTAATTCGATGCAAAGCGAAGAACCTTACC
AGGGCTTGACATGCCGCGAATCCTCTTGAAAGAGAGGGGTGCCCTCGGGAACGC
GGACACAGGTGGTGCGTGGCTGTCGTCAGCTCGTGCCGTAAGGTGTTGGGTTAAG
TCTCGCAACGAGCGCAACCCTCGTGTTTAGTTGCCACTATGAGTTTGGAACCCTG
AACAGACCGCCGGTGTTAAGCCGGAGGAAGGAGAGGATGAGGCCAAGTCATCAT
GCCCCTTATGCCCTGGGCGACACACGTGCTACAATGGGCGGGACAAAGGGTCGC
GATCTCGCGAGGGTGAGCTAACTCCAAAAACCCGTCCTCAGTTCGGATTGCAGGC
TGCAACKCGCCTGCATGAAGCAGGAATCGCTAGTAATCGCCGGYCAGCCATACG
GCGGKGAATCCGTTCCCGGGCCTTGTACACACCGCCCGTCACACTATAGGAGCTG
GCCATGTTTGAAGTCATTACCCTTAACCGTAAGGAGGGGGATGCCTAAGGCTAGG
CTTGCGACTGGAGTGAAGTCGKAACAAGGTAGCCGTACTGGRAGGTGCGGCTG

>4571:emu_db:49272 ["4571:ncbi:49272 ['AJ555400.1.1483_U Triticum turgidum 16S ribosomal RNA, chloroplast']"]

TGAGTTTGATCCTGGCTCAGGATGAACGCTGGCGGCATGCTTAACACATGCAAGT
CGAACGGGAAGTGGTGTTTCCAGTGGCGAACGGGTGAGTAACGCGTAAGAACCT
GCCCTTGGGAGGGGAACAACAACTGGAAACGGTTGCTAATACCCCGTAGGCTGA
GGAGCAAAAGGAGAAATCCGCCCAAGGAGGGGCTCGCGTCTGATTAGCTAGTTG
GTGAGGTAATAGCTTACCAAGGCGATGATCAGTAGCTGGTCCGAGAGGATGATC
AGCCACACTGGGACTGAGACACGGCCCAGACTCCTACGGGAGGCAGCAGTGGGG
AATTTTCCGCAATGGGCGAAAGCCTGACGGAGCAATGCCGCGTGGAGGTGGAAG
GCCTACGGGTCGTCAACTTCTTTTCTCGGAGAAGAAACAATGACGGTATCTGAGG
AATAAGCATCGGCTAACTCTGTGCCAGCAGCCGCGGTAAGACAGAGGATGCAAG
CGTTATCCGGAATGATTGGGCGTAAAGCGTCTGTAGGTGGCTTTTCAAGTCCGCC
GTCAAATCCCAGGGCTCAACCCTGGACAGGCGGTGGAAACTACCAAGCTGGAGT
ACGGTAGGGGCAGAGGGAATTTCCGGTGGAGCGGTGAAATGCATTGAGATCGGA
AAGAACACCAACGGCGAAAGCACTCTGCTGGGCCGACACTGACACTGAGAGACG
AAAGCTAGGGGAGCAAATGGGATTAGAGACCCCAGTAGTCCTAGCCGTAAACGA
TGGATACTAGGTGCTGTGCGACTCGACCCGTGCAGTGCTGTAGCTAACGCGTTAA
GTATCCCGCCTGGGGAGTACGTTCGCAAGAATGAAACTCAAAGGAATTGACGGG
GGCCCGCACAAGCGGTGGAGCATGTGGTTTAATTCGATGCAAAGCGAAGAACCT
TACCAGGGCTTGACATGCCGCGAATCCTCTTGAAAGAGAGGGGTGCCCTCGGGA
ACGCGGACACAGGTGGTGCATGGCTGTCGTCAGCTCGTGCCGTAAGGTGTTGGGT
TAAGTCTCGCAACGAGCGCAACCCTCGTGTTTAGTTGCCACTATGAGTTTGGAAC
CCTGAACAGACCGCCGGTGTTAAGCCGGAGGAAGGAGAGGATGAGGCCAAGTCA
TCATGCCCCTTATGCCCTGGGCGACACACGTGCTACAATGGGCGGGACAAAGGGT
CGCGATCTCGCGAGGGTGAGCTAACTCCAAAAACCCGTCCTCAGTTCGGATTGCA
GGCTGCAACTCGCCTGCATGAAGCAGGAATCGCTAGTAATCGCCGGTCAGCCATA
CGGCGGTGAATCCGTTCCCGGGCCTTGTACACACCGCCCGTCACACTATAGGAGC
TGGCCATGTTTGAAGTCATTACCCTTAACCGTAAGGAGGGGGATGCCTAAGGCTA
GGCTTGCGACTGGAGTGAAGTCGTAACAAGGTAGCCGTACTGGAAGGTGCGGCT
GGATCACCTCCTTT

>13894:emu_db:49273 ["13894:ncbi:49273 ['NC_022417.100817.102307 Cocos nucifera 16S ribosomal RNA, chloroplast']"]

TCTCATGGAGAGTTCGATCCTGGCTCAGGATGAACGCTGGCGGCATGCTTAACAC
ATGCAAGTCGGACGGGAAGTGGTGTTTCCAGTGGCGGACGGGTGAGTAACGCGT
AAGAACCTGCCCTTGGGAGGGGAACAACAACTGGAAACGGTTGCTAATACCCCG
TAGGCTGAGGAGCAAAAGGAGGAATCCGCCCGAGGAGGGGCTCGCGTCTGATTA
GCTAGTTGGTGAGGCAATAGCTTACCAAGGCGATGATCAGTAGCTGGTCCGAGA
GGATGATCAGCCACACTGGGACTGAGACACGGCCCAGACTCCTACGGGAGGCAG
CAGTGGGGAATTTTCCGCAATGGGCGAAAGCCTGACGGAGCAATGCCGCGTGGA
GGTAGAAGGCCCACGGGTCGTGAACTTCTTTTCTCGGAGAAGAAGCAATGACGGT
ATCTGAGGAATAAGCATCGGCTAACTCTGTGCCAGCAGCCGCGGTAAGACAGAG
GATGCAAGCGTTATCCGGAATGATTGGGCGTAAAGCGTCTGTAGGTGGCTTTTCA
AGTCCGCCGTCAAATCCCAGGGCTCAACCCTGGACAGGCGGTGGAAACTACCAA
GCTGGAGTACGGTAGGGGCAGAGGGAATTTCCGGTGGAGCGGTGAAATGCGTAG
AGATCGGAAAGAACACCAACGGCGAAAGCACTCTGCTGGGCCGACACTGACACT
GAGAGACGAAAGCTAGGGGAGCAAATGGGATTAGATACCCCAGTAGTCCTAGCC
GTAAACGATGGATACTAGGCGCTGTGCGTATCGACCCGTGCAGTGCTGTAGCTAA
CGCGTTAAGTATCCCGCCTGGGGAGTACGTTCGCAAGAATGAAACTCAAAGGAA
TTGACGGGGGCCCGCACAAGCGGTGGAGCATGTGGTTTAATTCGATGCAAAGCG
AAGAACCTTACCGGGGCTTGACATGCCGCGAATCCTCTTGAAAGAGAGGGGTGC
CTTCGGGAACGCGGACACAGGTGGTGCATGGCTGTCGTCAGCTCGTGCCGTAAGG
TGTTGGGTTAAGTCCCGCAACGAGCGCAACCCTCGTGTTTAGTTGCCACCGTTGA
GTTTGGAACCCTGAACAGACTGCCGGTGATAAGCCGGAGGAAGGTGAGGATGAC
GTCAAGTCATCATGCCCCTTATGCCCTGGGCGACACACGTGCTACAATGGCCGGG
ACAAAGGGTCGCGATCCCGCGAGGGTGAGCTAACTCCAAAAACCCGTCCTCAGTT
CGGATTGCAGGCTGCAACTCGCCTGCATGAAGCCGGAATCGCTAGTAATCGCCGG
TCAGCCATACGGCGGTGAATTCGTTCCCGGGCCTTGTACACACCGCCCGTCACAC
TATGGGAGCTGGCCATGCCCGAAGTCGTTACCTTAACCGCAAGGAGGGGGATGC
CGAAGGCGGGGCTAGTGACTGGAGTGAAGTCGTAACAAGGTAGCCGTACTGGAA
GGTGCGGCTGGATCACCTCCTTT

>13894:emu_db:49274 ["13894:ncbi:49274 ['NC_022417.136379.137869 Cocos nucifera 16S ribosomal RNA, chloroplast']"]

TCTCATGGAGAGTTCGATCCTGGCTCAGGATGAACGCTGGCGGCATGCTTAACAC
ATGCAAGTCGGACGGGAAGTGGTGTTTCCAGTGGCGGACGGGTGAGTAACGCGT
AAGAACCTGCCCTTGGGAGGGGAACAACAACTGGAAACGGTTGCTAATACCCCG
TAGGCTGAGGAGCAAAAGGAGGAATCCGCCCGAGGAGGGGCTCGCGTCTGATTA
GCTAGTTGGTGAGGCAATAGCTTACCAAGGCGATGATCAGTAGCTGGTCCGAGA
GGATGATCAGCCACACTGGGACTGAGACACGGCCCAGACTCCTACGGGAGGCAG
CAGTGGGGAATTTTCCGCAATGGGCGAAAGCCTGACGGAGCAATGCCGCGTGGA
GGTAGAAGGCCCACGGGTCGTGAACTTCTTTTCTCGGAGAAGAAGCAATGACGGT
ATCTGAGGAATAAGCATCGGCTAACTCTGTGCCAGCAGCCGCGGTAAGACAGAG
GATGCAAGCGTTATCCGGAATGATTGGGCGTAAAGCGTCTGTAGGTGGCTTTTCA
AGTCCGCCGTCAAATCCCAGGGCTCAACCCTGGACAGGCGGTGGAAACTACCAA
GCTGGAGTACGGTAGGGGCAGAGGGAATTTCCGGTGGAGCGGTGAAATGCGTAG
AGATCGGAAAGAACACCAACGGCGAAAGCACTCTGCTGGGCCGACACTGACACT
GAGAGACGAAAGCTAGGGGAGCAAATGGGATTAGATACCCCAGTAGTCCTAGCC
GTAAACGATGGATACTAGGCGCTGTGCGTATCGACCCGTGCAGTGCTGTAGCTAA
CGCGTTAAGTATCCCGCCTGGGGAGTACGTTCGCAAGAATGAAACTCAAAGGAA

TTGACGGGGGCCCGCACAAGCGGTGGAGCATGTGGTTTAATTCGATGCAAAGCG
AAGAACCTTACCGGGGCTTGACATGCCGCGAATCCTCTTGAAAGAGAGGGGTGC
CTTCGGGAACGCGGACACAGGTGGTGCATGGCTGTCGTCAGCTCGTGCCGTAAGG
TGTTGGGTTAAGTCCCGCAACGAGCGCAACCCTCGTGTTTAGTTGCCACCGTTGA
GTTTGGAACCCTGAACAGACTGCCGGTGATAAGCCGGAGGAAGGTGAGGATGAC
GTCAAGTCATCATGCCCCTTATGCCCTGGGCGACACACGTGCTACAATGGCCGGG
ACAAAGGGTCGCGATCCCGCGAGGGTGAGCTAACTCCAAAAACCCGTCCTCAGTT
CGGATTGCAGGCTGCAACTCGCCTGCATGAAGCCGGAATCGCTAGTAATCGCCGG
TCAGCCATACGGCGGTGAATTCGTTCCCGGGCCTTGTACACACCGCCCGTCACAC
TATGGGAGCTGGCCATGCCCGAAGTCGTTACCTTAACCGCAAGGAGGGGGATGC
CGAAGGCGGGGCTAGTGACTGGAGTGAAGTCGTAACAAGGTAGCCGTACTGGAA
GGTGCGGCTGGATCACCTCCTTT

>13894:emu_db:49275 ["13894:ncbi:49275 ['KF285453.100817.102307_U Cocos nucifera 16S ribosomal RNA, chloroplast']"]

TCTCATGGAGAGTTCGATCCTGGCTCAGGATGAACGCTGGCGGCATGCTTAACAC
ATGCAAGTCGGACGGGAAGTGGTGTTTCCAGTGGCGGACGGGTGAGTAACGCGT
AAGAACCTGCCCTTGGGAGGGGAACAACAACTGGAAACGGTTGCTAATACCCCG
TAGGCTGAGGAGCAAAAGGAGGAATCCGCCCGAGGAGGGGCTCGCGTCTGATTA
GCTAGTTGGTGAGGCAATAGCTTACCAAGGCGATGATCAGTAGCTGGTCCGAGA
GGATGATCAGCCACACTGGGACTGAGACACGGCCCAGACTCCTACGGGAGGCAG
CAGTGGGGAATTTTCCGCAATGGGCGAAAGCCTGACGGAGCAATGCCGCGTGGA
GGTAGAAGGCCCACGGGTCGTGAACTTCTTTTCTCGGAGAAGAAGCAATGACGGT
ATCTGAGGAATAAGCATCGGCTAACTCTGTGCCAGCAGCCGCGGTAAGACAGAG
GATGCAAGCGTTATCCGGAATGATTGGGCGTAAAGCGTCTGTAGGTGGCTTTTCA
AGTCCGCCGTCAAATCCCAGGGCTCAACCCTGGACAGGCGGTGGAAACTACCAA
GCTGGAGTACGGTAGGGGCAGAGGGAATTTCCGGTGGAGCGGTGAAATGCGTAG
AGATCGGAAAGAACACCAACGGCGAAAGCACTCTGCTGGGCCGACACTGACACT
GAGAGACGAAAGCTAGGGGAGCAAATGGGATTAGATACCCCAGTAGTCCTAGCC
GTAAACGATGGATACTAGGCGCTGTGCGTATCGACCCGTGCAGTGCTGTAGCTAA
CGCGTTAAGTATCCCGCCTGGGGAGTACGTTCGCAAGAATGAAACTCAAAGGAA
TTGACGGGGGCCCGCACAAGCGGTGGAGCATGTGGTTTAATTCGATGCAAAGCG
AAGAACCTTACCGGGGCTTGACATGCCGCGAATCCTCTTGAAAGAGAGGGGTGC
CTTCGGGAACGCGGACACAGGTGGTGCATGGCTGTCGTCAGCTCGTGCCGTAAGG
TGTTGGGTTAAGTCCCGCAACGAGCGCAACCCTCGTGTTTAGTTGCCACCGTTGA
GTTTGGAACCCTGAACAGACTGCCGGTGATAAGCCGGAGGAAGGTGAGGATGAC
GTCAAGTCATCATGCCCCTTATGCCCTGGGCGACACACGTGCTACAATGGCCGGG
ACAAAGGGTCGCGATCCCGCGAGGGTGAGCTAACTCCAAAAACCCGTCCTCAGTT
CGGATTGCAGGCTGCAACTCGCCTGCATGAAGCCGGAATCGCTAGTAATCGCCGG
TCAGCCATACGGCGGTGAATTCGTTCCCGGGCCTTGTACACACCGCCCGTCACAC
TATGGGAGCTGGCCATGCCCGAAGTCGTTACCTTAACCGCAAGGAGGGGGATGC
CGAAGGCGGGGCTAGTGACTGGAGTGAAGTCGTAACAAGGTAGCCGTACTGGAA
GGTGCGGCTGGATCACCTCCTTT

>13894:emu_db:49276 ["13894:ncbi:49276 ['EU717157.1.1351_U Cocos nucifera 16S ribosomal RNA, chloroplast']"]

AAGAGTTTGATCCTGGCTCAGGATTAACGCTGGCGGCATGCTTAACACATGCAAG
TCGGACGGGAAGTGGTGTTTCCAGTGGCGGACGGGTGAGTAACGCGTAAGAACC
TGCCCTTGGGAGGGGAACAACAACTGGAAACGGTTGCTAATACCCCGTAGGCTG

AGGAGCAAAAGGAGGAATCCGCCCGAGGAGGGGCTCGCGTCTGATTAGCTAGTT
GGTGAGGCAATAGCTTACCAAGGCGATGATCAGTAGCTGGTCCGAGAGGATGAT
CAGCCACACTGGGACTGAGACACGGCCCAGACTCCTACGGGAGGCAGCAGTGGG
GAATTTTCCGCAATGGGCGAAAGCCTGACGGAGCAATGCCGCGTGGAGGTAGAA
GGCCCACGGGTCGTGAACTTCTTTTCTCGGAGAAGAAGCAATGACGGTATCTGAG
GAATAAGCATCGGCTAACTCTGTGCCAGCAGCCGCGGTAAGACAGAGGATGCAA
GCGTTATCCGGAATGATTGGGCGTAAAGCGTCTGTAGGTGGCTTTTCAAGTCCGC
CGTCAAATCCCAGGGCTCAACCCTGGACAGGCGGTGGAAACTACCAAGCTGGAG
TACGGTAGGGGCAGAGGGAATTTCCGGTGGAGCGGTGAAATGCGTAGAGATCGG
AAAGAACACCAACGGCGAAAGCACTCTGCTGGGCCGACACTGACACTGAGAGAC
GAAAGCTAGGGGAGCAAATGGGATTAGATACCCCAGTAGTCCTAGCCGTAAACG
ATGGATACTAGGCGCTGTGCGTATCGACCCGTGCAGTGCTGTAGCTAACGCGTTA
AGTATCCCGCCTGGGGAGTACGTTCGCAAGAATGAAACTCAAAGGAATTGACGG
GGGCCCGCACAAGCGGTGGAGCATGTGGTTTAATTCGATGCAAAGCGAAGAACC
TTACCGGGGCTTGACATGCCGCGAATCCTCTTGAAAGAGAGGGGTGCCTTCGGGA
ACGCGGACACAGGTGGTGCATGGCTGTCGTCAGCTCGTGCCGTAAGGTGTTGGGT
TAAGTCCCGCAACGAGCGCAACCCTCGTGTTTAGTTGCCACCGTTGAGTTTGGAA
CCCTGAACAGACTGCCGGTGATAAGCCGGAGGAAGGTGAGGATGACGTCAAGTC
ATCATGCCCCTTATGCCCTGGGCGACACACGTGCTACAATGGCCGGGACAAAGGG
TCGCGATCCCGCGAGGGTGAGCTAACTCCAAAAACCCGTCCTCAGTTCGGATTGC
AGGCTGCAACTCGCCTGCATGAAGCCGGAATCGCTAGTAATCGCCGGTCAGCCAT
ACGGCGGTGAATTCGTTCACGGGGTTTGTACACACGGCCCGTCAA

>3708:emu_db:49277 ["3708:ncbi:49277 ['NC_016734.99831.101306 Brassica napus 16S ribosomal RNA, chloroplast']"]

TCTCATGGAGAGTTCGATCCTGGCTCAGGATGAACGCTGGCGGCATGCTTAACAC
ATGCAAGTCGGACGGGAAGTGGTGTTTCCAGTGGCGGACGGGTGAGTAACGCGT
AAGAACCTGCCCTTGGGAGGGGAACAACAGCTGGAAACGGCTGCTAATACCCCG
TAGGCTGAGGAGCAAAAGGAGGAATCCGCCCGAGGAGGGGCTCGCGTCTGATTA
GCTAGTTGGTGAGGCAATAGCTTACCAAGGCGATGATCAGTAGCTGGTCCGAGA
GGATGATCAGCCACACTGGGACTGAGACACGGCCCAGACTCCTACGGGAGGCAG
CAGTGGGGAATTTTCCGCAATGGGCGAAAGCCTGACGGAGCAATGCCGCGTGGA
GGTAGAAGGCCTACGGGTCCTGAACTTCTTTTCCCAGAGAAGAAGCAATGACGGT
ATCTGGGGAATAAGCATCGGCTAACTCTGTGCCAGCAGCCGCGGTAATACAGAG
GATGCAAGCGTTATCCGGAATGATTGGGCGTAAAGCGTCTGTAGGTGGCTTTTTA
AGTCCGCCGTCAAATCCCAGGGCTCAACCCTGGACAGGCGGTGGAAACTACCAA
GCTTGAGTACGGTAGGGGCAGAGGGAATTTCCGGTGGAGCGGTGAAATGCGTAG
AGATCGGAAAGAACACCAACGGCGAAAGCACTCTGCTGGGCCGACACTGACACT
GAGAGACGAAAGCTAGGGGAGCGAATGGGATTAGATACCCCAGTAGTCCTAGCC
GTAAACGATGGATACTAGGCGCTGTGCGTATCGACCCGTGCAGTGCTGTAGCTAA
CGCGTTAAGTATCCCGCCTGGGGAGTACGTTCGCAAGAATGAAACTCAAAGGAA
TTGACGGGGGCCCGCACAAGCGGTGGAGGATGCAAAGCGAAGCACCTTACCAGG
GCTTGACATGCCGCGAATCCTCTTGAAAGAGAGGGGTGCCTTCGGGAACGCGGA
CACAGGTGGTGCATGGCTGTCGTCAGCTCGTGCCGTAAGGTGTTGGGTTAAGTCC
CGCAACGAGCGCAACCCTCGTGTTTAGTTGCCACCGTTGAGTTTGGAACCCTGAA
CAGACTGCCGGTGATAAGCCGGAGGAAGGTGAGGATGACGTCAAGTCATCATGC
CCCTTATGCCCTGGGCGACACACGTGCTACAATGGCCGGGACAAAGGGTCGCGAT
CCCGCGAGGGTGAGCTAACTCCAAAAACCCGTCCTCAGTTCGGATTGCAGGCTGC
AACTCGCCTGCATGAAGCCGGAATCGCTAGTAATCGCCGGTCAGCCATACGGCGG
TGAATTCGTTCCCGGGCCTTGTACACACCGCCCGTCACACTATGGGAGCTGGCCA

TGCCCGAAGTCGTTACCTTAACCGCAAGGAGGGGGGTGCCGAAGGCAGGGCTAG
TGACTGGAGTGAAGTCGTAACAAGGTAGCCGTACTGGAAGGTGCGGCTGGATCA
CCTCCTTT

>3708:emu_db:49278 ["3708:ncbi:49278 ['NC_016734.134585.136060 Brassica napus 16S ribosomal RNA, chloroplast']"]

TCTCATGGAGAGTTCGATCCTGGCTCAGGATGAACGCTGGCGGCATGCTTAACAC
ATGCAAGTCGGACGGGAAGTGGTGTTTCCAGTGGCGGACGGGTGAGTAACGCGT
AAGAACCTGCCCTTGGGAGGGGAACAACAGCTGGAAACGGCTGCTAATACCCCG
TAGGCTGAGGAGCAAAAGGAGGAATCCGCCCGAGGAGGGGCTCGCGTCTGATTA
GCTAGTTGGTGAGGCAATAGCTTACCAAGGCGATGATCAGTAGCTGGTCCGAGA
GGATGATCAGCCACACTGGGACTGAGACACGGCCCAGACTCCTACGGGAGGCAG
CAGTGGGGAATTTTCCGCAATGGGCGAAAGCCTGACGGAGCAATGCCGCGTGGA
GGTAGAAGGCCTACGGGTCCTGAACTTCTTTTCCCAGAGAAGAAGCAATGACGGT
ATCTGGGGAATAAGCATCGGCTAACTCTGTGCCAGCAGCCGCGGTAATACAGAG
GATGCAAGCGTTATCCGGAATGATTGGGCGTAAAGCGTCTGTAGGTGGCTTTTTA
AGTCCGCCGTCAAATCCCAGGGCTCAACCCTGGACAGGCGGTGGAAACTACCAA
GCTTGAGTACGGTAGGGGCAGAGGGAATTTCCGGTGGAGCGGTGAAATGCGTAG
AGATCGGAAAGAACACCAACGGCGAAAGCACTCTGCTGGGCCGACACTGACACT
GAGAGACGAAAGCTAGGGGAGCGAATGGGATTAGATACCCCAGTAGTCCTAGCC
GTAAACGATGGATACTAGGCGCTGTGCGTATCGACCCGTGCAGTGCTGTAGCTAA
CGCGTTAAGTATCCCGCCTGGGGAGTACGTTCGCAAGAATGAAACTCAAAGGAA
TTGACGGGGGCCCGCACAAGCGGTGGAGGATGCAAAGCGAAGCACCTTACCAGG
GCTTGACATGCCGCGAATCCTCTTGAAAGAGAGGGGTGCCTTCGGGAACGCGGA
CACAGGTGGTGCATGGCTGTCGTCAGCTCGTGCCGTAAGGTGTTGGGTTAAGTCC
CGCAACGAGCGCAACCCTCGTGTTTAGTTGCCACCGTTGAGTTTGGAACCCTGAA
CAGACTGCCGGTGATAAGCCGGAGGAAGGTGAGGATGACGTCAAGTCATCATGC
CCCTTATGCCCTGGGCGACACACGTGCTACAATGGCCGGGACAAAGGGTCGCGAT
CCCGCGAGGGTGAGCTAACTCCAAAAACCCGTCCTCAGTTCGGATTGCAGGCTGC
AACTCGCCTGCATGAAGCCGGAATCGCTAGTAATCGCCGGTCAGCCATACGGCGG
TGAATTCGTTCCCGGGCCTTGTACACACCGCCCGTCACACTATGGGAGCTGGCCA
TGCCCGAAGTCGTTACCTTAACCGCAAGGAGGGGGGTGCCGAAGGCAGGGCTAG
TGACTGGAGTGAAGTCGTAACAAGGTAGCCGTACTGGAAGGTGCGGCTGGATCA
CCTCCTTT

>3708:emu_db:49279 ["3708:ncbi:49279 ['GQ861354.99831.101306_U Brassica napus 16S ribosomal RNA, chloroplast']"]

TCTCATGGAGAGTTCGATCCTGGCTCAGGATGAACGCTGGCGGCATGCTTAACAC
ATGCAAGTCGGACGGGAAGTGGTGTTTCCAGTGGCGGACGGGTGAGTAACGCGT
AAGAACCTGCCCTTGGGAGGGGAACAACAGCTGGAAACGGCTGCTAATACCCCG
TAGGCTGAGGAGCAAAAGGAGGAATCCGCCCGAGGAGGGGCTCGCGTCTGATTA
GCTAGTTGGTGAGGCAATAGCTTACCAAGGCGATGATCAGTAGCTGGTCCGAGA
GGATGATCAGCCACACTGGGACTGAGACACGGCCCAGACTCCTACGGGAGGCAG
CAGTGGGGAATTTTCCGCAATGGGCGAAAGCCTGACGGAGCAATGCCGCGTGGA
GGTAGAAGGCCTACGGGTCCTGAACTTCTTTTCCCAGAGAAGAAGCAATGACGGT
ATCTGGGGAATAAGCATCGGCTAACTCTGTGCCAGCAGCCGCGGTAATACAGAG
GATGCAAGCGTTATCCGGAATGATTGGGCGTAAAGCGTCTGTAGGTGGCTTTTTA
AGTCCGCCGTCAAATCCCAGGGCTCAACCCTGGACAGGCGGTGGAAACTACCAA
GCTTGAGTACGGTAGGGGCAGAGGGAATTTCCGGTGGAGCGGTGAAATGCGTAG

AGATCGGAAAGAACACCAACGGCGAAAGCACTCTGCTGGGCCGACACTGACACT
GAGAGACGAAAGCTAGGGGAGCGAATGGGATTAGATACCCCAGTAGTCCTAGCC
GTAAACGATGGATACTAGGCGCTGTGCGTATCGACCCGTGCAGTGCTGTAGCTAA
CGCGTTAAGTATCCCGCCTGGGGAGTACGTTCGCAAGAATGAAACTCAAAGGAA
TTGACGGGGGCCCGCACAAGCGGTGGAGGATGCAAAGCGAAGCACCTTACCAGG
GCTTGACATGCCGCGAATCCTCTTGAAAGAGAGGGGTGCCTTCGGGAACGCGGA
CACAGGTGGTGCATGGCTGTCGTCAGCTCGTGCCGTAAGGTGTTGGGTTAAGTCC
CGCAACGAGCGCAACCCTCGTGTTTAGTTGCCACCGTTGAGTTTGGAACCCTGAA
CAGACTGCCGGTGATAAGCCGGAGGAAGGTGAGGATGACGTCAAGTCATCATGC
CCCTTATGCCCTGGGCGACACACGTGCTACAATGGCCGGGACAAAGGGTCGCGAT
CCCGCGAGGGTGAGCTAACTCCAAAAACCCGTCCTCAGTTCGGATTGCAGGCTGC
AACTCGCCTGCATGAAGCCGGAATCGCTAGTAATCGCCGGTCAGCCATACGGCGG
TGAATTCGTTCCCGGGCCTTGTACACACCGCCCGTCACACTATGGGAGCTGGCCA
TGCCCGAAGTCGTTACCTTAACCGCAAGGAGGGGGGTGCCGAAGGCAGGGCTAG
TGACTGGAGTGAAGTCGTAACAAGGTAGCCGTACTGGAAGGTGCGGCTGGATCA
CCTCCTTT

>3708:emu_db:49280 ["3708:ncbi:49280 ['GQ861354.134585.136060_U Brassica napus 16S ribosomal RNA, chloroplast']"]

TCTCATGGAGAGTTCGATCCTGGCTCAGGATGAACGCTGGCGGCATGCTTAACAC
ATGCAAGTCGGACGGGAAGTGGTGTTTCCAGTGGCGGACGGGTGAGTAACGCGT
AAGAACCTGCCCTTGGGAGGGGAACAACAGCTGGAAACGGCTGCTAATACCCCG
TAGGCTGAGGAGCAAAAGGAGGAATCCGCCCGAGGAGGGGCTCGCGTCTGATTA
GCTAGTTGGTGAGGCAATAGCTTACCAAGGCGATGATCAGTAGCTGGTCCGAGA
GGATGATCAGCCACACTGGGACTGAGACACGGCCCAGACTCCTACGGGAGGCAG
CAGTGGGGAATTTTCCGCAATGGGCGAAAGCCTGACGGAGCAATGCCGCGTGGA
GGTAGAAGGCCTACGGGTCCTGAACTTCTTTTCCCAGAGAAGAAGCAATGACGGT
ATCTGGGGAATAAGCATCGGCTAACTCTGTGCCAGCAGCCGCGGTAATACAGAG
GATGCAAGCGTTATCCGGAATGATTGGGCGTAAAGCGTCTGTAGGTGGCTTTTTA
AGTCCGCCGTCAAATCCCAGGGCTCAACCCTGGACAGGCGGTGGAAACTACCAA
GCTTGAGTACGGTAGGGGCAGAGGGAATTTCCGGTGGAGCGGTGAAATGCGTAG
AGATCGGAAAGAACACCAACGGCGAAAGCACTCTGCTGGGCCGACACTGACACT
GAGAGACGAAAGCTAGGGGAGCGAATGGGATTAGATACCCCAGTAGTCCTAGCC
GTAAACGATGGATACTAGGCGCTGTGCGTATCGACCCGTGCAGTGCTGTAGCTAA
CGCGTTAAGTATCCCGCCTGGGGAGTACGTTCGCAAGAATGAAACTCAAAGGAA
TTGACGGGGGCCCGCACAAGCGGTGGAGGATGCAAAGCGAAGCACCTTACCAGG
GCTTGACATGCCGCGAATCCTCTTGAAAGAGAGGGGTGCCTTCGGGAACGCGGA
CACAGGTGGTGCATGGCTGTCGTCAGCTCGTGCCGTAAGGTGTTGGGTTAAGTCC
CGCAACGAGCGCAACCCTCGTGTTTAGTTGCCACCGTTGAGTTTGGAACCCTGAA
CAGACTGCCGGTGATAAGCCGGAGGAAGGTGAGGATGACGTCAAGTCATCATGC
CCCTTATGCCCTGGGCGACACACGTGCTACAATGGCCGGGACAAAGGGTCGCGAT
CCCGCGAGGGTGAGCTAACTCCAAAAACCCGTCCTCAGTTCGGATTGCAGGCTGC
AACTCGCCTGCATGAAGCCGGAATCGCTAGTAATCGCCGGTCAGCCATACGGCGG
TGAATTCGTTCCCGGGCCTTGTACACACCGCCCGTCACACTATGGGAGCTGGCCA
TGCCCGAAGTCGTTACCTTAACCGCAAGGAGGGGGGTGCCGAAGGCAGGGCTAG
TGACTGGAGTGAAGTCGTAACAAGGTAGCCGTACTGGAAGGTGCGGCTGGATCA
CCTCCTTT

>4679:emu_db:49281 ["4679:ncbi:49281 ['GAAO01015831.123.1440 Allium cepa 16S ribosomal RNA, chloroplast']"]

AGAGTTCGATCCTGGCTCAGGATGAACGCTAGCGGCATGCTTAACACATGCAAGT
CGGACGGGAAGTGGTGTTTCCAGTGGCGGACGGGTGAGTAACGCGTAAGAACCT
GCCCTTGGGAGGGGAACAACAACTGGAAACGGTTGCTAATACCCCGTAGGCTGA
GGAGCAAAAGGAGGAATCCGCCCGAGGAGGGGCTCGCGTCTGATTAGCTAGTTG
GTGAGGCAATAGCTTACCAAGGCGATGATCAGTAGCTGGTCCGAGAGGATGATC
AGCCACACTGGGACTGAGACACGGCCCAGACTCCTACGGGAGGCAGCAGTGGGG
AATTTTCCGCAATGGGCGAAAGCCTGACGGAGCAATGCCGCGTGGAGGTAGAAG
GCCCACGGGTCATGAACTTCTTTTCTCAGAGAAGAAGCAATGACGGTATCTGAGG
AATAAGCATCGGCTAACTCTGTGCCAGCAGCCGCGGTAAGACAGAGGATGCAAG
CGTTATCCGGAATGATTGGGCGTAAAGCGTCTGTAGGTGGCTTTTCAAGTCCGCC
GTCAAATCCCAGGGCTCAACCCTGGACAGGCGGTGGAAACTACCAAGCTGGAGT
ACGGTAGGGGCAGAGGGAATTTCCGGTGGAGCGGTGAAATGCGTAGAGATCGGG
AAGAACACCAACGGCGAAAGCACTCTGCTGGGCCGACACTGACACTGAGAGACG
AAAGCTAGGGGAGCAAATGGGATTAGATACCCCAGTAGTCCTAGCCGTAAACGA
TGGATACTAGGCGCTGCGCGTATCGACCCGTGCAGTGCTGTAGCTAACGCGTTAA
GTATCCCGCCTGGGGAGTACGTTCGCAAGAATGAAACTCAAAGGAATTGACGGG
GGCCCGCACAAGCGGTGGAGCATGTGGTTTAATTCGATCAAAGCGAAGAACCTT
ACCAGGGCTTGACATGCCGCGAATCCTCTTGAAAGAGAGGGGTGCCTTCGGGAA
CGCGGACACAGGTGGTGCATGGCTGTCGTCAGCTCGTGCCGTAAGGTGTTGGGTT
AAGTCTCGCAACGAGCGCAACCCTCGTGTTTAGTTGCCACCATTGAGTGTGGAAC
CCTGAACAGACTGCCGGTGATAAGCCGGAGGAAGGTGAGGATGACGTCAAGTCA
TCATGCCCCTTATGCCCTGGGCGACACACGTGCTACAATGGGCGGGACAAAGGGT
CGCGATCCCGCGAGGGTGAGCTAACTCCAAAAACCCGTCCTCAGTTCGGATTGCA
GGCTGCAACTCGCCTGCATGAAGCCGGAATCGCTAGTAATCGCCGGTCAGCCATA
CGACGGTGAATTC

>3711:emu_db:49282 ["3711:ncbi:49282 ['AC234765.100272.101741_U Brassica rapa 16S ribosomal RNA, chloroplast']"]

AGAGTTCGATCCTGGCTCAGGATGAACGCTGGCGGCATGCTTAACACATGCAAGT
CGGACGGGAAGTGGTGTTTCCAGTGGCGGACGGGTGAGTAACGCGTAAGAACCT
GCCCTTGGGAGGGGAACAACAGCTGGAAACGGCTGCTAATACCCCGTAGGCTGA
GGAGCAAAAGGAGGAATCCGCCCGAGGAGGGGCTCGCGTCTGATTAGCTAGTTG
GTGAGGCAATAGCTTACCAAGGCGATGATCAGTAGCTGGTCCGAGAGGATGATC
AGCCACACTGGGACTGAGACACGGCCCAGACTCCTACGGGAGGCAGCAGTGGGG
AATTTTCCGCAATGGGCGAAAGCCTGACGGAGCAATGCCGCGTGGAGGTAGAAG
GCCTACGGGTCCTGAACTTCTTTTCCCAGAGAAGAAGCAATGACGGTATCTGGGG
AATAAGCATCGGCTAACTCTGTGCCAGCAGCCGCGGTAATACAGAGGATGCAAG
CGTTATCCGGAATGATTGGGCGTAAAGCGTCTGTAGGTGGCTTTTTAAGTCCGCC
GTCAAATCCCAGGGCTCAACCCTGGACAGGCGGTGGAAACTACCAAGCTTGAGT
ACGGTAGGGGCAGAGGGAATTTCCGGTGGAGCGGTGAAATGCGTAGAGATCGGA
AAGAACACCAACGGCGAAAGCACTCTGCTGGGCCGACACTGACACTGAGAGACG
AAAGCTAGGGGAGCGAATGGGATTAGATACCCCAGTAGTCCTAGCCGTAAACGA
TGGATACTAGGCGCTGTGCGTATCGACCCGTGCAGTGCTGTAGCTAACGCGTTAA
GTATCCCGCCTGGGGAGTACGTTCGCAAGAATGAAACTCAAAGGAATTGACGGG
GGCCCGCACAAGCGGTGGAGCATGTGGTTTAATTCGATGCAAAGCGAAGAACCT
TACCAGGGCTTGACATGCCGCGAATCCTCTTGAAAGAGAGGGGTGCCTTCGGGAA
CGCGGACACAGGTGGTGCATGGCTGTCGTCAGCTCGTGCCGTAAGGTGTTGGGTT
AAGTCCCGCAACGAGCGCAACCCTCGTGTTTAGTTGCCACCGTTGAGTTTGGAAC
CCTGAACAGACTGCCGGTGATAAGCCGGAGGAAGGTGAGGATGACGTCAAGTCA
TCATGCCCCTTATGCCCTGGGCGACACACGTGCTACAATGGCCGGGACAAAGGGT

CGCGATCCCGCGAGGGTGAGCTAACTCCAAAAACCCGTCCTCAGTTCGGATTGCA
GGCTGCAACTCGCCTGCATGAAGCCGGAATCGCTAGTAATCGCCGGTCAGCCATA
CGGCGGTGAATTCGTTCCCGGGCCTTGTACACACCGCCCGTCACACTATGGGAGC
TGGCCATGCCCGAAGTCGTTACCTTAACCGCAAGGAGGGGGGTGCCGAAGGCAG
GGCTAGTGACTGGAGTGAAGTCGTAACAAGGTAGCCGTACTGGAAGGTGCGGCT
G

>3711:emu_db:49283 ["3711:ncbi:49283 ['DQ231548.135204.136673_U Brassica rapa 16S ribosomal RNA, chloroplast']"]

AGAGTTCGATCCTGGCTCAGGATGAACGCTGGCGGCATGCTTAACACATGCAAGT
CGGACGGGAAGTGGTGTTTCCAGTGGCGGACGGGTGAGTAACGCGTAAGAACCT
GCCCTTGGGAGGGGAACAACAGCTGGAAACGGCTGCTAATACCCCGTAGGCTGA
GGAGCAAAAGGAGGAATCCGCCCGAGGAGGGGCTCGCGTCTGATTAGCTAGTTG
GTGAGGCAATAGCTTACCAAGGCGATGATCAGTAGCTGGTCCGAGAGGATGATC
AGCCACACTGGGACTGAGACACGGCCCAGACTCCTACGGGAGGCAGCAGTGGGG
AATTTTCCGCAATGGGCGAAAGCCTGACGGAGCAATGCCGCGTGGAGGTAGAAG
GCCTACGGGTCCTGAACTTCTTTTCCCAGAGAAGAAGCAATGACGGTATCTGGGG
AATAAGCATCGGCTAACTCTGTGCCAGCAGCCGCGGTAATACAGAGGATGCAAG
CGTTATCCGGAATGATTGGGCGTAAAGCGTCTGTAGGTGGCTTTTTAAGTCCGCC
GTCAAATCCCAGGGCTCAACCCTGGACAGGCGGTGGAAACTACCAAGCTTGAGT
ACGGTAGGGGCAGAGGGAATTTCCGGTGGAGCGGTGAAATGCGTAGAGATCGGA
AAGAACACCAACGGCGAAAGCACTCTGCTGGGCCGACACTGACACTGAGAGACG
AAAGCTAGGGGAGCGAATGGGATTAGATACCCCAGTAGTCCTAGCCGTAAACGA
TGGATACTAGGCGCTGTGCGTATCGACCCGTGCAGTGCTGTAGCTAACGCGTTAA
GTATCCCGCCTGGGGAGTACGTTCGCAAGAATGAAACTCAAAGGAATTGACGGG
GGCCCGCACAAGCGGTGGAGCATGTGGTTTAATTCGATGCAAAGCGAAGAACCT
TACCAGGGCTTGACATGCCGCGAATCCTCTTGAAAGAGAGGGGTGCCTTCGGGAA
CGCGGACACAGGTGGTGCATGGCTGTCGTCAGCTCGTGCCGTAAGGTGTTGGGTT
AAGTCCCGCAACGAGCGCAACCCTCGTGTTTAGTTGCCACCGTTGAGTTTGGAAC
CCTGAACAGACTGCCGGTGATAAGCCGGAGGAAGGTGAGGATGACGTCAAGTCA
TCATGCCCCTTATGCCCTGGGCGACACACGTGCTACAATGGCCGGGACAAAGGGT
CGCGATCCCGCGAGGGTGAGCTAACTCCAAAAACCCGTCCTCAGTTCGGATTGCA
GGCTGCAACTCGCCTGCATGAAGCCGGAATCGCTAGTAATCGCCGGTCAGCCATA
CGGCGGTGAATTCGTTCCCGGGCCTTGTACACACCGCCCGTCACACTATGGGAGC
TGGCCATGCCCGAAGTCGTTACCTTAACCGCAAGGAGGGGGGTGCCGAAGGCAG
GGCTAGTGACTGGAGTGAAGTCGTAACAAGGTAGCCGTACTGGAAGGTGCGGCT
G

>3711:emu_db:49284 ["3711:ncbi:49284 ['AC189190.93110.94579_U Brassica rapa 16S ribosomal RNA, chloroplast']"]

AGAGTTCGATCCTGGCTCAGGATGAACGCTGGCGGCATGCTTAACACATGCAAGT
CGGACGGGAAGTGGTGTTTCCAGTGGCGGACGGGTGAGTAACGCGTAAGAACCT
GCCCTTGGGAGGGGAACAACAGCTGGAAACGGCTGCTAATACCCCGTAGGCTGA
GGAGCAAAAGGAGGAATCCGCCCGAGGAGGGGCTCGCGTCTGATTAGCTAGTTG
GTGAGGCAATAGCTTACCAAGGCGATGATCAGTAGCTGGTCCGAGAGGATGATC
AGCCACACTGGGACTGAGACACGGCCCAGACTCCTACGGGAGGCAGCAGTGGGG
AATTTTCCGCAATGGGCGAAAGCCTGACGGAGCAATGCCGCGTGGAGGTAGAAG
GCCTACGGGTCCTGAACTTCTTTTCCCAGAGAAGAAGCAATGACGGTATCTGGGG
AATAAGCATCGGCTAACTCTGTGCCAGCAGCCGCGGTAATACAGAGGATGCAAG

CGTTATCCGGAATGATTGGGCGTAAAGCGTCTGTAGGTGGCTTTTTAAGTCCGCC
GTCAAATCCCAGGGCTCAACCCTGGACAGGCGGTGGAAACTACCAAGCTTGAGT
ACGGTAGGGGCAGAGGGAATTTCCGGTGGAGCGGTGAAATGCGTAGAGATCGGA
AAGAACACCAACGGCGAAAGCACTCTGCTGGGCCGACACTGACACTGAGAGACG
AAAGCTAGGGGAGCGAATGGGATTAGATACCCCAGTAGTCCTAGCCGTAAACGA
TGGATACTAGGCGCTGTGCGTATCGACCCGTGCAGTGCTGTAGCTAACGCGTTAA
GTATCCCGCCTGGGGAGTACGTTCGCAAGAATGAAACTCAAAGGAATTGACGGG
GGCCCGCACAAGCGGTGGAGCATGTGGTTTAATTCGATGCAAAGCGAAGAACCT
TACCAGGGCTTGACATGCCGCGAATCCTCTTGAAAGAGAGGGGTGCCTTCGGGAA
CGCGGACACAGGTGGTGCATGGCTGTCGTCAGCTCGTGCCGTAAGGTGTTGGGTT
AAGTCCCGCAACGAGCGCAACCCTCGTGTTTAGTTGCCACCGTTGAGTTTGGAAC
CCTGAACAGACTGCCGGTGATAAGCCGGAGGAAGGTGAGGATGACGTCAAGTCA
TCATGCCCCTTATGCCCTGGGCGACACACGTGCTACAATGGCCGGGACAAAGGGT
CGCGATCCCGCGAGGGTGAGCTAACTCCAAAAACCCGTCCTCAGTTCGGATTGCA
GGCTGCAACTCGCCTGCATGAAGCCGGAATCGCTAGTAATCGCCGGTCAGCCATA
CGGCGGTGAATTCGTTCCCGGGCCTTGTACACACCGCCCGTCACACTATGGGAGC
TGGCCATGCCCGAAGTCGTTACCTTAACCGCAAGGAGGGGGGTGCCGAAGGCAG
GGCTAGTGACTGGAGTGAAGTCGTAACAAGGTAGCCGTACTGGAAGGTGCGGCT
G

>3711:emu_db:49285 ["3711:ncbi:49285 ['DQ231548.100091.101560_U Brassica rapa 16S ribosomal RNA, chloroplast']"]

AGAGTTCGATCCTGGCTCAGGATGAACGCTGGCGGCATGCTTAACACATGCAAGT
CGGACGGGAAGTGGTGTTTCCAGTGGCGGACGGGTGAGTAACGCGTAAGAACCT
GCCCTTGGGAGGGGAACAACAGCTGGAAACGGCTGCTAATACCCCGTAGGCTGA
GGAGCAAAAGGAGGAATCCGCCCGAGGAGGGGCTCGCGTCTGATTAGCTAGTTG
GTGAGGCAATAGCTTACCAAGGCGATGATCAGTAGCTGGTCCGAGAGGATGATC
AGCCACACTGGGACTGAGACACGGCCCAGACTCCTACGGGAGGCAGCAGTGGGG
AATTTTCCGCAATGGGCGAAAGCCTGACGGAGCAATGCCGCGTGGAGGTAGAAG
GCCTACGGGTCCTGAACTTCTTTTCCCAGAGAAGAAGCAATGACGGTATCTGGGG
AATAAGCATCGGCTAACTCTGTGCCAGCAGCCGCGGTAATACAGAGGATGCAAG
CGTTATCCGGAATGATTGGGCGTAAAGCGTCTGTAGGTGGCTTTTTAAGTCCGCC
GTCAAATCCCAGGGCTCAACCCTGGACAGGCGGTGGAAACTACCAAGCTTGAGT
ACGGTAGGGGCAGAGGGAATTTCCGGTGGAGCGGTGAAATGCGTAGAGATCGGA
AAGAACACCAACGGCGAAAGCACTCTGCTGGGCCGACACTGACACTGAGAGACG
AAAGCTAGGGGAGCGAATGGGATTAGATACCCCAGTAGTCCTAGCCGTAAACGA
TGGATACTAGGCGCTGTGCGTATCGACCCGTGCAGTGCTGTAGCTAACGCGTTAA
GTATCCCGCCTGGGGAGTACGTTCGCAAGAATGAAACTCAAAGGAATTGACGGG
GGCCCGCACAAGCGGTGGAGCATGTGGTTTAATTCGATGCAAAGCGAAGAACCT
TACCAGGGCTTGACATGCCGCGAATCCTCTTGAAAGAGAGGGGTGCCTTCGGGAA
CGCGGACACAGGTGGTGCATGGCTGTCGTCAGCTCGTGCCGTAAGGTGTTGGGTT
AAGTCCCGCAACGAGCGCAACCCTCGTGTTTAGTTGCCACCGTTGAGTTTGGAAC
CCTGAACAGACTGCCGGTGATAAGCCGGAGGAAGGTGAGGATGACGTCAAGTCA
TCATGCCCCTTATGCCCTGGGCGACACACGTGCTACAATGGCCGGGACAAAGGGT
CGCGATCCCGCGAGGGTGAGCTAACTCCAAAAACCCGTCCTCAGTTCGGATTGCA
GGCTGCAACTCGCCTGCATGAAGCCGGAATCGCTAGTAATCGCCGGTCAGCCATA
CGGCGGTGAATTCGTTCCCGGGCCTTGTACACACCGCCCGTCACACTATGGGAGC
TGGCCATGCCCGAAGTCGTTACCTTAACCGCAAGGAGGGGGGTGCCGAAGGCAG
GGCTAGTGACTGGAGTGAAGTCGTAACAAGGTAGCCGTACTGGAAGGTGCGGCT
G

>4113:emu_db:49286 ["4113:ncbi:49286 ['AC234828.125208.126662_U Solanum tuberosum 16S ribosomal RNA, chloroplast']"]

AGAGTTCGATCCGGGCTCAGGATGAACGCTGGCGGCATGCTTAATACATGCAAGT
CGGACGGGAAACACGAGAAATGGTGTTTCTAGTGGAGGATGGGTGAGTAACGCG
TAAAAACCTGCCCTTGGGAGGGGAACAACAGTTGGAAATGACTGCTAATACCCC
GTAGGCTGAGGAGCAAAAATAGGAATCTGCTCGAGGAGGGGCTCGCGTCTGATT
AGCTAGTTGGTGAGGCAATAGCTTACTAAGGCGATGATCAGTAGCTGGTCTGAGA
GGATGATCAACCACACTGGGACTGAGACACAGCCCAAACTCCTACAGGAGGCAG
TAGTGAGGAATTCTTCGCAATGGGAGAAATCCTGACGGAGCAATACCGCGTAGA
GGTAGAAGGCCCACGGCTTGTGAACTTCTTTTCTCAGAGAAGAAGCAATGCCTGT
ATTTGGGGAATAAGCATCAGCTAACTCTGTACCAGCAACCACAATAATACAAATG
ATGCAAGCATTATCAAGAATAATTGGGTGTAAAGTGTATATAGATGGATTTTTAA
GTCCGTCGTCAAATCCCAGGGCTGAATCCTTGACAGGCGGTGGAAACTACCAAGT
TGGAGTACGGTAGGGGCATAGGGAATTTCTGGTGGAACGGTGAAATGCATAGAA
ATCGAAAAGAACCCCAACAGTGAAAGCACTCTGTTGGGCCGACACCGACAGTGA
GAGACAAAAGCTAGGGGAGTGAATGAGATTAGATACCCAGTAGTCCTAGCCATA
AGCGATGGATACTAAGTGTTGTGCATATCGGCAGTACTGTAGCTAACACGTTAAG
TATCCCGCATGGGGAGTAAGTTCGCAAGAATGAAACTCAAAGAAATTGATGGGG
ACCCACAGAAGCGTTGGAGCATGTGGTTTAATTCAATGCAAGGCGAAGAACCTTA
CCAGGGCTTGACATGCCGCAAATCTATTTATAGAGAGGGGTGCCTTTGAGAATGC
AGACACAGGTGGTGCATGGCTGCCGTCAGCTCGTGTCGTAAGGTGTTGGGTTAAG
TCCCGCAACAAGTATAACCCTCTTGTTTAGTTTCTATCATTGAATTTGGAACCCTG
AACAAACTGCCTGTGATAAGCCAGAGGAAGGTGACTCGGGAATTAATGAAGTCA
CTTCACTTCAATGAAAGGGAGTATGTTTTTCTTGTCAGCAGGATCCTCCCTTCTA
TCTATCAACCATTTCATTATAGAACTATCTCTTTGAGCTATGGAGCTAACAAAAA
ACACTCATCTGCATTCTTAAAGTCTTAATCATGTTCCAGTTCCCAAAACTTATACC
TTTCTTCTGCAATGATTGAATTATGGGGTTCCGCATTTCGAAAAGTCTTTTTGAT
GCATTTGTAATAGCCTATGTGTTCCAAAAAAGAACGTTCTACGCCCGGGCCTCTTT
TCAGTAGATGAGATTAGGATGAAAGAGCTT

>4113:emu_db:49287 ["4113:ncbi:49287 ['AEWC01004521.94.1495_U Solanum tuberosum 16S ribosomal RNA, chloroplast']"]

TGAGCTACAGGCCCCACCCCGTCTCCACTGGATCTCCGAGATCCTTTCGATGACC
CATTTCCAGTGGTGGACGGGTGAGCAACGTGTAAGAACCTGCCTTTGGGAGGGG
AACAACAGCTGGAAACAGCTGCGAATACCCCGTAGGCTGAGGAGCAAAAGGAAG
AATCCACCCGAGGAGGGGATCGCGTCTGATTAGCTAGTTGGTGAGGCAATAGCTT
ACCAAGGCAATGATCAGTAGCTGGTCCGAGAGGATGATCAACCACACTGGGACT
GAGACACAACCCAGACTCCTACGGGAGGCAGCAGTGGGGAATTTTCAGCAATGG
GCGAAAGCCTGACGGAGCAATGCTGTGTGGAGGTAGAAGGCCCACGGGTCGTGA
GCTTCCTTTTTCGGAGAAGAAGTAATGACGGTATCTGGGGAATAAGCATCAACTA
ACTCTGTGCCAGCAGCCGCGGTAATACAGAGGATGCAAGTGTTATTCGGAATGAT
TGGGCGTAAAGCGTCTGTATGTGGCTTTTTAAGTCCGCCATCAAATCCCAGGGAT
CAACACTAGACAGGCGGTGGAAACTACCAAGCTGGAGTATGGTAGGGGCAGAGG
GAATTTTCGGTGGAGCGGTGAAATGCGTAGAGATCGGAAAGAACACCAACGGCG
AAAGCACTCTGCTGGGCCGACACTGACACTGAGAGACGAAAGCTAGGGGAGCGA
ATGGGATTAGATACCCCAGTAGTCCTAGCCGTAAACGATGGATACTAGGCGCTGT
GCGTATTGACCCGTGCAGTGCTGTAGCTAATGCGTTAAGTATCCTGCCTGGGGAG
TACACTCGTAAGAATGAAACTCAAAGGAATTGACGGGAGCCCGCACAAGCGTTG
GAGCATGTGGTTTAATTCGATGCAACTCCAGTTTTATATATAATATTGATTGATTG

ATTGTAAGTCCTAACACTTAAAACCTTGAAATTAATATTTTTATCTTACATAAATC
TTATTTAAATTATAAAAAATAAAATAAATTAATTATTCAATGAAAAACATGAGAG
AAAAAGATTCACATTTTAAAAACAAATTTTTAATATGGAATTCCTATCATATTGG
ATATATATAACATTTATTTTTTTCACATCAATTACCATTTTTAATTATTTTATTTT
ATTGACTATACTTTTATTAATTAGCGGAAAATACATGTGCAAAGCATGTACACTA
AATTAGTATATTAAGAAACACAAATTCCCTTAATGAAATATCGATGTAAGAACTT
TAATTTAAAATCCTTAACTCAATCACCCTTCGCATAAAAGAATGAAAACATTTTT
AATGATGGAAATATATTCATGTAATATCCTTTGCCATATCGTGTATTAAAGTATTA
AAATTGTTAGCTAAAATCTCTAGACCTATTA

>4113:emu_db:49288 ["4113:ncbi:49288 ['DQ231562.101809.103308_U Solanum tuberosum 16S ribosomal RNA, chloroplast']"]

TCTCATGGAGAGTTCGATCCTGGCTCAGGATGAACGCTGGCGGCATGCTTAACAC
ATGCAAGTCGGACGGGAAACACGGGAAACGGTGTTTCCAGTGGCGGACGGGTGA
GTAACGCGTAAGAACCTGCCCTTGGGAGGGGAACAACAGCTGGAAACGGCTGCT
AATACCCCGTAGGCTGAGGAGCAAAAGGAGGAATCCGCCCGAGGAGGGGCTCGC
GTCTGATTAGCTAGTTGGTGAGGCAATAGCTTACCAAGGCGATGATCAGTAGCTG
GTCCGAGAGGATGATCAGCCACACTGGGACTGAGACACGGCCCAGACTCCTACG
GGAGGCAGCAGTGGGGAATTTTCCGCAATGGGCGAAAGCCTGACGGAGCAATGC
CGCGTGGAGGTAGAAGGCCCACGGGTCGTGAACTTCTTTTCCCGGAGAAGAAGC
AATGACGGTATCTGGGGAATAAGCATCGGCTAACTCTGTGCCAGCAGCCGCGGTA
ATACAGAGGATGCAAGCGTTATCCGGAATGATTGGGCGTAAAGCGTCTGTAGGT
GGCTTTTTAAGTCCGCCGTCAAATCCCAGGGCTCAACCCTGGACAGGCGGTGGAA
ACTACCAAGCTGGAGTACGGTAGGGGCAGAGGGAATTTCCGGTGGAGCGGTGAA
ATGCGTAGAGATCGGAAAGAACACCAACGGCGAAAGCACTCTGCTGGGCCGACA
CTGACACTGAGAGACGAAAGCTAGGGGAGCGAATGGGATTAGATACCCCAGTAG
TCCTAGCCGTAAACGATGGATACTAGGCGCTGTGCGTATCGACCCGTGCAGTGCT
GTAGCTAACGCGTTAAGTATCCCGCCTGGGGAGTACGTTCGCAAGAATGAAACTC
AAAGGAATTGACGGGGGCCCGCACAAGCGGTGGAGCATGTGGTTTAATTCGATG
CAAAGCGAAGAACCTTACCAGGGCTTGACATGCCGCGAATCCTCTTGAAAGAGA
GGGGTGCCTTCGGGAACGCGGACACAGGTGGTGCATGGCTGTCTTCAGCTCGTGC
CGTAAGGTGTTGGGTTAAGTCCCGCAACGAGCGCAACCCTCGTGTTTAGTTGCCA
TCGTTGAGTTTGGAACCCTGAACAGACTGCCGGTGATAAGCCGGAGGAAGGTGA
GGATGACGTCAAGTCATCATGCCCCTTATGCCCTGGGCGACACACGTGCTACAAT
GGCCGGGACAAAGGGTCGCGATCCCGCGAGGGTGAGCTAACCCCAAAAACCCGT
CCTCAGTTCGGATTGCAGGCTGCAACTCGCCTGCATGAAGCCGGAATCGCCAGTA
ATCGCCGGTCAGCCATACGGCGGTGAATTCGTTCCCGGGCCTTGTACACACCGCC
CGTCACACTATGGGAGCTGGCCATGCCCGAAGTCGTTACCTTAACCGCAAGGAGG
GGGATGCCGAAGGCAGGGCTAGTGACTGGAGTGAAGTCGTAACAAGGTAGCCGT
ACTGGAAGGTGCGGCTGGATCACCTCCTTT

>4113:emu_db:49289 ["4113:ncbi:49289 ['AC217039.19117.20595_U Solanum tuberosum 16S ribosomal RNA, chloroplast']"]

AGAGTTCGATCCTGGCTCAGGATGAACGCTGGCGGCATGCTTAACACATGCAAGT
CGGACGGGAAACACGGGAAACGGTGTTTCCAATGGCGGATGGGTGAGTAATGCG
TAAGAACCTGCCCTTGGGAGGGGAACAACAGCTGGAAACGACTGCTAATACCCC
GTAGGCTAACGAGCAAAAGGAGGAATCCGCCCGAGGAGGGGCTCGCGTCTGATT
AGCTAGTTGGTGAGGCAATAGCTTACCAAGGCGATGATCAGTAGCTGGTCCGAG
AGGATGATCAGCCACACTGGGACTGAGACACAGCCCAGACTCCTACGGGAGGCA

GCAGTGGGGAATTTTCCGCAATGGGCGAAAGCCTGACGGAGCAATGCCGCGTGG
AGGTAGAAGGCCCACGGGTCGTGAACTTCTTTTCCCGGAGAAGAAGCAATGACG
GTATCTGGGGAATAAGCATCAGCTAACTCTGTGCCAGCAGCCGCGGTAATACAGA
GGATGCAAGCGTTATCCGGAATGATTGGGCGTAAAGCGTCTGTAGGTGGCTTTTT
AAGTCCGCCGTCAAATCCCAGGGCTCAACCCTGGACAGGCGGTGGAAACTACCA
AGCTGGAGTACGGTAGGGGCAGAGGGAATTTCCGGTGGAGCGGTGAAATGCGTA
GAGATCGGAAAGAACACCAACGGCGAAAGCACTCTGCTGGGCCGACACTGACAC
TGAGAGACGAAAGCTAGGGGCGCGAATGGGATTAGATACCCCAGTAGTCCTAGC
CGTAAACGATGGATACTAGGCGCTGTGCGTATCGACCCGTGCAGTGCTGTAGCTA
ACGCGTTAAGTATCCCGCCTGGGGAGTACGTTCGCAAGAATGAAACTCAAAGGA
ATTGACGGGGGCCCGCACAAGCGGTGGAGCATGTGGTTTAATTCGATGCAAAGC
GAAGAACCTTACCAGGGCTTGACATGCCGCGAATCCTCTTGAAAGAGAGAGGTG
CCTTCGGGAACGCGGACACAGGTGGTGCATGGCTGTCGTCAGCTCGTGCCGTAAG
GTGTTGGGTTAAGTCCCGCAACGAGCGCAACCCTTATGTTTAGTTGCCATCGTTG
AGTTTGGAACCCTGAACAGACTGCCGGTGATAAGCCGGAGGAAGGTGAGGATGA
CGTCAAGTCATCATGCCCCTTATGCCCTGGGCGACACACGTGCTACAATGGCCGG
GACAAAGGGTCGCGATCCCGCGAGGGTGAGCTAACCCCAAAAACCCGTCCTCAG
TTCGGATTGCAGGCTGCAACTCGCCTGCATGAAGCCGGAATCGCTAGTAATCGCC
GGTCAGCCATACGGCGGTGAATTCGTTCCCAGGCCTTGTACACACCGCCCGTCAC
ACTATGGGAGCTGGCCATGCCCGAAGTCGTTACCTTAACCGCAAGGAGGGGGAT
GCCGAAGGCAGGGCTAGTGACTGGAGTGAAGTCGTAACAAGGTAGCCGTACTGG
AAGGTGCGGCTG

>4113:emu_db:49290 ["4113:ncbi:49290 ['AJ252712.1.1008_U Solanum tuberosum 16S ribosomal RNA, chloroplast']"]

GAGTTTGATCATGGCTCAGGATGAACGCTGGCGGCATGCTTAACACATGCAAGTC
GGACGGGAAACACGGGAAACGGTGTTTCCAGTGGCGGACGGGTGAGTAACGCGT
AAGAACCTGCCCTTGGGAGGGGAACAACAGCTGGAAACGGCTGCTAATACCCCG
TAGGCTGAGGAGCAAAAGGAGGAATCCGCCCGAGGAGGGGCTCGCGTCTGATTA
GCTAGTTGGTGAGGCAATAGCTTACCAAGGCGATGATCAGTAGCTGGTCCGAGA
GGATGATCAGCCACACTGGGACTGAGACACGGCCCAGACTCCTACGGGAGGCAG
CAGTGGGGAATTTTCCGCAATGGGCGAAAGCCTGACGGAGCAATGCCGCGTGGA
GGTAGAAGGCCCACGGGTCGTGAACTTCTTTTCCCGGAGAAGAAGCAATGACGG
TATCTGGGGAATAAGCATCGGCTAACTCTGTGCCAGCAGCCGCGGTAATACAGAG
GATGCAAGCGTTATCCGGAATGATTGGGCGTAAAGCGTCTGTAGGTGGCTTTTTA
AGTCCGCCGTCAAATCCCAGGGCTCAACCCTGGACAGGCGGTGGAAACTACCAA
GCTGGAGTACGGTAGGGGCAGAGGGAATTTCCGGTGGAGCGGTGAAATGCGTAG
AGATCGGAAAGAACACCAACGGCGAAAGCACTCTGCTGGGCCGACACTGACACT
GAGAGACGAAAGCTAGGGGAGCGAATGGGATTAGATACCCCAGTAGTCCTAGCC
GTAAACGATGGATACTAGGCGCTGTGCGTATCGACCCGTGCAGTGCTGTAGCTAA
CGCGTTAAGTATCCCGCCTGGGGAGTACGTTCGCAAGAATGAAACTCAAAGGAA
TTGACGGGGGCCCGCACAAGCGGTGGAGCATGTGGTTTAATTCGATGCAAAGCG
AAGAACCTTACCAGGGCTTGACATGCCGCGAATCCTCTTGAAAGAGAGGGGTGC
CTTCGGGAACGCGGACACAGGTGGTGCATGGC

>4113:emu_db:49291 ["4113:ncbi:49291 ['JF772170.101749.103238_U Solanum tuberosum 16S ribosomal RNA, chloroplast']"]

TCTCATGGAGAGTTCGATCCTGGCTCAGGATGAACGCTGGCGGCATGCTTAACAC
ATGCAAGTCGGACGGGAAACACGGGAAACGGTGTTTCCAGTGGCGGACGGGTGA

GTAACGCGTAAGAACCTGCCCTTGGGAGGGGAACAACAGCTGGAAACGGCTGCT
AATACCCCGTAGGCTGAGGAGCAAAAGGAGGAATCCGCCCGAGGAGGGGCTCGC
GTCTGATTAGCTAGTTGGTGAGGCAATAGCTTACCAAGGCGATGATCAGTAGCTG
GTCCGAGAGGATGATCAGCCACACTGGGACTGAGACACGGCCCAGACTCCTACG
GGAGGCAGCAGTGGGGAATTTTCCGCAATGGGCGAAAGCCTGACGGAGCAATGC
CGCGTGGAGGTAGAAGGCCCACGGGTCGTGAACTTCTTTTCCCGGAGAAGAAGC
AATGACGGTATCTGGGGAATAAGCATCGGCTAACTCTGTGCCAGCAGCCGCGGTA
ATACAGAGGATGCAAGCGTTATCCGGAATGATTGGGCGTAAAGCGTCTGTAGGT
GGCTTTTTAAGTCCGCCGTCAAATCCCAGGGCTCAACCCTGGACAGGCGGTGGAA
ACTACCAAGCTGGAGTACGGTAGGGGCAGAGGGAATTTCCGGTGGAGCGGTGAA
ATGCGTAGAGATCGGAAAGAACACCAACGGCGAAAGCACTCTGCTGGGCCGACA
CTGACACTGAGAGACGAAAGCTAGGGGAGCGAATGGGATTAGATACCCCAGTAG
TCCTAGCCGTAAACGATGGATACTAGGCGCTGTGCGTATCGACCCGTGCAGTGCT
GTAGCTAACGCGTTAAGTATCCCGCCTGGGGAGTACGTTCGCAAGAATGAAACTC
AAAGGAATTGACGGGGGCCCGCACAAGCGGTGGAGCATGTGGTTTAATTCGATG
CAAAGCGAAGAACCTTACCAGGGCTTGACATGCCGCGAATCCTCTTGAAAGAGA
GGGGTGCCTTCGGGAACGCGGACACAGGTGGTGCATGGCTGTCGTCAGCTCGTGC
CGTAAGGTGTTGGGTTAAGTCCCGCAACGAGCGCAACCCTCGTGTTTAGTTGCCA
TCGTTGAGTTTGGAACCCTGAACAGACTGCCGGTGATAAGCCGGAGGAAGGTGA
GGATGACGTCAAGTCATCATGCCCCTTATGCCCTGGGCGACACACGTGCTACAAT
GGCCGGGACAAAGGGTCGCGATCCCGCGAGGGTGAGCTAACCCCAAAAACCCGT
CCTCAGTTCGGATTGCAGGCTGCAACTCGCCTGCATGAAGCCGGAATCGCTAGTA
ATCGCCGGTCAGCCATACGGCGGTGAATTCGTTCCCGGGCCTTGTACACACCGCC
CGTCACACTATGGGAGCTGGCCATGCCCGAAGTCGTTACCTTAACCGCAAGGAGG
GGGATGCCGAAGGCAGGGCTAGTGACTGGAGTGAAGTCGTAACAAGGTAGCCGT
ACTGGAAGGTGCGGCTGGAT

>4113:emu_db:49292 ["4113:ncbi:49292 ['DQ386163.101797.103296_U Solanum tuberosum 16S ribosomal RNA, chloroplast']"]

TCTCATGGAGAGTTCGATCCTGGCTCAGGATGAACGCTGGCGGCATGCTTAACAC
ATGCAAGTCGGACGGGAAACACGGGAAACGGTGTTTCCAGTGGCGGACGGGTGA
GTAACGCGTAAGAACCTGCCCTTGGGAGGGGAACAACAGCTGGAAACGGCTGCT
AATACCCCGTAGGCTGAGGAGCAAAAGGAGGAATCCGCCCGAGGAGGGGCTCGC
GTCTGATTAGCTAGTTGGTGAGGCAATAGCTTACCAAGGCGATGATCAGTAGCTG
GTCCGAGAGGATGATCAGCCACACTGGGACTGAGACACGGCCCAGACTCCTACG
GGAGGCAGCAGTGGGGAATTTTCCGCAATGGGCGAAAGCCTGACGGAGCAATGC
CGCGTGGAGGTAGAAGGCCCACGGGTCGTGAACTTCTTTTCCCGGAGAAGAAGC
AATGACGGTATCTGGGGAATAAGCATCGGCTAACTCTGTGCCAGCAGCCGCGGTA
ATACAGAGGATGCAAGCGTTATCCGGAATGATTGGGCGTAAAGCGTCTGTAGGT
GGCTTTTTAAGTCCGCCGTCAAATCCCAGGGCTCAACCCTGGACAGGCGGTGGAA
ACTACCAAGCTGGAGTACGGTAGGGGCAGAGGGAATTTCCGGTGGAGCGGTGAA
ATGCGTAGAGATCGGAAAGAACACCAACGGCGAAAGCACTCTGCTGGGCCGACA
CTGACACTGAGAGACGAAAGCTAGGGGAGCGAATGGGATTAGATACCCCAGTAG
TCCTAGCCGTAAACGATGGATACTAGGCGCTGTGCGTATCGACCCGTGCAGTGCT
GTAGCTAACGCGTTAAGTATCCCGCCTGGGGAGTACGTTCGCAAGAATGAAACTC
AAAGGAATTGACGGGGGCCCGCACAAGCGGTGGAGCATGTGGTTTAATTCGATG
CAAAGCGAAGAACCTTACCAGGGCTTGACATGCCGCGAATCCTCTTGAAAGAGA
GGGGTGCCTTCGGGAACGCGGACACAGGTGGTGCATGGCTGTCGTCAGCTCGTGC
CGTAAGGTGTTGGGTTAAGTCCCGCAACGAGCGCAACCCTCGTGTTTAGTTGCCA
TCGTTGAGTTTGGAACCCTGAACAGACTGCCGGTGATAAGCCGGAGGAAGGTGA

GGATGACGTCAAGTCATCATGCCCCTTATGCCCTGGGCGACACACGTGCTACAAT
GGCCGGGACAAAGGGTCGCGATCCCGCGAGGGTGAGCTAACCCCAAAAACCCGT
CCTCAGTTCGGATTGCAGGCTGCAACTCGCCTGCATGAAGCCGGAATCGCTAGTA
ATCGCCGGTCAGCCATACGGCGGTGAATTCGTTCCCGGGCCTTGTACACACCGCC
CGTCACACTATGGGAGCTGGCCATGCCCGAAGTCGTTACCTTAACCGCAAGGAGG
GGGATGCCGAAGGCAGGGCTAGTGACTGGAGTGAAGTCGTAACAAGGTAGCCGT
ACTGGAAGGTGCGGCTGGATCACCTCCTTT

>4113:emu_db:49293 ["4113:ncbi:49293 ['DQ386163.137738.139237_U Solanum tuberosum 16S ribosomal RNA, chloroplast']"]

TCTCATGGAGAGTTCGATCCTGGCTCAGGATGAACGCTGGCGGCATGCTTAACAC
ATGCAAGTCGGACGGGAAACACGGGAAACGGTGTTTCCAGTGGCGGACGGGTGA
GTAACGCGTAAGAACCTGCCCTTGGGAGGGGAACAACAGCTGGAAACGGCTGCT
AATACCCCGTAGGCTGAGGAGCAAAAGGAGGAATCCGCCCGAGGAGGGGCTCGC
GTCTGATTAGCTAGTTGGTGAGGCAATAGCTTACCAAGGCGATGATCAGTAGCTG
GTCCGAGAGGATGATCAGCCACACTGGGACTGAGACACGGCCCAGACTCCTACG
GGAGGCAGCAGTGGGGAATTTTCCGCAATGGGCGAAAGCCTGACGGAGCAATGC
CGCGTGGAGGTAGAAGGCCCACGGGTCGTGAACTTCTTTTCCCGGAGAAGAAGC
AATGACGGTATCTGGGGAATAAGCATCGGCTAACTCTGTGCCAGCAGCCGCGGTA
ATACAGAGGATGCAAGCGTTATCCGGAATGATTGGGCGTAAAGCGTCTGTAGGT
GGCTTTTTAAGTCCGCCGTCAAATCCCAGGGCTCAACCCTGGACAGGCGGTGGAA
ACTACCAAGCTGGAGTACGGTAGGGGCAGAGGGAATTTCCGGTGGAGCGGTGAA
ATGCGTAGAGATCGGAAAGAACACCAACGGCGAAAGCACTCTGCTGGGCCGACA
CTGACACTGAGAGACGAAAGCTAGGGGAGCGAATGGGATTAGATACCCCAGTAG
TCCTAGCCGTAAACGATGGATACTAGGCGCTGTGCGTATCGACCCGTGCAGTGCT
GTAGCTAACGCGTTAAGTATCCCGCCTGGGGAGTACGTTCGCAAGAATGAAACTC
AAAGGAATTGACGGGGGCCCGCACAAGCGGTGGAGCATGTGGTTTAATTCGATG
CAAAGCGAAGAACCTTACCAGGGCTTGACATGCCGCGAATCCTCTTGAAAGAGA
GGGGTGCCTTCGGGAACGCGGACACAGGTGGTGCATGGCTGTCGTCAGCTCGTGC
CGTAAGGTGTTGGGTTAAGTCCCGCAACGAGCGCAACCCTCGTGTTTAGTTGCCA
TCGTTGAGTTTGGAACCCTGAACAGACTGCCGGTGATAAGCCGGAGGAAGGTGA
GGATGACGTCAAGTCATCATGCCCCTTATGCCCTGGGCGACACACGTGCTACAAT
GGCCGGGACAAAGGGTCGCGATCCCGCGAGGGTGAGCTAACCCCAAAAACCCGT
CCTCAGTTCGGATTGCAGGCTGCAACTCGCCTGCATGAAGCCGGAATCGCTAGTA
ATCGCCGGTCAGCCATACGGCGGTGAATTCGTTCCCGGGCCTTGTACACACCGCC
CGTCACACTATGGGAGCTGGCCATGCCCGAAGTCGTTACCTTAACCGCAAGGAGG
GGGATGCCGAAGGCAGGGCTAGTGACTGGAGTGAAGTCGTAACAAGGTAGCCGT
ACTGGAAGGTGCGGCTGGATCACCTCCTTT

>4113:emu_db:49294 ["4113:ncbi:49294 ['JF772171.137925.139414_U Solanum tuberosum 16S ribosomal RNA, chloroplast']"]

AGTTCGATCCTGGCTCAGGATGAACGCTGGCGGCATGCTTAACACATGCAAGTCG
GACGGGAAACACGGGAAACGGTGTTTCCAGTGGCGGACGGGTGAGTAACGCGTA
AGAACCTGCCCTTGGGAGGGGAACAACAGCTGGAAACGGCTGCTAATACCCCGT
AGGCTGAGGAGCAAAAGGAGGAATCCGCCCGAGGAGGGGCTCGCGTCTGATTAG
CTAGTTGGTGAGGCAATAGCTTACCAAGGCGATGATCAGTAGCTGGTCCGAGAG
GATGATCAGCCACACTGGGACTGAGACACGGCCCAGACTCCTACGGGAGGCAGC
AGTGGGGAATTTTCCGCAATGGGCGAAAGCCTGACGGAGCAATGCCGCGTGGAG
GTAGAAGGCCCACGGGTCGTGAACTTCTTTTCCCGGAGAAGAAGCAATGACGGT

ATCTGGGGAATAAGCATCGGCTAACTCTGTGCCAGCAGCCGCGGTAATACAGAG
GATGCAAGCGTTATCCGGAATGATTGGGCGTAAAGCGTCTGTAGGTGGCTTTTA
AGTCCGCCGTCAAATCCCAGGGCTCAACCCTGGACAGGCGGTGGAAACTACCAA
GCTGGAGTACGGTAGGGGCAGAGGGAATTTCCGGTGGAGCGGTGAAATGCGTAG
AGATCGGAAAGAACACCAACGGCGAAAGCACTCTGCTGGGCCGACACTGACACT
GAGAGACGAAAGCTAGGGGAGCGAATGGGATTAGATACCCCAGTAGTCCTAGCC
GTAAACGATGGATACTAGGCGCTGTGCGTATCGACCCGTGCAGTGCTGTAGCTAA
CGCGTTAAGTATCCCGCCTGGGGAGTACGTTCGCAAGAATGAAACTCAAAGGAA
TTGACGGGGGCCCGCACAAGCGGTGGAGCATGTGGTTTAATTCGATGCAAAGCG
AAGAACCTTACCAGGGCTTGACATGCCGCGAATCCTCTTGAAAGAGAGGGGTGC
CTTCGGGAACGCGGACACAGGTGGTGCATGGCTGTCGTCAGCTCGTGCCGTAAGG
TGTTGGGTTAAGTCCCGCAACGAGCGCAACCCTCGTGTTTAGTTGCCATCGTTGA
GTTTGGAACCCTGAACAGACTGCCGGTGATAAGCCGGAGGAAGGTGAGGATGAC
GTCAAGTCATCATGCCCCTTATGCCCTGGGCGACACACGTGCTACAATGGCCGGG
ACAAAGGGTCGCGATCCCGCGAGGGTGAGCTAACCCCAAAAACCCGTCCTCAGT
TCGGATTGCAGGCTGCAACTCGCCTGCATGAAGCCGGAATCGCTAGTAATCGCCG
GTCAGCCATACGGCGGTGAATTCGTTCCCGGGCCTTGTACACACCGCCCGTCACA
CTATGGGAGCTGGCCATGCCCGAAGTCGTTACCTTAACCGCAAGGAGGGGGATG
CCGAAGGCAGGGCTAGTGACTGGAGTGAAGTCGTAACAAGGTAGCCGTACTGGA
AGGTGCGGCTGGATCACCTCCTTT

>4113:emu_db:49295 ["4113:ncbi:49295 ['JF772171.101981.103470_U Solanum tuberosum 16S ribosomal RNA, chloroplast']"]

TCTCATGGAGAGTTCGATCCTGGCTCAGGATGAACGCTGGCGGCATGCTTAACAC
ATGCAAGTCGGACGGGAAACACGGGAAACGGTGTTTCCAGTGGCGGACGGGTGA
GTAACGCGTAAGAACCTGCCCTTGGGAGGGGAACAACAGCTGGAAACGGCTGCT
AATACCCCGTAGGCTGAGGAGCAAAAGGAGGAATCCGCCCGAGGAGGGGCTCGC
GTCTGATTAGCTAGTTGGTGAGGCAATAGCTTACCAAGGCGATGATCAGTAGCTG
GTCCGAGAGGATGATCAGCCACACTGGGACTGAGACACGGCCCAGACTCCTACG
GGAGGCAGCAGTGGGGAATTTTCCGCAATGGGCGAAAGCCTGACGGAGCAATGC
CGCGTGGAGGTAGAAGGCCCACGGGTCGTGAACTTCTTTTCCCGGAGAAGAAGC
AATGACGGTATCTGGGGAATAAGCATCGGCTAACTCTGTGCCAGCAGCCGCGGTA
ATACAGAGGATGCAAGCGTTATCCGGAATGATTGGGCGTAAAGCGTCTGTAGGT
GGCTTTTTAAGTCCGCCGTCAAATCCCAGGGCTCAACCCTGGACAGGCGGTGGAA
ACTACCAAGCTGGAGTACGGTAGGGGCAGAGGGAATTTCCGGTGGAGCGGTGAA
ATGCGTAGAGATCGGAAAGAACACCAACGGCGAAAGCACTCTGCTGGGCCGACA
CTGACACTGAGAGACGAAAGCTAGGGGAGCGAATGGGATTAGATACCCCAGTAG
TCCTAGCCGTAAACGATGGATACTAGGCGCTGTGCGTATCGACCCGTGCAGTGCT
GTAGCTAACGCGTTAAGTATCCCGCCTGGGGAGTACGTTCGCAAGAATGAAACTC
AAAGGAATTGACGGGGGCCCGCACAAGCGGTGGAGCATGTGGTTTAATTCGATG
CAAAGCGAAGAACCTTACCAGGGCTTGACATGCCGCGAATCCTCTTGAAAGAGA
GGGGTGCCTTCGGGAACGCGGACACAGGTGGTGCATGGCTGTCGTCAGCTCGTGC
CGTAAGGTGTTGGGTTAAGTCCCGCAACGAGCGCAACCCTCGTGTTTAGTTGCCA
TCGTTGAGTTTGGAACCCTGAACAGACTGCCGGTGATAAGCCGGAGGAAGGTGA
GGATGACGTCAAGTCATCATGCCCCTTATGCCCTGGGCGACACACGTGCTACAAT
GGCCGGGACAAAGGGTCGCGATCCCGCGAGGGTGAGCTAACCCCAAAAACCCGT
CCTCAGTTCGGATTGCAGGCTGCAACTCGCCTGCATGAAGCCGGAATCGCTAGTA
ATCGCCGGTCAGCCATACGGCGGTGAATTCGTTCCCGGGCCTTGTACACACCGCC
CGTCACACTATGGGAGCTGGCCATGCCCGAAGTCGTTACCTTAACCGCAAGGAGG

GGGATGCCGAAGGCAGGGCTAGTGACTGGAGTGAAGTCGTAACAAGGTAGCCGT
ACTGGAAGGTGCGGCTGGAT

>4577:emu_db:49296 ["4577:ncbi:49296 ['AC194291.171087.172556_U Zea mays 16S ribosomal RNA, chloroplast']"]

AGAGTTCGATCCTGGCTCAGGATGAACGCTGGCGGCATGCTTAACACATGCAAGT
CGAACGGGAAGTGGTGTTTCCAGTGGCGAACGGGTGAGTAACGCGTAAGAACCT
GCCCTTGGGAGGGGAACAACAACTGGAAACGGTTGCTAATACCCCGTAGGCTGA
GGAGCAAAAGGAGAAATCCGCCCAAGGAGGGGCTCGCGTCTGATTAGCTAGTTG
GTGAGGCAATAGCTTACCAAGGCGATGATCAGTAGCTGGTCCGAGAGGATGATC
AGCCACACTGGGACTGAGACACGGCCCAGACTCCTACGGGAGGCAGCAGTGGGG
AATTTTCCGCAATGGGCGAAAGCCTGACGGAGCAATGCCGCGTGGAGGTGGAAG
GCCTACGGGTCGTCAACTTCTTTTCTCGGAGAAGAAACAATGACGGTATCTGAGG
AATAAGCATCGGCTAACTCTGTGCCAGCAGCCGCGGTAAGACAGAGGATGCAAG
CGTTATCCGGAATGATTGGGCGTAAAGCGTCTGTAGGTGGCTTTTCAAGTCCGCC
GTCAAATCCCAGGGCTCAACCCTGGACAGGCGGTGGAAACTACCAAGCTGGAGT
ACGGTAGGGGCAGAGGGAATTTCCGGTGGAGCGGTGAAATGCATTGAGATCGGA
AAGAACACCAACGGCGAAAGCACTCTGCTGGGCCGACACTGACACTGAGAGACG
AAAGCTAGGGGAGCAAATGGGATTAGAGACCCCAGTAGTCCTAGCCGTAAACGA
TGGATACTAGGTGCTGTGCGACTCGACCCGTGCAGTGCTGTAGCTAACGCGTTAA
GTATCCCGCCTGGGGAGTACGTTCGCAAGAATGAAACTCAAAGGAATTGACGGG
GGCCCGCACAAGCGGTGGAGCATGTGGTTTAATTCGATGCAAAGCGAAGAACCT
TACCAGGGCTTGACATGCCGCGAATCCTCTTGAAAGAGAGGGGTGCCCTCGGGA
ACGCGGACACAGGTGGTGCATGGCTGTCGTCAGCTCGTGCCGTAAGGTGTTGGGT
TAAGTCTCGCAACGAGCGCAACCCTCGTGTTTAGTTGCCACTATGAGTTTGGAAC
CCTGAACAGACCGCCGGTGTTAAGCCGGAGGAAGGAGAGGATGAGGCCAAGTCA
TCATGCCCCTTATGCCCTGGGCGACACACGTGCTACAATGGGCGGGACAAAGGGT
CGCGATCTCGCGAGGGTGAGCTAACTCCAAAAACCCGTCCTCAGTTCGGATTGCA
GGCTGCAACTCGCCTGCATGAAGCAGGAATCGCTAGTAATCGCCGGTCAGCCATA
CGGCGGTGAATCCGTTCCCGGGCCTTGTACACACCGCCCGTCACACTATAGGAGC
TGGCCAGGTTTGAAGTCATTACCCTTAACCGTAAGGAGGGGGATGCCTAAGGCTA
GGCTTGCGACTGGAGTGAAGTCGTAACAAGGTAGCCGTACTGGAAGGTGCGGCT
G

>4577:emu_db:49297 ["4577:ncbi:49297 ['AY928077.95200.96669_U Zea mays 16S ribosomal RNA, chloroplast']"]

AGAGTTCGATCCTGGCTCAGGATGAACGCTGGCGGCATGCTTAACACATGCAAGT
CGAACGGGAAGTGGTGTTTCCAGTGGCGAACGGGTGAGTAACGCGTAAGAACCT
GCCCTTGGGAGGGGAACAACAACTGGAAACGGTTGCTAATACCCCGTAGGCTGA
GGAGCAAAAGGAGAAATCCGCCCAAGGAGGGGCTCGCGTCTGATTAGCTAGTTG
GTGAGGCAATAGCTTACCAAGGCGATGATCAGTAGCTGGTCCGAGAGGATGATC
AGCCACACTGGGACTGAGACACGGCCCAGACTCCTACGGGAGGCAGCAGTGGGG
AATTTTCCGCAATGGGCGAAAGCCTGACGGAGCAATGCCGCGTGGAGGTGGAAG
GCCTACGGGTCGTCAACTTCTTTTCTCGGAGAAGAAACAATGACGGTATCTGAGG
AATAAGCATCGGCTAACTCTGTGCCAGCAGCCGCGGTAAGACAGAGGATGCAAG
CGTTATCCGGAATGATTGGGCGTAAAGCGTCTGTAGGTGGCTTTTCAAGTCCGCC
GTCAAATCCCAGGGCTCAACCCTGGACAGGCGGTGGAAACTACCAAGCTGGAGT
ACGGTAGGGGCAGAGGGAATTTCCGGTGGAGCGGTGAAATGCATTGAGATCGGA
AAGAACACCAACGGCGAAAGCACTCTGCTGGGCCGACACTGACACTGAGAGACG

AAAGCTAGGGGAGCAAATGGGATTAGAGACCCCAGTAGTCCTAGCCGTAAACGA
TGGATACTAGGTGCTGTGCGACTCGACCCGTGCAGTGCTGTAGCTAACGCGTTAA
GTATCCCGCCTGGGGAGTACGTTCGCAAGAATGAAACTCAAAGGAATTGACGGG
GGCCCGCACAAGCGGTGGAGCATGTGGTTTAATTCGATGCAAAGCGAAGAACCT
TACCAGGGCTTGACATGCCGCGAATCCTCTTGAAAGAGAGGGGTGCCCTCGGGA
ACGCGGACACAGGTGGTGCATGGCTGTCGTCAGCTCGTGCCGTAAGGTGTTGGGT
TAAGTCTCGCAACGAGCGCAACCCTCGTGTTTAGTTGCCACTATGAGTTTGGAAC
CCTGAACAGACCGCCGGTGTTAAGCCGGAGGAAGGAGAGGATGAGGCCAAGTCA
TCATGCCCCTTATGCCCTGGGCGACACACGTGCTACAATGGGCGGGACAAAGGGT
CGCGATCTCGCGAGGGTGAGCTAACTCCAAAAACCCGTCCTCAGTTCGGATTGCA
GGCTGCAACTCGCCTGCATGAAGCAGGAATCGCTAGTAATCGCCGGTCAGCCATA
CGGCGGTGAATCCGTTCCCGGGCCTTGTACACACCGCCCGTCACACTATAGGAGC
TGGCCAGGTTTGAAGTCATTACCCTTAACCGTAAGGAGGGGGATGCCTAAGGCTA
GGCTTGCGACTGGAGTGAAGTCGTAACAAGGTAGCCGTACTGGAAGGTGCGGCT
G

>4577:emu_db:49298 ["4577:ncbi:49298 ['AC209810.3832.5301_U Zea mays 16S ribosomal RNA, chloroplast']"]

AGAGTTCGATCCTGGCTCAGGATGAACGCTGGCGGCATGCTTAACACATGCAAGT
CGAACGGGAAGTGGTGTTTCCAGTGGCGAACGGGTGAGTAACGCGTAAGAACCT
GCCCTTGGGAGGGGAACAACAACTGGAAACGGTTGCTAATACCCCGTAGGCTGA
GGAGCAAAAGGAGAAATCCGCCCAAGGAGGGGCTCGCGTCTGATTAGCTAGTTG
GTGAGGCAATAGCTTACCAAGGCGATGATCAGTAGCTGGTCCGAGAGGATGATC
AGCCACACTGGGACTGAGACACGGCCCAGACTCCTACGGGAGGCAGCAGTGGGG
AATTTTCCGCAATGGGCGAAAGCCTGACGGAGCAATGCCGCGTGGAGGTGGAAG
GCCTACGGGTCGTCAACTTCTTTTCTCGGAGAAGAAACAATGACGGTATCTGAGG
AATAAGCATCGGCTAACTCTGTGCCAGCAGCCGCGGTAAGACAGAGGATGCAAG
CGTTATCCGGAATGATTGGGCGTAAAGCGTCTGTAGGTGGCTTTTCAAGTCCGCC
GTCAAATCCCAGGGCTCAACCCTGGACAGGCGGTGGAAACTACCAAGCTGGAGT
ACGGTAGGGGCAGAGGGAATTTCCGGTGGAGCGGTGAAATGCATTGAGATCGGA
AAGAACACCAACGGCGAAAGCACTCTGCTGGGCCGACACTGACACTGAGAGACG
AAAGCTAGGGGAGCAAATGGGATTAGAGACCCCAGTAGTCCTAGCCGTAAACGA
TGGATACTAGGTGCTGTGCGACTCGACCCGTGCAGTGCTGTAGCTAACGCGTTAA
GTATCCCGCCTGGGGAGTACGTTCGCAAGAATGAAACTCAAAGGAATTGACGGG
GGCCCGCACAAGCGGTGGAGCATGTGGTTTAATTCGATGCAAAGCGAAGAACCT
TACCAGGGCTTGACATGCCGCGAATCCTCTTGAAAGAGAGGGGTGCCCTCGGGA
ACGCGGACACAGGTGGTGCATGGCTGTCGTCAGCTCGTGCCGTAAGGTGTTGGGT
TAAGTCTCGCAACGAGCGCAACCCTCGTGTTTAGTTGCCACTATGAGTTTGGAAC
CCTGAACAGACCGCCGGTGTTAAGCCGGAGGAAGGAGAGGATGAGGCCAAGTCA
TCATGCCCCTTATGCCCTGGGCGACACACGTGCTACAATGGGCGGGACAAAGGGT
CGCGATCTCGCGAGGGTGAGCTAACTCCAAAAACCCGTCCTCAGTTCGGATTGCA
GGCTGCAACTCGTCTGCATGAAGCAGGAATCGCTAGTAATCGCCGGTCAGCCATA
CGGCGGTGAATCCGTTCCCGGGCCTTGTACACACCGCCCGTCACACTATAGGAGC
TGGCCAGGTTTGAAGTCATTACCCTTAACCGTAAGGAGGGGGATGCCTAAGGCTA
GGCTTGCGACTGGAGTGAAGTCGTAACAAGGTAGCCGTACTGGAAGGTGCGGCT
G

>4577:emu_db:49299 ["4577:ncbi:49299 ['Z00028.1514.3004_U Zea mays 16S ribosomal RNA, chloroplast']"]

TCTCATGGAGAGTTCGATCCTGGCTCAGGATGAACGCTGGCGGCATGCTTAACAC
ATGCAAGTCGAACGGAAGTGGTGTTTCCAGTGGCGAACGGGTGAGTAACGCGT
AAGAACCTGCCCTTGGGAGGGGAACAACAACTGGAAACGGTTGCTAATACCCCG
TAGGCTGAGGAGCAAAAGGAGAAATCCGCCCAAGGAGGGGCTCGCGTCTGATTA
GCTAGTTGGTGAGGCAATAGCTTACCAAGGCGATGATCAGTAGCTGGTCCGAGA
GGATGATCAGCCACACTGGGACTGAGACACGGCCCAGACTCCTACGGGAGGCAG
CAGTGGGGAATTTTCCGCAATGGGCGAAAGCCTGACGGAGCAATGCCGCGTGGA
GGTGGAAGGCCTACGGGTCGTCAACTTCTTTTCTCGGAGAAGAAACAATGACGGT
ATCTGAGGAATAAGCATCGGCTAACTCTGTGCCAGCAGCCGCGGTAAGACAGAG
GATGCAAGCGTTATCCGGAATGATTGGGCGTAAAGCGTCTGTAGGTGGCTTTTCA
AGTCCGCCGTCAAATCCCAGGGCTCAACCCTGGACAGGCGGTGGAAACTACCAA
GCTGGAGTACGGTAGGGGCAGAGGGAATTTCCGGTGGAGCGGTGAAATGCATTG
AGATCGGAAAGAACACCAACGGCGAAAGCACTCTGCTGGGCCGACACTGACACT
GAGAGACGAAAGCTAGGGGAGCAAATGGGATTAGAGACCCCAGTAGTCCTAGCC
GTAAACGATGGATACTAGGTGCTGTGCGACTCGACCCGTGCAGTGCTGTAGCTAA
CGCGTTAAGTATCCCGCCTGGGGAGTACGTTCGCAAGAATGAAACTCAAAGGAA
TTGACGGGGGCCCGCACAAGCGGTGGAGCATGTGGTTTAATTCGATGCAAGGCG
AAGAACCTTACCAGGGCTTGACATGCCGCGAATCCTCTTGAAAGAGAGGGGTGC
CCTCGGGAACGCGGACACAGGTGGTGCATGGCTGTCGTCAGCTCGTGCCGTAAGG
TGTTGGGTTAAGTCTCGCAACGAGCGCAACCCTCGTGTTTAGTTGCCACTATGAG
TTTGGAACCCTGAACAGACCGCCGGTGTTAAGCCGGAGGAAGGAGAGGATGAGG
CCAAGTCATCATGCCCCTTATGCCCTGGGCGACACACGTGCTACAATGGGCGGGA
CAAAGGGTCGCGATCTCGCGAGGGTGAGCTAACTCCAAAAACCCGTCCTCAGTTC
GGATTGCAGGCTGCAACTCGCCTGCATGAAGCAGGAATCGCTAGTAATCGCCGGT
CAGCCATACGGCGGCGAATCCGTTCCCGGGCCTTGTACACACCGCCCGTCACACT
ATAGGAGCTGGCCAGGTTTGAAGTCATTACCCTTAACCGTAAGGAGGGGGATGCC
TAAGGCTAGGCTTGCGACTGGAGTGAAGTCGTAACAAGGTAGCCGTACTGGAAG
GTGCGGCTGGATCACCTCCTTT

>4577:emu_db:49300 ["4577:ncbi:49300 ['X86563.95161.96651_U Zea mays 16S ribosomal RNA, chloroplast']"]

TCTCATGGAGAGTTCGATCCTGGCTCAGGATGAACGCTGGCGGCATGCTTAACAC
ATGCAAGTCGAACGGAAGTGGTGTTTCCAGTGGCGAACGGGTGAGTAACGCGT
AAGAACCTGCCCTTGGGAGGGGAACAACAACTGGAAACGGTTGCTAATACCCCG
TAGGCTGAGGAGCAAAAGGAGAAATCCGCCCAAGGAGGGGCTCGCGTCTGATTA
GCTAGTTGGTGAGGCAATAGCTTACCAAGGCGATGATCAGTAGCTGGTCCGAGA
GGATGATCAGCCACACTGGGACTGAGACACGGCCCAGACTCCTACGGGAGGCAG
CAGTGGGGAATTTTCCGCAATGGGCGAAAGCCTGACGGAGCAATGCCGCGTGGA
GGTGGAAGGCCTACGGGTCGTCAACTTCTTTTCTCGGAGAAGAAACAATGACGGT
ATCTGAGGAATAAGCATCGGCTAACTCTGTGCCAGCAGCCGCGGTAAGACAGAG
GATGCAAGCGTTATCCGGAATGATTGGGCGTAAAGCGTCTGTAGGTGGCTTTTCA
AGTCCGCCGTCAAATCCCAGGGCTCAACCCTGGACAGGCGGTGGAAACTACCAA
GCTGGAGTACGGTAGGGGCAGAGGGAATTTCCGGTGGAGCGGTGAAATGCATTG
AGATCGGAAAGAACACCAACGGCGAAAGCACTCTGCTGGGCCGACACTGACACT
GAGAGACGAAAGCTAGGGGAGCAAATGGGATTAGAGACCCCAGTAGTCCTAGCC
GTAAACGATGGATACTAGGTGCTGTGCGACTCGACCCGTGCAGTGCTGTAGCTAA
CGCGTTAAGTATCCCGCCTGGGGAGTACGTTCGCAAGAATGAAACTCAAAGGAA
TTGACGGGGGCCCGCACAAGCGGTGGAGCATGTGGTTTAATTCGATGCAAGGCG
AAGAACCTTACCAGGGCTTGACATGCCGCGAATCCTCTTGAAAGAGAGGGGTGC
CCTCGGGAACGCGGACACAGGTGGTGCATGGCTGTCGTCAGCTCGTGCCGTAAGG

TGTTGGGTTAAGTCTCGCAACGAGCGCAACCCTCGTGTTTAGTTGCCACTATGAG
TTTGGAACCCTGAACAGACCGCCGGTGTTAAGCCGGAGGAAGGAGAGGATGAGG
CCAAGTCATCATGCCCCTTATGCCCTGGGCGACACACGTGCTACAATGGGCGGGA
CAAAGGGTCGCGATCTCGCGAGGGTGAGCTAACTCCAAAAACCCGTCCTCAGTTC
GGATTGCAGGCTGCAACTCGCCTGCATGAAGCAGGAATCGCTAGTAATCGCCGGT
CAGCCATACGGCGGCGAATCCGTTCCCGGGCCTTGTACACACCGCCCGTCACACT
ATAGGAGCTGGCCAGGTTTGAAGTCATTACCCTTAACCGTAAGGAGGGGGATGCC
TAAGGCTAGGCTTGCGACTGGAGTGAAGTCGTAACAAGGTAGCCGTACTGGAAG
GTGCGGCTGGATCACCTCCTTT

>4577:emu_db:49301 ["4577:ncbi:49301 ['AY928077.126168.127637_U Zea mays 16S ribosomal RNA, chloroplast']"]

AGAGTTCGATCCTGGCTCAGGATGAACGCTGGCGGCATGCTTAACACATGCAAGT
CGAACGGGAAGTGGTGTTTCCAGTGGCGAACGGGTGAGTAACGCGTAAGAACCT
GCCCTTGGGAGGGGAACAACAACTGGAAACGGTTGCTAATACCCCGTAGGCTGA
GGAGCAAAAGGAGAAATCCGCCCAAGGAGGGGCTCGCGTCTGATTAGCTAGTTG
GTGAGGCAATAGCTTACCAAGGCGATGATCAGTAGCTGGTCCGAGAGGATGATC
AGCCACACTGGGACTGAGACACGGCCCAGACTCCTACGGGAGGCAGCAGTGGGG
AATTTTCCGCAATGGGCGAAAGCCTGACGGAGCAATGCCGCGTGGAGGTGGAAG
GCCTACGGGTCGTCAACTTCTTTTCTCGGAGAAGAAACAATGACGGTATCTGAGG
AATAAGCATCGGCTAACTCTGTGCCAGCAGCCGCGGTAAGACAGAGGATGCAAG
CGTTATCCGGAATGATTGGGCGTAAAGCGTCTGTAGGTGGCTTTTCAAGTCCGCC
GTCAAATCCCAGGGCTCAACCCTGGACAGGCGGTGGAAACTACCAAGCTGGAGT
ACGGTAGGGGCAGAGGGAATTTCCGGTGGAGCGGTGAAATGCATTGAGATCGGA
AAGAACACCAACGGCGAAAGCACTCTGCTGGGCCGACACTGACACTGAGAGACG
AAAGCTAGGGGAGCAAATGGGATTAGAGACCCCAGTAGTCCTAGCCGTAAACGA
TGGATACTAGGTGCTGTGCGACTCGACCCGTGCAGTGCTGTAGCTAACGCGTTAA
GTATCCCGCCTGGGGAGTACGTTCGCAAGAATGAAACTCAAAGGAATTGACGGG
GGCCCGCACAAGCGGTGGAGCATGTGGTTTAATTCGATGCAAAGCGAAGAACCT
TACCAGGGCTTGACATGCCGCGAATCCTCTTGAAAGAGAGGGGTGCCCTCGGGA
ACGCGGACACAGGTGGTGCATGGCTGTCGTCAGCTCGTGCCGTAAGGTGTTGGGT
TAAGTCTCGCAACGAGCGCAACCCTCGTGTTTAGTTGCCACTATGAGTTTGGAAC
CCTGAACAGACCGCCGGTGTTAAGCCGGAGGAAGGAGAGGATGAGGCCAAGTCA
TCATGCCCCTTATGCCCTGGGCGACACACGTGCTACAATGGGCGGGACAAAGGGT
CGCGATCTCGCGAGGGTGAGCTAACTCCAAAAACCCGTCCTCAGTTCGGATTGCA
GGCTGCAACTCGCCTGCATGAAGCAGGAATCGCTAGTAATCGCCGGTCAGCCATA
CGGCGGTGAATCCGTTCCCGGGCCTTGTACACACCGCCCGTCACACTATAGGAGC
TGGCCAGGTTTGAAGTCATTACCCTTAACCGTAAGGAGGGGGATGCCTAAGGCTA
GGCTTGCGACTGGAGTGAAGTCGTAACAAGGTAGCCGTACTGGAAGGTGCGGCT
G

>4577:emu_db:49302 ["4577:ncbi:49302 ['AC211326.73983.75454_U Zea mays 16S ribosomal RNA, chloroplast']"]

AGAGTTCGATCCTGGCTCAGGATGAACGCTGGCGGCATGCTAAACACATGCAAGT
CGAACGGGAAGTGGTGTTTCCAGTGGCGAACGGGTGAGTAACGCGTAAGAACCT
GCCCTTGGGAGGGGAACAACAACTGGAAATGGTTGCTAATACCCCGTAGGCTGA
GGAGCAAAAGGAGAAATCCGCCCAAGGAGGGGCTCGCGTCTGATTAGCTAGTTG
GTGAGGCAATAGCTTACCAAGGCGATGATCAGTAGCTGGTCCGAGAGGATGATC
AGCCACACTGGGACTGAGACACGGCCCAGACTCCTACGGGAGGCAGCAGTGGGG

AATTTTCCGCAATGGGCGAAAGCCTGACGGAGCAATGCCGCGTGGAGGTGGAAG
GCCTACGGGTCGTCAACTTCTTTTCTCGGAGAAGAAACAATGACGGTATCTGAGG
AATAAGCATCGGCTAACTCTGTGCCAGCAGCCGCGGTAAGACAGAGGATGCAAG
CGTTATCCGGAATGATTGGGCATAAAGCGTCTGTAGGTGGCTTTTCAAGTCCGCC
GTCAAATCCCAGGGCTCAACCCTGGACAGGCGGTGGAAACTACCAAGCTGGAGT
ACGGTAGGGGCAGAGGGAATTTCCGGTGGAGCGGTGAAATGCATTGAGATCGGA
AAGAACACCAACGGCGAAAGCACTCTGCTGGGCCGACACTGACACTGAGAGACG
AAAGCTAGGGGAGCAAATGGGATTAGAGACCCCAGTAGTCCTAGCCGTAAACGA
TGGATACTAGGTGTTGTGCGACTCGACCCGTGCAGTGCTGTAGCTAACGCGTTAA
GTATCCCGCCTGGGGAGTACGTTCGCAAGAATGAAACTCAAAGGAATTGACGGG
GGCCCGCACAAGCGGTGGAGCATGTGGTTTAATTCGATGCAAAGCGAAGAACCT
TACCAGGGCTTGACATGCCGCGAATCCTCTTGAAAGAGAGGGGTGCCCTCGGGA
ACGCGGACACAGGTGGTGCATGGCTGTCGTCAGCTCGTGCCGTAAGGTGTTGGGT
TAAGTCTCGCAACGAGCGCAACCCTCGTGTTTAGTTGCCACTATGAGTTTGGGAA
CCCTGAACAGACCGCCGGTGTTAAGCCAGAGGAAGGAGAGGATGAGGCCAAGTC
ATCATGCCCCTTATGCCCTGGGCGACACACGTGCTACAATGGGCGGGACAAAGG
GTCGCGATCTCGCGAGGGTGAGCTAACTCCAAAAAACCCGTCCTCAGTTCGGATT
GTAGGCTGCAACTCGCCTGCATGAAGCAGGAATCGCTAGTAATCGCCGGTCAGCC
ATACGGCGGTGAATCCGTTCCCGGGCCTTGTACACACCGCCCGTCACACTATAGG
AGCTGGCCAGGTTTGAAGTCATTACCCTTAACCGTAAGGAGGGGGATGCCTAAGG
CTAGGCTTGCGACTGGAGTGAAGTCGTAACAAGGTAGCCGTACTGGAAGGTGCG
GCTG

>4577:emu_db:49303 ["4577:ncbi:49303 ['AC214801.171736.173205_U Zea mays 16S ribosomal RNA, chloroplast']"]

AGAGTTCGATCCTGGCTCAGGATGAACGCTGGCGGCATGCTTAACACATGCAAGT
CGAACGGGAAGTGGTGTTTCCAGTGGCGAACGGGTGAGTAACGCGTAAGAACCT
GCCCTTGGGAGGGGAACAACAACTGGAAACGGTTGCTAATACCCCGTAGGCTGA
GGAGCAAAAGGAGAAATCCGCCCAAGGAGGGGCTCGCGTCTGATTAGCTAGTTG
GTGAGGCAATAGCTTACCAAGGCGATGATCAGTAGCTGGTCCGAGAGGATGATC
AGCCACACTGGGACTGAGACACGGCCCAGACTCCTACGGGAGGCAGCAGTGGGG
AATTTTCCGCAATGGGCGAAAGCCTGACGGAGCAATGCCGCGTGGAGGTGGAAG
GCCTACGGGTCGTCAACTTCTTTTCTCGGAGAAGAAACAATGACGGTATCTGAGG
AATAAGCATCGGCTAACTCTGTGCCAGCAGCCGCGGTAAGACAGAGGATGCAAG
CGTTATCCGGAATGATTGGGCGTAAAGCGTCTGTAGGTGGCTTTTCAAGTCCGCC
GTCAAATCCCAGGGCTCAACCCTGGACAGGCGGTGGAAACTACCAAGCTGGAGT
ACGGTAGGGGCAGAGGGAATTTCCGGTGGAGCGGTGAAATGCATTGAGATCGGA
AAGAACACCAACGGCGAAAGCACTCTGCTGGGCCGACACTGACACTGAGAGACG
AAAGCTAGGGGAGCAAATGGGATTAGAGACCCCAGTAGTCCTAGCCGTAAACGA
TGGATACTAGGTGCTGTGCGACTCGACCCGTGCAGTGCTGTAGCTAACGCGTTAA
GTATCCCGCCTGGGGAGTACGTTCGCAAGAATGAAACTCAAAGGAATTGACGGG
GGCCCGCACAAGCGGTGGAGCATGTGGTTTAATTCGATGCAAAGCGAAGAACCT
TACCAGGGCTTGACATGCCGCGAATCCTCTTGAAAGAGAGGGGTGCCCTCGGGA
ACGCGGACACAGGTGGTGCATGGCTGTCGTCAGCTCGTGCCGTAAGGTGTTGGGT
TAAGTCTCGCAACGAGCGCAACCCTCGTGTTTAGTTGCCACTATGAGTTTGGAAC
CCTGAACAGACCGCCGGTGTTAAGCCGGAGGAAGGAGAGGATGAGGCCAAGTCA
TCATGCCCCTTATGCCCTGGGCGACACACGTGCTACAATGGGCGGGACAAAGGGT
CGCGATCTCGCGAGGGTGAGCTAACTCCAAAAACCCGTCCTCAGTTCGGATTGCA
GGCTGCAACTCGCCTGCATGAAGCAGGAATCGCTAGTAATCGCCGGTCAGCCATA
CGGCGGTGAATCCGTTCCCGGGCCTTGTACACACCGCCCGTCACACTATAGGAGC

TGGCCAGGTTTGAAGTCATTACCCTTAACCGTAAGGAGGGGGATGCCTAAGGCTA
GGCTTGCGACTGGAGTGAAGTCGTAACAAGGTAGCCGTACTGGAAGGTGCGGCT
G

>4577:emu_db:49304 ["4577:ncbi:49304 ['AC187397.54765.56236_U Zea mays 16S ribosomal RNA, chloroplast']"]

AGAGTTCGATCCTGGCTCAGGATGAACGCTGGCGGCATGCTTAACACATGCAAGT
CGAACGGGAAGTGGTGTTTCCAGTGGCGAACGGGTGAGTAACGCGTAAGAACCT
GCCCTTGGGAGGGGAACAACAACTGGAAACGGTTGCTAATACCCCGTAGGCTGA
GGAGCAAAAGGAGAAATCCGCCCAAGGAGGGGCTCGCGTCTGATTAGCTAGTTG
GTGAGGCAATAGCTTACCAAGGCGATGATCAGTAGCTGGTCCGAGAGGATGATC
AGCCACACTGGGACTGAGACACGGCCCAGACTCCTACGGGAGGCAGCAGTGGGG
AATTTTCCGCAATGGGCGAAAGCCTGACGGAGCAATGCCGCGTGGAGGTGGAAG
GCCTACGGGTCGTCAACTTCTTTTCTCGGAGAAGAAACAATGACGGTATCTGAGG
AATAAGCATCGGCTAACTCTGTGCCAGCAGCCGCGGTAAGACAGAGGATGCAAG
CGTTATCCGGAATGATTGGGCGTAAAGCGTCTGTAGGTGGCTTTTCAAGTCCGCC
GTCAAATCCCAGGGCTCAACCCTGGACAGGCGGTGGAAACTACCAAGCTGGAGT
ACGGTAGGGGCAGAGGGAATTTCCGGTGGAGCGGTGAAATGCATTGAGATCGGA
AAGAACACCAACGGCGAAAGCACTCTGCTGGGCCGACACTGACACTGAGAGACG
AAAGCTAGGGGAGCAAATGGGATTAGAGACCCCAGTAGTCCTAGCCGTAAACGA
TGGATACTAGGTGCTGTGCGACTCGACCCGTGCAGTGCTGTAGCTAACGCGTTAA
GTATCCCGCCTGGGGAGTACGTTCGCAAGAATGAAACTCAAAGGGAATTGACGG
GGGCCCGCACAAGCGGTGGAGCATGTGGTTTAATTCGATGCAAAGCGAAGAACC
TTACCAGGGCTTGACATGCCGCGAATCCTCTTGAAAGAGAGGGGTGCCCTCGGGA
ACGCGGACACAGGTGGTGCATGGCTGTCGTCAGCTCGTGCCGTAAGGTGTTGGGT
TAAGTCTCGCAACGAGCGCAACCCTCGTGTTTAGTTGCCACTATGAGTTTGGAAC
CCTGAACAGACCGCCGGTGTTAAGCCGGAGGAAGGAGAGGATGAGGCCAAGTCA
TCATGCCCCTTATGCCCTGCGCGACACACGTGCTACAATGGGCGGGACAAAAGGG
TCGCGATCTCGCGAGGGTGAGCTAACTCCAAAAACCCGTCCTCAGTTCGGATTGC
AGGCTGCAACTCGCCTGCATGAAGCAGGAATCGCTAGTAATCGCCGGTCAGCCAT
ACGGCGGTGAATCCGTTCCCGGGCCTTGTACACACCGCCCGTCACACTATAGGAG
CTGGCCAGGTTTGAAGTCATTACCCTTAACCGTAAGGAGGGGGATGCCTAAGGCT
AGGCTTGCGACTGGAGTGAAGTCGTAACAAGGTAGCCGTACTGGAAGGTGCGGC
TG

>4577:emu_db:49305 ["4577:ncbi:49305 ['X86563.126086.127576_U Zea mays 16S ribosomal RNA, chloroplast']"]

TCTCATGGAGAGTTCGATCCTGGCTCAGGATGAACGCTGGCGGCATGCTTAACAC
ATGCAAGTCGAACGGGAAGTGGTGTTTCCAGTGGCGAACGGGTGAGTAACGCGT
AAGAACCTGCCCTTGGGAGGGGAACAACAACTGGAAACGGTTGCTAATACCCCG
TAGGCTGAGGAGCAAAAGGAGAAATCCGCCCAAGGAGGGGCTCGCGTCTGATTA
GCTAGTTGGTGAGGCAATAGCTTACCAAGGCGATGATCAGTAGCTGGTCCGAGA
GGATGATCAGCCACACTGGGACTGAGACACGGCCCAGACTCCTACGGGAGGCAG
CAGTGGGGAATTTTCCGCAATGGGCGAAAGCCTGACGGAGCAATGCCGCGTGGA
GGTGGAAGGCCTACGGGTCGTCAACTTCTTTTCTCGGAGAAGAAACAATGACGGT
ATCTGAGGAATAAGCATCGGCTAACTCTGTGCCAGCAGCCGCGGTAAGACAGAG
GATGCAAGCGTTATCCGGAATGATTGGGCGTAAAGCGTCTGTAGGTGGCTTTTCA
AGTCCGCCGTCAAATCCCAGGGCTCAACCCTGGACAGGCGGTGGAAACTACCAA
GCTGGAGTACGGTAGGGGCAGAGGGAATTTCCGGTGGAGCGGTGAAATGCATTG

AGATCGGAAAGAACACCAACGGCGAAAGCACTCTGCTGGGCCGACACTGACACT
GAGAGACGAAAGCTAGGGGAGCAAATGGGATTAGAGACCCCAGTAGTCCTAGCC
GTAAACGATGGATACTAGGTGCTGTGCGACTCGACCCGTGCAGTGCTGTAGCTAA
CGCGTTAAGTATCCCGCCTGGGGAGTACGTTCGCAAGAATGAAACTCAAAGGAA
TTGACGGGGGCCCGCACAAGCGGTGGAGCATGTGGTTTAATTCGATGCAAGGCG
AAGAACCTTACCAGGGCTTGACATGCCGCGAATCCTCTTGAAAGAGAGGGGTGC
CCTCGGGAACGCGGACACAGGTGGTGCATGGCTGTCGTCAGCTCGTGCCGTAAGG
TGTTGGGTTAAGTCTCGCAACGAGCGCAACCCTCGTGTTTAGTTGCCACTATGAG
TTTGGAACCCTGAACAGACCGCCGGTGTTAAGCCGGAGGAAGGAGAGGATGAGG
CCAAGTCATCATGCCCCTTATGCCCTGGGCGACACACGTGCTACAATGGCGGGA
CAAAGGGTCGCGATCTCGCGAGGGTGAGCTAACTCCAAAAACCCGTCCTCAGTTC
GGATTGCAGGCTGCAACTCGCCTGCATGAAGCAGGAATCGCTAGTAATCGCCGGT
CAGCCATACGGCGGCGAATCCGTTCCCGGGCCTTGTACACACCGCCCGTCACACT
ATAGGAGCTGGCCAGGTTTGAAGTCATTACCCTTAACCGTAAGGAGGGGGATGCC
TAAGGCTAGGCTTGCGACTGGAGTGAAGTCGTAACAAGGTAGCCGTACTGGAAG
GTGCGGCTGGATCACCTCCTTT

>161934:emu_db:49306 ["161934:ncbi:49306 ['EF534108.133833.135302_U Beta vulgaris 16S ribosomal RNA, chloroplast']"]

AGAGTTCGATCCTGGCTCAGGATGAACGCTGGCGGCATGCTTAACACATGCAAGT
CGGACGGGAAGTGGTGTTTCCAGTGGCGGACGGGTGAGTAACGCGTAAGAACCT
GCCCTTGGGAGGGGAACAACAGCTGGAAACGGCTGCTAATACCCCGTAGGCTGA
GAAGCAAAAGGAGGAATCCGCCCGAGGAGGGGCTCGCGTCTGATTAGCTAGTTG
GTGAGGCAATGGCTTACCAAGGCGATGATCAGTAGCTGGTCCGAGAGGATGATC
AGCCACACTGGGACTGAGACACGGCCCAGACTCCTACGGGAGGCAGCAGTGGGG
AATTTTCCGCAATGGGCGAAAGCCTGACGGAGCAATGCCGCGTGGAGGTCGAAG
GCCCACGGGTCGTGAACTTCTTTTCCCGGAGAAGAAGCAATGACGGTATCCGGGG
AATAAGCATCGGCTAACTCTGTGCCAGCAGCCGCGGTAAGACAGAGGATGCAAG
CGTTATCCGGAATGATTGGGCGTAAAGCGTCTGTAGGTGGCTTTTTAAGTCCGCC
GTCAAATCCCAGGGCTCAACCCTGGACAGGCGGTGGAAACTATCAAGCTGGAGT
ACGGTAGGGGCAGAGGGAATTTCCGGTGGAGCGGTGAAATGCGTAGAGATCGGA
AAGAACACCAACGGCGAAAGCACTCTGCTGGGCCGACACTGACACTGAGAGACG
AAAGCTAGGGGAGCGAATGGGATTAGATACCCCAGTAGTCCTAGCCGTAAACGA
TGGATACTAGGCGCTGTGCGTATCGACCCGTGCAGTGTTGTAGCTAACGCGTTAA
GTATCCCGCCTGGGGAGTACGTTCGCAAGAATGAAACTCAAAGGAATTGACGGG
GGCCCGCACAAGCGGTGGAGCATGTGGTTTAATTCGATGCAAAGCGAAGAACCT
TACCAGGGCTTGACATGCCGCGAATCCTCTTGAAAGAGAGGGGTGCCTTCGGGAA
CGCGGATACAGGTGGTGCATGGCTGTCGTCAGCTCGTGCCGTAAGGTGTTGGGTT
AAGTCCCGCAACGAGCGCAACCCTCGTGTTTAGTTGCCAACGTTGAGTTTGGAAC
CCTGAACAGACTGCCGGTGATAAGCCGGAGGAAGGTGAGGATGACGTCAAGTCA
TCATGCCCCTTACGCCCTGGGCGACACACGTGCTACAATGGCCGGGACAAAGGGT
CGCGATCCCGCGAGGGTGAGCTAACCCCAAAAACCCGTCCTCAGTTCGGATTGCA
GGCTGCAACTCGCCTGCATGAAGCCGGAATCGCTAGTAATCGCCGGTCAGCCATA
CGGCGGTGAATTCGTTCCCGGGCCTTGTACACACCGCCCGTCACACTATGGGAGC
TGGCCATGCCCGAAGTCGTTACCTTAACCGCAAGGAGGGGGATGCCGAAGGCAG
GGCTAGTGACTGGAGTGAAGTCGTAACAAGGTAGCCGTACTGGAAGGTGCGGCT
G

>161934:emu_db:49307 ["161934:ncbi:49307 ['EF534108.97450.98919_U Beta vulgaris 16S ribosomal RNA, chloroplast']"]

AGAGTTCGATCCTGGCTCAGGATGAACGCTGGCGGCATGCTTAACACATGCAAGT
CGGACGGGAAGTGGTGTTTCCAGTGGCGGACGGGTGAGTAACGCGTAAGAACCT
GCCCTTGGGAGGGGAACAACAGCTGGAAACGGCTGCTAATACCCCGTAGGCTGA
GAAGCAAAAGGAGGAATCCGCCCGAGGAGGGGCTCGCGTCTGATTAGCTAGTTG
GTGAGGCAATGGCTTACCAAGGCGATGATCAGTAGCTGGTCCGAGAGGATGATC
AGCCACACTGGGACTGAGACACGGCCCAGACTCCTACGGGAGGCAGCAGTGGGG
AATTTTCCGCAATGGGCGAAAGCCTGACGGAGCAATGCCGCGTGGAGGTCGAAG
GCCCACGGGTCGTGAACTTCTTTTCCCGGAGAAGAAGCAATGACGGTATCCGGGG
AATAAGCATCGGCTAACTCTGTGCCAGCAGCCGCGGTAAGACAGAGGATGCAAG
CGTTATCCGGAATGATTGGGCGTAAAGCGTCTGTAGGTGGCTTTTTAAGTCCGCC
GTCAAATCCCAGGGCTCAACCCTGGACAGGCGGTGGAAACTATCAAGCTGGAGT
ACGGTAGGGGCAGAGGGAATTTCCGGTGGAGCGGTGAAATGCGTAGAGATCGGA
AAGAACACCAACGGCGAAAGCACTCTGCTGGGCCGACACTGACACTGAGAGACG
AAAGCTAGGGGAGCGAATGGGATTAGATACCCCAGTAGTCCTAGCCGTAAACGA
TGGATACTAGGCGCTGTGCGTATCGACCCGTGCAGTGTTGTAGCTAACGCGTTAA
GTATCCCGCCTGGGGAGTACGTTCGCAAGAATGAAACTCAAAGGAATTGACGGG
GGCCCGCACAAGCGGTGGAGCATGTGGTTTAATTCGATGCAAAGCGAAGAACCT
TACCAGGGCTTGACATGCCGCGAATCCTCTTGAAAGAGAGGGGTGCCTTCGGGAA
CGCGGATACAGGTGGTGCATGGCTGTCGTCAGCTCGTGCCGTAAGGTGTTGGGTT
AAGTCCCGCAACGAGCGCAACCCTCGTGTTTAGTTGCCAACGTTGAGTTTGGAAC
CCTGAACAGACTGCCGGTGATAAGCCGGAGGAAGGTGAGGATGACGTCAAGTCA
TCATGCCCCTTACGCCCTGGGCGACACACGTGCTACAATGGCCGGGACAAAGGGT
CGCGATCCCGCGAGGGTGAGCTAACCCCAAAAACCCGTCCTCAGTTCGGATTGCA
GGCTGCAACTCGCCTGCATGAAGCCGGAATCGCTAGTAATCGCCGGTCAGCCATA
CGGCGGTGAATTCGTTCCCGGGCCTTGTACACACCGCCCGTCACACTATGGGAGC
TGGCCATGCCCGAAGTCGTTACCTTAACCGCAAGGAGGGGGATGCCGAAGGCAG
GGCTAGTGACTGGAGTGAAGTCGTAACAAGGTAGCCGTACTGGAAGGTGCGGCT
G

>4072:emu_db:49308 ["4072:ncbi:49308 ['NC_018552.103639.105138 Capsicum annuum 16S ribosomal RNA, chloroplast']"]

TCTCATGGAGAGTTCGATCCTGGCTCAGGATGAACGCTGGCGGCATGCTTAACAC
ATGCAAGTCGGACGGGAAACACGGGAAACGGTGTTTCCAGTGGCGGACGGGTGA
GTAACGCGTAAGAACCTGCCCTTGGGAGGGGAACAACAGCTGGAAACGGCTGCT
AATACCCCGTAGGCTGAGGAGCAAAAGGAGGAATCCGCCCGAGGAGGGGCTCGC
GTCTGATTAGCTAGTTGGTGAGGCAATAGCTTACCAAGGCGATGATCAGTAGCTG
GTCCGAGAGGATGATCAGCCACACTGGGACTGAGACACGGCCCAGACTCCTACG
GGAGGCAGCAGTGGGGAATTTTCCGCAATGGGCGAAAGCCTGACGGAGCAATGC
CGCGTGGAGGTAGAAGGCCCACGGGTCGTGAACTTCTTTTCCCGGAGAAGAAGC
AATGACGGTATCTGGGGAATAAGCATCGGCTAACTCTGTGCCAGCAGCCGCGGTA
ATACAGAGGATGCAAGCGTTATCCGGAATGATTGGGCGTAAAGCGTCTGTAGGT
GGCTTTTTAAGTCCGCCGTCAAATCCCAGGGCTCAACCCTGGACAGGCGGTGGAA
ACTACCAAGCTGGAGTACGGTAGGGGCAGAGGGAATTTCCGGTGGAGCGGTGAA
ATGCGTAGAGATCGGAAAGAACACCAACGGCGAAAGCACTCTGCTGGGCCGACA
CTGACACTGAGAGACGAAAGCTAGGGGAGCGAATGGGATTAGATACCCCAGTAG
TCCTAGCCGTAAACGATGGATACTAGGCGCTGTGCGTATCGACCCGTGCAGTGCT
GTAGCTAACGCGTTAAGTATCCCGCCTGGGGAGTACGTTCGCAAGAATGAAACTC
AAAGGAATTGACGGGGGCCCGCACAAGCGGTGGAGCATGTGGTTTAATTCGATG
CAAAGCGAAGAACCTTACCAGGGCTTGACATGCCGCGAATCCTCTTGAAAGAGA
GGGGTGCCTTCGGGAACGCGGACACAGGTGGTGCATGGCTGTCGTCAGCTCGTGC

CGTAAGGTGTTGGGTTAAGTCCCGCAACGAGCGCAACCCTCGTGTTTAGTTGCCA
TCGTTGAGTTTGGAACCCTGAACAGACTGCCGGTGATAAGCCGGAGGAAGGTGA
GGATGACGTCAAGTCATCATGCCCCTTATGCCCTGGGCGACACACGTGCTACAAT
GGCCGGGACAAAGGGTCGCGATCCCGCGAGGGTGAGCTAACCCCAAAAACCCGT
CCTCAGTTCGGATTGCAGGCTGCAACTCGCCTGCATGAAGCCGGAATCGCTAGTA
ATCGCCGGTCAGCCATACGGCGGTGAATTCGTTCCCGGGCCTTGTACACACCGCC
CGTCACACTATGGGAGCTGGCCATGCCCGAAGTCGTTACCTTAACCGCAAGGAGG
GGGATGCCGAAGGCAGGGCTAGTGACTGGAGTGAAGTCGTAACAAGGTAGCCGT
ACTGGAAGGTGCGGCTGGATCACCTCCTTT

>4072:emu_db:49309 ["4072:ncbi:49309 ['NC_018552.139010.140509 Capsicum annuum 16S ribosomal RNA, chloroplast']"]

TCTCATGGAGAGTTCGATCCTGGCTCAGGATGAACGCTGGCGGCATGCTTAACAC
ATGCAAGTCGGACGGGAAACACGGGAAACGGTGTTTCCAGTGGCGGACGGGTGA
GTAACGCGTAAGAACCTGCCCTTGGGAGGGGAACAACAGCTGGAAACGGCTGCT
AATACCCCGTAGGCTGAGGAGCAAAAGGAGGAATCCGCCCGAGGAGGGGCTCGC
GTCTGATTAGCTAGTTGGTGAGGCAATAGCTTACCAAGGCGATGATCAGTAGCTG
GTCCGAGAGGATGATCAGCCACACTGGGACTGAGACACGGCCCAGACTCCTACG
GGAGGCAGCAGTGGGGAATTTTCCGCAATGGGCGAAAGCCTGACGGAGCAATGC
CGCGTGGAGGTAGAAGGCCCACGGGTCGTGAACTTCTTTTCCCGGAGAAGAAGC
AATGACGGTATCTGGGGAATAAGCATCGGCTAACTCTGTGCCAGCAGCCGCGGTA
ATACAGAGGATGCAAGCGTTATCCGGAATGATTGGGCGTAAAGCGTCTGTAGGT
GGCTTTTTAAGTCCGCCGTCAAATCCCAGGGCTCAACCCTGGACAGGCGGTGGAA
ACTACCAAGCTGGAGTACGGTAGGGGCAGAGGGAATTTCCGGTGGAGCGGTGAA
ATGCGTAGAGATCGGAAAGAACACCAACGGCGAAAGCACTCTGCTGGGCCGACA
CTGACACTGAGAGACGAAAGCTAGGGGAGCGAATGGGATTAGATACCCCAGTAG
TCCTAGCCGTAAACGATGGATACTAGGCGCTGTGCGTATCGACCCGTGCAGTGCT
GTAGCTAACGCGTTAAGTATCCCGCCTGGGGAGTACGTTCGCAAGAATGAAACTC
AAAGGAATTGACGGGGGCCCGCACAAGCGGTGGAGCATGTGGTTTAATTCGATG
CAAAGCGAAGAACCTTACCAGGGCTTGACATGCCGCGAATCCTCTTGAAAGAGA
GGGGTGCCTTCGGGAACGCGGACACAGGTGGTGCATGGCTGTCGTCAGCTCGTGC
CGTAAGGTGTTGGGTTAAGTCCCGCAACGAGCGCAACCCTCGTGTTTAGTTGCCA
TCGTTGAGTTTGGAACCCTGAACAGACTGCCGGTGATAAGCCGGAGGAAGGTGA
GGATGACGTCAAGTCATCATGCCCCTTATGCCCTGGGCGACACACGTGCTACAAT
GGCCGGGACAAAGGGTCGCGATCCCGCGAGGGTGAGCTAACCCCAAAAACCCGT
CCTCAGTTCGGATTGCAGGCTGCAACTCGCCTGCATGAAGCCGGAATCGCTAGTA
ATCGCCGGTCAGCCATACGGCGGTGAATTCGTTCCCGGGCCTTGTACACACCGCC
CGTCACACTATGGGAGCTGGCCATGCCCGAAGTCGTTACCTTAACCGCAAGGAGG
GGGATGCCGAAGGCAGGGCTAGTGACTGGAGTGAAGTCGTAACAAGGTAGCCGT
ACTGGAAGGTGCGGCTGGATCACCTCCTTT

>4072:emu_db:49310 ["4072:ncbi:49310 ['HM753597.1.1386_U Capsicum annuum 16S ribosomal RNA, chloroplast']"]

GCTGGGCGCTGCTTACACATGCAAGTCGGACGGGAAACACGGGAAACGGTGTTT
CCAGTGGCGGACGGGTGAGTAACGCGTAAGAACCTGCCCTTGGGAGGGGAACAA
CAGCTGGAAACGGCTGCTAATACCCCGTAGGCTGAGGAGCAAAAGGAGGAATCC
GCCCGAGGAGGGGCTCGCGTCTGATTAGCTAGTTGGTGAGGCAATAGCTTACCAA
GGCGATGATCAGTAGCTGGTCCGAGAGGATGATCAGCCACACTGGGACTGAGAC
ACGGCCCAGACTCCTACGGGAGGCAGCATTGGGGAATTTTCCGCAATGGGCGAA

AGCCTGACGGAGCAATGCCGCGTGGAGGTAGAAGGCCCACGGGTCGTGAACTTC
TTTTCCCGGAGAAGAAGCAATGACGGTATCTGGGGAATAAGCATCGGCTAACTCT
GTGCCAGCAGCCGCGGTATACAGAGGATGCAAGCGTATCCGGAATGTTGGGCGT
AAGCGTCTGTAGGTGGCTTTTTAAGTCCGCCGTCAAATCCCAGGGCTCAACCCTG
GACAGGCGTGGAAACTACCAAGCTGGAGTACGGTAGGGGCAGAGGGAATTTCCG
GTGGAGCGGTGAAATGCGTAGAGATCGGAAAGAACACCAACGGCGAAAGCACTC
TGCTGGGCCGACACTGACACTGAGAGACGAAAGCTAGGGGAGCGAATGGGATTA
GATACCCCAGTAGTCCTAGCCGTAAACGATGGATACTAGGCGCTGTGCGTATCGA
CCCGTGCAGTGCTGTAGCTAACGCGTTAAGTATCCCGCCTGGGGAGTACGTTCGC
AAGAATGAAACTCAAAGGAATTGACGGGGGCCCGCACAAGCGGTGGAGCATGTG
GTTTAATTCGATGCAAAGCGAAGAACCTTACCAGGGCTTGACATGCCGCGAATCC
TCTTGAAAGAGAGGGGTGCCTTCGGGAACGCGGACACAGGTGGTGCATGGCTGT
CGTCAGCTCGTGCCGTAAGGTGTTGGGTTAAGTCCCGCAACGAGCGCAACCCTCG
TGTTTAGTTGCCATCGTTGAGTTTGGAACCCTGAACAGACTGCCGGTGATAAGCC
GGAGGAAGGTGAGGATGACGTCAAGTCATCATGCCCCTTATGCCCTGGGCGACA
CACGTGCTACAATGGCCGGGACAAAGGGTCGCGATCCCGCGAGGGTGAGCTAAC
CCCAAAAACCCGTCCTCAGTTCGGATTGCAGGCTGCAACTCGCCTGCATGAAGCC
GGAATCGCTAGTAATCGCCGGTCAGCCATACGGCGGTGAATTCGTTCCCGGGCCT
TGTACACACCGCCCGTCACACTATGGGAGCTGGCCATGCCCGAAGTCGTTACCTT
AACCGCAAGGAGGAGGATGCCGAAG

>4072:emu_db:49311 ["4072:ncbi:49311 ['JX270811.139010.140509_U Capsicum annuum 16S ribosomal RNA, chloroplast']"]

TCTCATGGAGAGTTCGATCCTGGCTCAGGATGAACGCTGGCGGCATGCTTAACAC
ATGCAAGTCGGACGGGAAACACGGGAAACGGTGTTTCCAGTGGCGGACGGGTGA
GTAACGCGTAAGAACCTGCCCTTGGGAGGGGAACAACAGCTGGAAACGGCTGCT
AATACCCCGTAGGCTGAGGAGCAAAAGGAGGAATCCGCCCGAGGAGGGGCTCGC
GTCTGATTAGCTAGTTGGTGAGGCAATAGCTTACCAAGGCGATGATCAGTAGCTG
GTCCGAGAGGATGATCAGCCACACTGGGACTGAGACACGGCCCAGACTCCTACG
GGAGGCAGCAGTGGGGAATTTTCCGCAATGGGCGAAAGCCTGACGGAGCAATGC
CGCGTGGAGGTAGAAGGCCCACGGGTCGTGAACTTCTTTTCCCGGAGAAGAAGC
AATGACGGTATCTGGGGAATAAGCATCGGCTAACTCTGTGCCAGCAGCCGCGGTA
ATACAGAGGATGCAAGCGTTATCCGGAATGATTGGGCGTAAAGCGTCTGTAGGT
GGCTTTTTAAGTCCGCCGTCAAATCCCAGGGCTCAACCCTGGACAGGCGGTGGAA
ACTACCAAGCTGGAGTACGGTAGGGGCAGAGGGAATTTCCGGTGGAGCGGTGAA
ATGCGTAGAGATCGGAAAGAACACCAACGGCGAAAGCACTCTGCTGGGCCGACA
CTGACACTGAGAGACGAAAGCTAGGGGAGCGAATGGGATTAGATACCCCAGTAG
TCCTAGCCGTAAACGATGGATACTAGGCGCTGTGCGTATCGACCCGTGCAGTGCT
GTAGCTAACGCGTTAAGTATCCCGCCTGGGGAGTACGTTCGCAAGAATGAAACTC
AAAGGAATTGACGGGGGCCCGCACAAGCGGTGGAGCATGTGGTTTAATTCGATG
CAAAGCGAAGAACCTTACCAGGGCTTGACATGCCGCGAATCCTCTTGAAAGAGA
GGGGTGCCTTCGGGAACGCGGACACAGGTGGTGCATGGCTGTCGTCAGCTCGTGC
CGTAAGGTGTTGGGTTAAGTCCCGCAACGAGCGCAACCCTCGTGTTTAGTTGCCA
TCGTTGAGTTTGGAACCCTGAACAGACTGCCGGTGATAAGCCGGAGGAAGGTGA
GGATGACGTCAAGTCATCATGCCCCTTATGCCCTGGGCGACACACGTGCTACAAT
GGCCGGGACAAAGGGTCGCGATCCCGCGAGGGTGAGCTAACCCCAAAAACCCGT
CCTCAGTTCGGATTGCAGGCTGCAACTCGCCTGCATGAAGCCGGAATCGCTAGTA
ATCGCCGGTCAGCCATACGGCGGTGAATTCGTTCCCGGGCCTTGTACACACCGCC
CGTCACACTATGGGAGCTGGCCATGCCCGAAGTCGTTACCTTAACCGCAAGGAGG

GGGATGCCGAAGGCAGGGCTAGTGACTGGAGTGAAGTCGTAACAAGGTAGCCGT
ACTGGAAGGTGCGGCTGGATCACCTCCTTT

>4072:emu_db:49312 ["4072:ncbi:49312 ['JX270811.103639.105138_U Capsicum annuum 16S ribosomal RNA, chloroplast']"]

TCTCATGGAGAGTTCGATCCTGGCTCAGGATGAACGCTGGCGGCATGCTTAACAC
ATGCAAGTCGGACGGAAACACGGAAACGGTGTTTCCAGTGGCGGACGGGTGA
GTAACGCGTAAGAACCTGCCCTTGGGAGGGGAACAACAGCTGGAAACGGCTGCT
AATACCCCGTAGGCTGAGGAGCAAAAGGAGGAATCCGCCCGAGGAGGGGCTCGC
GTCTGATTAGCTAGTTGGTGAGGCAATAGCTTACCAAGGCGATGATCAGTAGCTG
GTCCGAGAGGATGATCAGCCACACTGGGACTGAGACACGGCCCAGACTCCTACG
GGAGGCAGCAGTGGGGAATTTTCCGCAATGGGCGAAAGCCTGACGGAGCAATGC
CGCGTGGAGGTAGAAGGCCCACGGGTCGTGAACTTCTTTTCCCGGAGAAGAAGC
AATGACGGTATCTGGGGAATAAGCATCGGCTAACTCTGTGCCAGCAGCCGCGGTA
ATACAGAGGATGCAAGCGTTATCCGGAATGATTGGGCGTAAAGCGTCTGTAGGT
GGCTTTTTAAGTCCGCCGTCAAATCCCAGGGCTCAACCCTGGACAGGCGGTGGAA
ACTACCAAGCTGGAGTACGGTAGGGGCAGAGGGAATTTCCGGTGGAGCGGTGAA
ATGCGTAGAGATCGGAAAGAACACCAACGGCGAAAGCACTCTGCTGGGCCGACA
CTGACACTGAGAGACGAAAGCTAGGGGAGCGAATGGGATTAGATACCCCAGTAG
TCCTAGCCGTAAACGATGGATACTAGGCGCTGTGCGTATCGACCCGTGCAGTGCT
GTAGCTAACGCGTTAAGTATCCCGCCTGGGGAGTACGTTCGCAAGAATGAAACTC
AAAGGAATTGACGGGGGCCCGCACAAGCGGTGGAGCATGTGGTTTAATTCGATG
CAAAGCGAAGAACCTTACCAGGGCTTGACATGCCGCGAATCCTCTTGAAAGAGA
GGGGTGCCTTCGGGAACGCGGACACAGGTGGTGCATGGCTGTCGTCAGCTCGTGC
CGTAAGGTGTTGGGTTAAGTCCCGCAACGAGCGCAACCCTCGTGTTTAGTTGCCA
TCGTTGAGTTTGGAACCCTGAACAGACTGCCGGTGATAAGCCGGAGGAAGGTGA
GGATGACGTCAAGTCATCATGCCCCTTATGCCCTGGGCGACACACGTGCTACAAT
GGCCGGGACAAAGGGTCGCGATCCCGCGAGGGTGAGCTAACCCCAAAAACCCGT
CCTCAGTTCGGATTGCAGGCTGCAACTCGCCTGCATGAAGCCGGAATCGCTAGTA
ATCGCCGGTCAGCCATACGGCGGTGAATTCGTTCCCGGGCCTTGTACACACCGCC
CGTCACACTATGGGAGCTGGCCATGCCCGAAGTCGTTACCTTAACCGCAAGGAGG
GGGATGCCGAAGGCAGGGCTAGTGACTGGAGTGAAGTCGTAACAAGGTAGCCGT
ACTGGAAGGTGCGGCTGGATCACCTCCTTT

>4039:emu_db:49313 ["4039:ncbi:49313 ['DQ898156.137616.139105_U Daucus carota 16S ribosomal RNA, chloroplast']"]

CTCATGGAGAGTTCGATCCTGGCTCAGGATGAACGCTGGCGGCATGCTTAACACA
TGCAAGTCGGACGGGAAGTGGTGTTTCCAGTGGCGGACGGGTGAGTAACGCGTA
AGAACCTGCCCTTGGGAGGGGAACAACAGCTGGAAACGGCTGCTAATACCCCGT
AGGCTGAGGAGCAAAAGGAGGAATCCGCCCGAGGAGGGGCTCGCGTCTGATTAG
CTAGTTGGTGAGGCAATAGCTTACCAAGGCGATGATCAGTAGCTGGTCCGAGAG
GATGATCAGCCACACTGGGACTGAGACACGGCCCAGACTCCTACGGGAGGCAGC
AGTGGGGAATTTTCCGCAATGGGCGAAAGCCTGACGGAGCAATGCCGCGTGGAG
GTAGAAGGCCCACGGGTCGTGAACTTCTTTTCCCGGAGAAGAAGCAATGACGGT
ATCTGGGGAATAAGCATCGGCTAACTCTGTGCCAGCAGCCGCGGTAATACAGAG
GATGCAAGCGTTATCCGGAATTATTGGGCGTAAAGCGTCTGTAGGTGGCTTTTTA
AGTCCGCCGTCAAATCCCAGGGCTCAACCCTGGACAGGCGGTGGAAACTACCAA
GCTGGAGTACGGTAGGGGCAGAGGGAATTTCCGGTGGAGCGGTGAAATGCGTAG
AGATCGGAAAGAACACCAACGGCGAAAGCACTCTGCTGGGCCGACACTGACACT

GAGAGACGAAAGCTAGGGTAGCGAATGGGATTAGATACCCCAGTAGTCCTAGCC
GTAAACGATGGATACTAGGCGCTGTGCGTATCGACCCGTGCAGTGCTGTAGCTAA
CGCGTTAAGTATCCCGCCTGGGGAGTACGTTCGCAAGAATGAAACTCAAAGGAA
TTGACGGGGGCCCGCACAAGCGGTGGAGCATGTGGTTTAATTCGATGCAAAGCG
AAGAACCTTACCAGGGCTTGACATGCCGCGAATCCTCTTGAAAGAGAGGGGTGC
CTTCGGGAACGCGGACACAGGTGGTGCATGGCTGTCGTCAGCTCGTGCCGTAAGG
TGTTGGGTTAAGTCCCGCAACGAGCGCAACCCTCGTGTTTAGTTGCCATCATTGA
GTTTGGAACCCTGAACAGACTGCCGGTGATAAGCCGGAGGAAGGTGAGGATGAC
GTCAAGTCATCATGCCCCTTATGCCCTGGGCGACACACGTGCTACAATGGCCGGG
ACAAAGGGTCGCGATCCCGCGAGGGTGAGCTAACTCCAAAAACCCGTCCTCAGTT
CGGATTGCAGGCTGCAACTCGCCTGCATGAAGCCGGAATCGCTAGTAATCGCCGG
TCAGCCATACGGCGGTGAATTCGTTCCCGGGCCTTGTACACACCGCCCGTCACAC
TATGGGAGCTGGCCATGCCCGAAGTCGTTACCTTAACCGCAAGGAGGGGGATGC
CGAAGGCAGGGCTAGTGACTGGAGTGAAGTCGTAACAAGGTAGCCGTACTGGAA
GGTGCGGCTGGATCACCTCCTTT

>4039:emu_db:49314 ["4039:ncbi:49314 ['DQ898156.101048.102537_U Daucus carota 16S ribosomal RNA, chloroplast']"]

TCTCATGGAGAGTTCGATCCTGGCTCAGGATGAACGCTGGCGGCATGCTTAACAC
ATGCAAGTCGGACGGGAAGTGGTGTTTCCAGTGGCGGACGGGTGAGTAACGCGT
AAGAACCTGCCCTTGGGAGGGGAACAACAGCTGGAAACGGCTGCTAATACCCCG
TAGGCTGAGGAGCAAAAGGAGGAATCCGCCCGAGGAGGGGCTCGCGTCTGATTA
GCTAGTTGGTGAGGCAATAGCTTACCAAGGCGATGATCAGTAGCTGGTCCGAGA
GGATGATCAGCCACACTGGGACTGAGACACGGCCCAGACTCCTACGGGAGGCAG
CAGTGGGGAATTTTCCGCAATGGGCGAAAGCCTGACGGAGCAATGCCGCGTGGA
GGTAGAAGGCCCACGGGTCGTGAACTTCTTTTCCCGGAGAAGAAGCAATGACGG
TATCTGGGGAATAAGCATCGGCTAACTCTGTGCCAGCAGCCGCGGTAATACAGAG
GATGCAAGCGTTATCCGGAATTATTGGGCGTAAAGCGTCTGTAGGTGGCTTTTTA
AGTCCGCCGTCAAATCCCAGGGCTCAACCCTGGACAGGCGGTGGAAACTACCAA
GCTGGAGTACGGTAGGGGCAGAGGGAATTTCCGGTGGAGCGGTGAAATGCGTAG
AGATCGGAAAGAACACCAACGGCGAAAGCACTCTGCTGGGCCGACACTGACACT
GAGAGACGAAAGCTAGGGTAGCGAATGGGATTAGATACCCCAGTAGTCCTAGCC
GTAAACGATGGATACTAGGCGCTGTGCGTATCGACCCGTGCAGTGCTGTAGCTAA
CGCGTTAAGTATCCCGCCTGGGGAGTACGTTCGCAAGAATGAAACTCAAAGGAA
TTGACGGGGGCCCGCACAAGCGGTGGAGCATGTGGTTTAATTCGATGCAAAGCG
AAGAACCTTACCAGGGCTTGACATGCCGCGAATCCTCTTGAAAGAGAGGGGTGC
CTTCGGGAACGCGGACACAGGTGGTGCATGGCTGTCGTCAGCTCGTGCCGTAAGG
TGTTGGGTTAAGTCCCGCAACGAGCGCAACCCTCGTGTTTAGTTGCCATCATTGA
GTTTGGAACCCTGAACAGACTGCCGGTGATAAGCCGGAGGAAGGTGAGGATGAC
GTCAAGTCATCATGCCCCTTATGCCCTGGGCGACACACGTGCTACAATGGCCGGG
ACAAAGGGTCGCGATCCCGCGAGGGTGAGCTAACTCCAAAAACCCGTCCTCAGTT
CGGATTGCAGGCTGCAACTCGCCTGCATGAAGCCGGAATCGCTAGTAATCGCCGG
TCAGCCATACGGCGGTGAATTCGTTCCCGGGCCTTGTACACACCGCCCGTCACAC
TATGGGAGCTGGCCATGCCCGAAGTCGTTACCTTAACCGCAAGGAGGGGGATGC
CGAAGGCAGGGCTAGTGACTGGAGTGAAGTCGTAACAAGGTAGCCGTACTGGAA
GGTGCGGCTGGATCACCTCCTT

>4039:emu_db:49315 ["4039:ncbi:49315 ['X73670.301.1784_U Daucus carota 16S ribosomal RNA, chloroplast']"]

TCTCATGGAGAGTTCGATCCTGGCTCAGGATGAACGCTGGCGGCATGGAAAACAC
ATGCAAGTCGGACGGAAGTGGTGTTTCCAGTGGCGGACGGACTGTAACGCGT
AAGAACCTGCCCTTGGGTGGGGAACAACAGCTGGAAACGGCTGCTAATACCCCG
TAGGCTGAGGAGCAAAAGGAGGAATCCGCCCGAGGGAGGGGCTCGCGTCTGATA
GCTAGTTGGTGAGGCAATAGCTTACCAAGGCGATGATCAGTAGCTGGTCCGAGA
GGATGATCAGCCACACTGGGACTGAGACACGGCCCAGACTCCTACGGGAGGCAG
CAGTGGGGAATTTTCCGCAATGGGCGAAAGCGACGGAGCAATGCCGCGTGGAGG
TAGAAGGCCCACGGGTCGTGAACTTCTTTTCCCGGAGAAGAAGCAATGACGGTAT
CTGGGGAATAAGCATCGGCTAACTCTGTGCCAGCAGCCGCGGTAATACAGAGGA
TGCAAGCGTTATCCGGAATGATTGGGCGTAAAGCGTCTGTAGGTGGCTTTTTAAG
TCCGCCGTCAAATCCCAGGGCTCAACCCTGGACAGGCGGTGGAAACTACCAAGCT
GGAGTACGGTAGGGGCAGAGGGAATTTCCGGTGGAGCGGTGAAATGCGTAGAGA
TCGGAAAGAACACCAACGGCGAAAGCACTCTGCTGGGCCGACATTGACACTGAG
AGACGAAAGCTAGGGGAGCGAATGGGATTAAATACCCCATTAGTCCTAGCCGTA
AACGATGGATACTAGGCGCTGTGCGATCGACCCGTGCAGTGCTGTAACTACCGCG
TTAAGTATCCCGCCTGGGGAGTACGTTCGCAAGAATGAAACTCAAAGGAATTGAC
GGGGGCGCGCACAATCGGTGGAGCATGTGGTTTAATTCGATGCAAAGCGAAGAA
CCTTACCATGGCTTGACATGCCGCGAATCCTCTTGAAAGAGAGGGGTGCCTTCGG
GAACGCGGACACAGGTGGTGAATGGCTGTCGTCAGCTCGTGCCGTAAGGTGTTGG
GTTAAGTCCCGCAACGAGCGCAACCCTCGTGTTTAGTTGCCATCGTTGAGTTTGG
AACCCTGAACAGACTGCCGGTGATAAGCCGGAGGAAGGTGAGGATGACGTCAAG
TCATCATGCCCCTTATGCCCTTGGCGACACTCGTGCTACAATGGCCGGGACAAAG
GGTTGCGATCCCGCGAGGGTGAGCTAACCCCAAAAACCCGTCCTCAGTTGGGATT
GCAGGCTGCAACTCGCCTGCATGAAGCCGGAATCGCTAGTAATCGCCGGTCAGCC
ATACGGCGGTGAATTCGGTACCGGGCCTTGTACACACCGCCCGTCACACTATGGG
AGCTGGCCATGCCCGAAGTCGTTACCTTAACCGCAAGGGGGGGATGCCGAAGCA
GGGCTAGTGACTGGAGTGAAGTCGTAACAAGGTAGCCGTACTGGAAGGTGCGGC
TGGATCACCTCCT

>4039:emu_db:49316 ["4039:ncbi:49316 ['NC_008325.137616.139105_U Daucus carota 16S ribosomal RNA, chloroplast']"]

CTCATGGAGAGTTCGATCCTGGCTCAGGATGAACGCTGGCGGCATGCTTAACACA
TGCAAGTCGGACGGGAAGTGGTGTTTCCAGTGGCGGACGGGTGAGTAACGCGTA
AGAACCTGCCCTTGGGAGGGGAACAACAGCTGGAAACGGCTGCTAATACCCCGT
AGGCTGAGGAGCAAAAGGAGGAATCCGCCCGAGGAGGGGCTCGCGTCTGATTAG
CTAGTTGGTGAGGCAATAGCTTACCAAGGCGATGATCAGTAGCTGGTCCGAGAG
GATGATCAGCCACACTGGGACTGAGACACGGCCCAGACTCCTACGGGAGGCAGC
AGTGGGGAATTTTCCGCAATGGGCGAAAGCCTGACGGAGCAATGCCGCGTGGAG
GTAGAAGGCCCACGGGTCGTGAACTTCTTTTCCCGGAGAAGAAGCAATGACGGT
ATCTGGGGAATAAGCATCGGCTAACTCTGTGCCAGCAGCCGCGGTAATACAGAG
GATGCAAGCGTTATCCGGAATTATTGGGCGTAAAGCGTCTGTAGGTGGCTTTTTA
AGTCCGCCGTCAAATCCCAGGGCTCAACCCTGGACAGGCGGTGGAAACTACCAA
GCTGGAGTACGGTAGGGGCAGAGGGAATTTCCGGTGGAGCGGTGAAATGCGTAG
AGATCGGAAAGAACACCAACGGCGAAAGCACTCTGCTGGGCCGACACTGACACT
GAGAGACGAAAGCTAGGGTAGCGAATGGGATTAGATACCCCAGTAGTCCTAGCC
GTAAACGATGGATACTAGGCGCTGTGCGTATCGACCCGTGCAGTGCTGTAGCTAA
CGCGTTAAGTATCCCGCCTGGGGAGTACGTTCGCAAGAATGAAACTCAAAGGAA
TTGACGGGGGCCCGCACAAGCGGTGGAGCATGTGGTTTAATTCGATGCAAAGCG
AAGAACCTTACCAGGGCTTGACATGCCGCGAATCCTCTTGAAAGAGAGGGGTGC
CTTCGGGAACGCGGACACAGGTGGTGCATGGCTGTCGTCAGCTCGTGCCGTAAGG

TGTTGGGTTAAGTCCCGCAACGAGCGCAACCCTCGTGTTTAGTTGCCATCATTGA
GTTTGGAACCCTGAACAGACTGCCGGTGATAAGCCGGAGGAAGGTGAGGATGAC
GTCAAGTCATCATGCCCCTTATGCCCTGGGCGACACACGTGCTACAATGGCCGGG
ACAAAGGGTCGCGATCCCGCGAGGGTGAGCTAACTCCAAAAACCCGTCCTCAGTT
CGGATTGCAGGCTGCAACTCGCCTGCATGAAGCCGGAATCGCTAGTAATCGCCGG
TCAGCCATACGGCGGTGAATTCGTTCCCGGGCCTTGTACACACCGCCCGTCACAC
TATGGGAGCTGGCCATGCCCGAAGTCGTTACCTTAACCGCAAGGAGGGGGATGC
CGAAGGCAGGGCTAGTGACTGGAGTGAAGTCGTAACAAGGTAGCCGTACTGGAA
GGTGCGGCTGGATCACCTCCTTT

>4039:emu_db:49317 ["4039:ncbi:49317 ['NC_008325.101048.102537_U Daucus carota 16S ribosomal RNA, chloroplast']"]

TCTCATGGAGAGTTCGATCCTGGCTCAGGATGAACGCTGGCGGCATGCTTAACAC
ATGCAAGTCGGACGGGAAGTGGTGTTTCCAGTGGCGGACGGGTGAGTAACGCGT
AAGAACCTGCCCTTGGGAGGGGAACAACAGCTGGAAACGGCTGCTAATACCCCG
TAGGCTGAGGAGCAAAAGGAGGAATCCGCCCGAGGAGGGGCTCGCGTCTGATTA
GCTAGTTGGTGAGGCAATAGCTTACCAAGGCGATGATCAGTAGCTGGTCCGAGA
GGATGATCAGCCACACTGGGACTGAGACACGGCCCAGACTCCTACGGGAGGCAG
CAGTGGGGAATTTTCCGCAATGGGCGAAAGCCTGACGGAGCAATGCCGCGTGGA
GGTAGAAGGCCCACGGGTCGTGAACTTCTTTTCCCGGAGAAGAAGCAATGACGG
TATCTGGGGAATAAGCATCGGCTAACTCTGTGCCAGCAGCCGCGGTAATACAGAG
GATGCAAGCGTTATCCGGAATTATTGGGCGTAAAGCGTCTGTAGGTGGCTTTTTA
AGTCCGCCGTCAAATCCCAGGGCTCAACCCTGGACAGGCGGTGGAAACTACCAA
GCTGGAGTACGGTAGGGGCAGAGGGAATTTCCGGTGGAGCGGTGAAATGCGTAG
AGATCGGAAAGAACACCAACGGCGAAAGCACTCTGCTGGGCCGACACTGACACT
GAGAGACGAAAGCTAGGGTAGCGAATGGGATTAGATACCCCAGTAGTCCTAGCC
GTAAACGATGGATACTAGGCGCTGTGCGTATCGACCCGTGCAGTGCTGTAGCTAA
CGCGTTAAGTATCCCGCCTGGGGAGTACGTTCGCAAGAATGAAACTCAAAGGAA
TTGACGGGGGCCCGCACAAGCGGTGGAGCATGTGGTTTAATTCGATGCAAAGCG
AAGAACCTTACCAGGGCTTGACATGCCGCGAATCCTCTTGAAAGAGAGGGGTGC
CTTCGGGAACGCGGACACAGGTGGTGCATGGCTGTCGTCAGCTCGTGCCGTAAGG
TGTTGGGTTAAGTCCCGCAACGAGCGCAACCCTCGTGTTTAGTTGCCATCATTGA
GTTTGGAACCCTGAACAGACTGCCGGTGATAAGCCGGAGGAAGGTGAGGATGAC
GTCAAGTCATCATGCCCCTTATGCCCTGGGCGACACACGTGCTACAATGGCCGGG
ACAAAGGGTCGCGATCCCGCGAGGGTGAGCTAACTCCAAAAACCCGTCCTCAGTT
CGGATTGCAGGCTGCAACTCGCCTGCATGAAGCCGGAATCGCTAGTAATCGCCGG
TCAGCCATACGGCGGTGAATTCGTTCCCGGGCCTTGTACACACCGCCCGTCACAC
TATGGGAGCTGGCCATGCCCGAAGTCGTTACCTTAACCGCAAGGAGGGGGATGC
CGAAGGCAGGGCTAGTGACTGGAGTGAAGTCGTAACAAGGTAGCCGTACTGGAA
GGTGCGGCTGGATCACCTCCTT

>4513:emu_db:49318 ["4513:ncbi:49318 ['BACC01003966.4091.5560_U Horedeum vulgare 16S ribosomal RNA, chloroplast']"]

AGAGTTTGATCCTGGCTCAGGATGAACGCTGGCGGCATGCTTAACACATGCAAGT
CGAACGGGAAGTGGTGTTTCCAGTGGCGAACGGGTGAGTAACGCGTAAGAACCT
GCCCTTGGGAGGGGAACAACAACTGGAAACGGTTGCTAATACCCCGTAGGCTGA
GGAGCAAAAGGAGAAATCCGCCCAAGGAGGGGCTCGCGTCTGATTAGCTAGTTG
GTGAGGCAATAGCTTACCAAGGCGATGATCAGTAGCTGGTCCGAGAGGATGATC
AGCCACACTGGGACTGAGACACGGCCCAGACTCCTACGGGAGGCAGCAGTGGGG

AATTTTCCGCAATGGGCGAAAGCCTGACGGAGCAATGCCGCGTGGAGGTGGAAG
GCCTACGGGTCGTCAACTTCTTTTCTCGGAGAAGAAACAATGACGGTATCTGAGG
AATAAGCATCGGCTAACTCTGTGCCAGCAGCCGCGGTAAGACAGAGGATGCAAG
CGTTATCCGGAATGATTGGGCGTAAAGCGTCTGTAGGTGGCTTTTCAAGTCCGCC
GTCAAATCCCAGGGCTCAACCCTGGACAGGCGGTGGAAACTACCAAGCTGGAGT
ACGGTAGGGGCAGAGGGAATTTCCGGTGGAGCGGTGAAATGCATTGAGATCGGA
AAGAACACCAACGGCGAAAGCACTCTGCTGGGCCGACACTGACACTGAGAGACG
AAAGCTAGGGGAGCAAATGGGATTAGAGACCCCAGTAGTCCTAGCCGTAAACGA
TGGATACTAGGTGCTGTGCGACTCGACCCGTGCAGTGCTGTAGCTAACGCGTTAA
GTATCCCGCCTGGGGAGTACGTTCGCAAGAATGAAACTCAAAGGAATTGACGGG
GGCCCGCACAAGCGGTGGAGCATGTGGTTTAATTCGATGCAAAGCGAAGAACCT
TACCAGGGCTTGACATGCCGCGAATCCTCTTGAAAGAGAGGGGTGCCCTCGGGA
ACGCGGACACAGGTGGTGCATGGCTGTCGTCAGCTCGTGCCGTAAGGTGTTGGGT
TAAGTCTCGCAACGAGCGCAACCCTCGTGTTTAGTTGCCACTATGAGTTTGGAAC
CCTGAACAGACCGCCGGTGTTAAGCCGGAGGAAGGAGAGGATGAGGCCAAGTCA
TCATGCCCCTTATGCCCTGGGCGACACACGTGCTACAATGGGCGGGACAAAGGGT
CGCGATCTCGCGAGGGTGAGCTAACTCCAAAAACCCGTCCTCAGTTCGGATTGCA
GGCTGCAACTCGCCTGCATGAAGCAGGAATCGCTAGTAATCGCCGGTCAGCCATA
CGGCGGTGAATCCGTTCCCGGGCCTTGTACACACCGCCCGTCACACTATAGGAGC
TGGCCATGTTTGAAGTCATTACCCTTAACCGTAAGGAGGGGGATGCCTAAGGCTA
GGCTTGCGACTGGAGTGAAGTCGTAACAAGGTAGCCGTACTGGAAGGTGCGGCT
G

>4513:emu_db:49319 ["4513:ncbi:49319 ['EF115541.92685.94176_U Horedeum vulgare 16S ribosomal RNA, chloroplast']"]

ATCTCATGGAGAGTTTGATCCTGGCTCAGGATGAACGCTGGCGGCATGCTTAACA
CATGCAAGTCGAACGGGAAGTGGTGTTTCCAGTGGCGAACGGGTGAGTAACGCG
TAAGAACCTGCCCTTGGGAGGGGAACAACAACTGGAAACGGTTGCTAATACCCC
GTAGGCTGAGGAGCAAAAGGAGAAATCCGCCCAAGGAGGGGCTCGCGTCTGATT
AGCTAGTTGGTGAGGCAATAGCTTACCAAGGCGATGATCAGTAGCTGGTCCGAG
AGGATGATCAGCCACACTGGGACTGAGACACGGCCCAGACTCCTACGGGAGGCA
GCAGTGGGGAATTTTCCGCAATGGGCGAAAGCCTGACGGAGCAATGCCGCGTGG
AGGTGGAAGGCCTACGGGTCGTCAACTTCTTTTCTCGGAGAAGAAACAATGACGG
TATCTGAGGAATAAGCATCGGCTAACTCTGTGCCAGCAGCCGCGGTAAGACAGA
GGATGCAAGCGTTATCCGGAATGATTGGGCGTAAAGCGTCTGTAGGTGGCTTTTC
AAGTCCGCCGTCAAATCCCAGGGCTCAACCCTGGACAGGCGGTGGAAACTACCA
AGCTGGAGTACGGTAGGGGCAGAGGGAATTTCCGGTGGAGCGGTGAAATGCATT
GAGATCGGAAAGAACACCAACGGCGAAAGCACTCTGCTGGGCCGACACTGACAC
TGAGAGACGAAAGCTAGGGGAGCAAATGGGATTAGAGACCCCAGTAGTCCTAGC
CGTAAACGATGGATACTAGGTGCTGTGCGACTCGACCCGTGCAGTGCTGTAGCTA
ACGCGTTAAGTATCCCGCCTGGGGAGTACGTTCGCAAGAATGAAACTCAAAGGA
ATTGACGGGGGCCCGCACAAGCGGTGGAGCATGTGGTTTAATTCGATGCAAAGC
GAAGAACCTTACCAGGGCTTGACATGCCGCGAATCCTCTTGAAAGAGAGGGGTG
CCCTCGGGAACGCGGACACAGGTGGTGCATGGCTGTCGTCAGCTCGTGCCGTAAG
GTGTTGGGTTAAGTCTCGCAACGAGCGCAACCCTCGTGTTTAGTTGCCACTATGA
GTTTGGAACCCTGAACAGACCGCCGGTGTTAAGCCGGAGGAAGGAGAGGATGAG
GCCAAGTCATCATGCCCCTTATGCCCTGGGCGACACACGTGCTACAATGGGCGGG
ACAAAGGGTCGCGATCTCGCGAGGGTGAGCTAACTCCAAAAACCCGTCCTCAGTT
CGGATTGCAGGCTGCAACTCGCCTGCATGAAGCAGGAATCGCTAGTAATCGCCGG
TCAGCCATACGGCGGTGAATCCGTTCCCGGGCCTTGTACACACCGCCCGTCACAC

TATAGGAGCTGGCCATGTTTGAAGTCATTACCCTTAACCGTAAGGAGGGGGATGC
CTAAGGCTAGGCTTGCGACTGGAGTGAAGTCGTAACAAGGTAGCCGTACTGGAA
GGTGCGGCTGGATCACCTCCTTT

>4513:emu_db:49320 ["4513:ncbi:49320 ['KC912688.91675.93166_U Horedeum vulgare 16S ribosomal RNA, chloroplast']"]

ATCTCATGGAGAGTTTGATCCTGGCTCAGGATGAACGCTGGCGGCATGCTTAACA
CATGCAAGTCGAACGGGAAGTGGTGTTTCCAGTGGCGAACGGGTGAGTAACGCG
TAAGAACCTGCCCTTGGGAGGGGAACAACAACTGGAAACGGTTGCTAATACCCC
GTAGGCTGAGGAGCAAAAGGAGAAATCCGCCCAAGGAGGGGCTCGCGTCTGATT
AGCTAGTTGGTGAGGCAATAGCTTACCAAGGCGATGATCAGTAGCTGGTCCGAG
AGGATGATCAGCCACACTGGGACTGAGACACGGCCCAGACTCCTACGGGAGGCA
GCAGTGGGGAATTTTCCGCAATGGGCGAAAGCCTGACGGAGCAATGCCGCGTGG
AGGTGGAAGGCCTACGGGTCGTCAACTTCTTTTCTCGGAGAAGAAACAATGACGG
TATCTGAGGAATAAGCATCGGCTAACTCTGTGCCAGCAGCCGCGGTAAGACAGA
GGATGCAAGCGTTATCCGGAATGATTGGGCGTAAAGCGTCTGTAGGTGGCTTTTC
AAGTCCGCCGTCAAATCCCAGGGCTCAACCCTGGACAGGCGGTGGAAACTACCA
AGCTGGAGTACGGTAGGGGCAGAGGGAATTTCCGGTGGAGCGGTGAAATGCATT
GAGATCGGAAAGAACACCAACGGCGAAAGCACTCTGCTGGGCCGACACTGACAC
TGAGAGACGAAAGCTAGGGGAGCAAATGGGATTAGAGACCCCAGTAGTCCTAGC
CGTAAACGATGGATACTAGGTGCTGTGCGACTCGACCCGTGCAGTGCTGTAGCTA
ACGCGTTAAGTATCCCGCCTGGGGAGTACGTTCGCAAGAATGAAACTCAAAGGA
ATTGACGGGGGCCCGCACAAGCGGTGGAGCATGTGGTTTAATTCGATGCAAAGC
GAAGAACCTTACCAGGGCTTGACATGCCGCGAATCCTCTTGAAAGAGAGGGGTG
CCCTCGGGAACGCGGACACAGGTGGTGCATGGCTGTCGTCAGCTCGTGCCGTAAG
GTGTTGGGTTAAGTCTCGCAACGAGCGCAACCCTCGTGTTTAGTTGCCACTATGA
GTTTGGAACCCTGAACAGACCGCCGGTGTTAAGCCGGAGGAAGGAGAGGATGAG
GCCAAGTCATCATGCCCCTTATGCCCTGGGCGACACACGTGCTACAATGGGCGGG
ACAAAGGGTCGCGATCTCGCGAGGGTGAGCTAACTCCAAAAACCCGTCCTCAGTT
CGGATTGCAGGCTGCAACTCGCCTGCATGAAGCAGGAATCGCTAGTAATCGCCGG
TCAGCCATACGGCGGTGAATCCGTTCCCGGGCCTTGTACACACCGCCCGTCACAC
TATAGGAGCTGGCCATGTTTGAAGTCATTACCCTTAACCGTAAGGAGGGGGATGC
CTAAGGCTAGGCTTGCGACTGGAGTGAAGTCGTAACAAGGTAGCCGTACTGGAA
GGTGCGGCTGGATCACCTCCTTT

>4513:emu_db:49321 ["4513:ncbi:49321 ['KC912687.92137.93628_U Horedeum vulgare 16S ribosomal RNA, chloroplast']"]

ATCTCATGGAGAGTTTGATCCTGGCTCAGGATGAACGCTGGCGGCATGCTTAACA
CATGCAAGTCGAACGGGAAGTGGTGTTTCCAGTGGCGAACGGGTGAGTAACGCG
TAAGAACCTGCCCTTGGGAGGGGAACAACAACTGGAAACGGTTGCTAATACCCC
GTAGGCTGAGGAGCAAAAGGAGAAATCCGCCCAAGGAGGGGCTCGCGTCTGATT
AGCTAGTTGGTGAGGCAATAGCTTACCAAGGCGATGATCAGTAGCTGGTCCGAG
AGGATGATCAGCCACACTGGGACTGAGACACGGCCCAGACTCCTACGGGAGGCA
GCAGTGGGGAATTTTCCGCAATGGGCGAAAGCCTGACGGAGCAATGCCGCGTGG
AGGTGGAAGGCCTACGGGTCGTCAACTTCTTTTCTCGGAGAAGAAACAATGACGG
TATCTGAGGAATAAGCATCGGCTAACTCTGTGCCAGCAGCCGCGGTAAGACAGA
GGATGCAAGCGTTATCCGGAATGATTGGGCGTAAAGCGTCTGTAGGTGGCTTTTC
AAGTCCGCCGTCAAATCCCAGGGCTCAACCCTGGACAGGCGGTGGAAACTACCA
AGCTGGAGTACGGTAGGGGCAGAGGGAATTTCCGGTGGAGCGGTGAAATGCATT

GAGATCGGAAAGAACACCAACGGCGAAAGCACTCTGCTGGGCCGACACTGACAC
TGAGAGACGAAAGCTAGGGGAGCAAATGGGATTAGAGACCCCAGTAGTCCTAGC
CGTAAACGATGGATACTAGGTGCTGTGCGACTCGACCCGTGCAGTGCTGTAGCTA
ACGCGTTAAGTATCCCGCCTGGGGAGTACGTTCGCAAGAATGAAACTCAAAGGA
ATTGACGGGGGCCCGCACAAGCGGTGGAGCATGTGGTTTAATTCGATGCAAAGC
GAAGAACCTTACCAGGGCTTGACATGCCGCGAATCCTCTTGAAAGAGAGGGGTG
CCCTCGGGAACGCGGACACAGGTGGTGCATGGCTGTCGTCAGCTCGTGCCGTAAG
GTGTTGGGTTAAGTCTCGCAACGAGCGCAACCCTCGTGTTTAGTTGCCACTATGA
GTTTGGAACCCTGAACAGACCGCCGGTGTTAAGCCGGAGGAAGGAGAGGATGAG
GCCAAGTCATCATGCCCCTTATGCCCTGGGCGACACACGTGCTACAATGGGCGGG
ACAAAGGGTCGCGATCTCGCGAGGGTGAGCTAACTCCAAAAACCCGTCCTCAGTT
CGGATTGCAGGCTGCAACTCGCCTGCATGAAGCAGGAATCGCTAGTAATCGCCGG
TCAGCCATACGGCGGTGAATCCGTTCCCGGGCCTTGTACACACCGCCCGTCACAC
TATAGGAGCTGGCCATGTTTGAAGTCATTACCCTTAACCGTAAGGAGGGGGATGC
CTAAGGCTAGGCTTGCGACTGGAGTGAAGTCGTAACAAGGTAGCCGTACTGGAA
GGTGCGGCTGGATCACCTCCTTT

>4513:emu_db:49322 ["4513:ncbi:49322 ['EF115541.123958.125449_U Horedeum vulgare 16S ribosomal RNA, chloroplast']"]

ATCTCATGGAGAGTTTGATCCTGGCTCAGGATGAACGCTGGCGGCATGCTTAACA
CATGCAAGTCGAACGGGAAGTGGTGTTTCCAGTGGCGAACGGGTGAGTAACGCG
TAAGAACCTGCCCTTGGGAGGGGAACAACAACTGGAAACGGTTGCTAATACCCC
GTAGGCTGAGGAGCAAAAGGAGAAATCCGCCCAAGGAGGGGCTCGCGTCTGATT
AGCTAGTTGGTGAGGCAATAGCTTACCAAGGCGATGATCAGTAGCTGGTCCGAG
AGGATGATCAGCCACACTGGGACTGAGACACGGCCCAGACTCCTACGGGAGGCA
GCAGTGGGGAATTTTCCGCAATGGGCGAAAGCCTGACGGAGCAATGCCGCGTGG
AGGTGGAAGGCCTACGGGTCGTCAACTTCTTTTCTCGGAGAAGAAACAATGACGG
TATCTGAGGAATAAGCATCGGCTAACTCTGTGCCAGCAGCCGCGGTAAGACAGA
GGATGCAAGCGTTATCCGGAATGATTGGGCGTAAAGCGTCTGTAGGTGGCTTTTC
AAGTCCGCCGTCAAATCCCAGGGCTCAACCCTGGACAGGCGGTGGAAACTACCA
AGCTGGAGTACGGTAGGGGCAGAGGGAATTTCCGGTGGAGCGGTGAAATGCATT
GAGATCGGAAAGAACACCAACGGCGAAAGCACTCTGCTGGGCCGACACTGACAC
TGAGAGACGAAAGCTAGGGGAGCAAATGGGATTAGAGACCCCAGTAGTCCTAGC
CGTAAACGATGGATACTAGGTGCTGTGCGACTCGACCCGTGCAGTGCTGTAGCTA
ACGCGTTAAGTATCCCGCCTGGGGAGTACGTTCGCAAGAATGAAACTCAAAGGA
ATTGACGGGGGCCCGCACAAGCGGTGGAGCATGTGGTTTAATTCGATGCAAAGC
GAAGAACCTTACCAGGGCTTGACATGCCGCGAATCCTCTTGAAAGAGAGGGGTG
CCCTCGGGAACGCGGACACAGGTGGTGCATGGCTGTCGTCAGCTCGTGCCGTAAG
GTGTTGGGTTAAGTCTCGCAACGAGCGCAACCCTCGTGTTTAGTTGCCACTATGA
GTTTGGAACCCTGAACAGACCGCCGGTGTTAAGCCGGAGGAAGGAGAGGATGAG
GCCAAGTCATCATGCCCCTTATGCCCTGGGCGACACACGTGCTACAATGGGCGGG
ACAAAGGGTCGCGATCTCGCGAGGGTGAGCTAACTCCAAAAACCCGTCCTCAGTT
CGGATTGCAGGCTGCAACTCGCCTGCATGAAGCAGGAATCGCTAGTAATCGCCGG
TCAGCCATACGGCGGTGAATCCGTTCCCGGGCCTTGTACACACCGCCCGTCACAC
TATAGGAGCTGGCCATGTTTGAAGTCATTACCCTTAACCGTAAGGAGGGGGATGC
CTAAGGCTAGGCTTGCGACTGGAGTGAAGTCGTAACAAGGTAGCCGTACTGGAA
GGTGCGGCTGGATCACCTCCTTT

>4513:emu_db:49323 ["4513:ncbi:49323 ['KC912689.92138.93629_U Horedeum vulgare 16S ribosomal RNA, chloroplast']"]

ATCTCATGGAGAGTTTGATCCTGGCTCAGGATGAACGCTGGCGGCATGCTTAACA
CATGCAAGTCGAACGGGAAGTGGTGTTTCCAGTGGCGAACGGGTGAGTAACGCG
TAAGAACCTGCCCTTGGGAGGGGAACAACAACTGGAAACGGTTGCTAATACCCC
GTAGGCTGAGGAGCAAAAGGAGAAATCCGCCCAAGGAGGGGCTCGCGTCTGATT
AGCTAGTTGGTGAGGCAATAGCTTACCAAGGCGATGATCAGTAGCTGGTCCGAG
AGGATGATCAGCCACACTGGGACTGAGACACGGCCCAGACTCCTACGGGAGGCA
GCAGTGGGGAATTTTCCGCAATGGGCGAAAGCCTGACGGAGCAATGCCGCGTGG
AGGTGGAAGGCCTACGGGTCGTCAACTTCTTTTCTCGGAGAAGAAACAATGACGG
TATCTGAGGAATAAGCATCGGCTAACTCTGTGCCAGCAGCCGCGGTAAGACAGA
GGATGCAAGCGTTATCCGGAATGATTGGGCGTAAAGCGTCTGTAGGTGGCTTTTC
AAGTCCGCCGTCAAATCCCAGGGCTCAACCCTGGACAGGCGGTGGAAACTACCA
AGCTGGAGTACGGTAGGGGCAGAGGGAATTTCCGGTGGAGCGGTGAAATGCATT
GAGATCGGAAAGAACACCAACGGCGAAAGCACTCTGCTGGGCCGACACTGACAC
TGAGAGACGAAAGCTAGGGGAGCAAATGGGATTAGAGACCCCAGTAGTCCTAGC
CGTAAACGATGGATACTAGGTGCTGTGCGACTCGACCCGTGCAGTGCTGTAGCTA
ACGCGTTAAGTATCCCGCCTGGGGAGTACGTTCGCAAGAATGAAACTCAAAGGA
ATTGACGGGGGCCCGCACAAGCGGTGGAGCATGTGGTTTAATTCGATGCAAAGC
GAAGAACCTTACCAGGGCTTGACATGCCGCGAATCCTCTTGAAAGAGAGGGGTG
CCCTCGGGAACGCGGACACAGGTGGTGCATGGCTGTCGTCAGCTCGTGCCGTAAG
GTGTTGGGTTAAGTCTCGCAACGAGCGCAACCCTCGTGTTTAGTTGCCACTATGA
GTTTGGAACCCTGAACAGACCGCCGGTGTTAAGCCGGAGGAAGGAGAGGATGAG
GCCAAGTCATCATGCCCCTTATGCCCTGGGCGACACACGTGCTACAATGGGCGGG
ACAAAGGGTCGCGATCTCGCGAGGGTGAGCTAACTCCAAAAACCCGTCCTCAGTT
CGGATTGCAGGCTGCAACTCGCCTGCATGAAGCAGGAATCGCTAGTAATCGCCGG
TCAGCCATACGGCGGTGAATCCGTTCCCGGGCCTTGTACACACCGCCCGTCACAC
TATAGGAGCTGGCCATGTTTGAAGTCATTACCCTTAACCGTAAGGAGGGGGATGC
CTAAGGCTAGGCTTGCGACTGGAGTGAAGTCGTAACAAGGTAGCCGTACTGGAA
GGTGCGGCTGGATCACCTCCTTT

>4513:emu_db:49324 ["4513:ncbi:49324 ['NC_008590.123958.125449_U Horedeum vulgare 16S ribosomal RNA, chloroplast']"]

ATCTCATGGAGAGTTTGATCCTGGCTCAGGATGAACGCTGGCGGCATGCTTAACA
CATGCAAGTCGAACGGGAAGTGGTGTTTCCAGTGGCGAACGGGTGAGTAACGCG
TAAGAACCTGCCCTTGGGAGGGGAACAACAACTGGAAACGGTTGCTAATACCCC
GTAGGCTGAGGAGCAAAAGGAGAAATCCGCCCAAGGAGGGGCTCGCGTCTGATT
AGCTAGTTGGTGAGGCAATAGCTTACCAAGGCGATGATCAGTAGCTGGTCCGAG
AGGATGATCAGCCACACTGGGACTGAGACACGGCCCAGACTCCTACGGGAGGCA
GCAGTGGGGAATTTTCCGCAATGGGCGAAAGCCTGACGGAGCAATGCCGCGTGG
AGGTGGAAGGCCTACGGGTCGTCAACTTCTTTTCTCGGAGAAGAAACAATGACGG
TATCTGAGGAATAAGCATCGGCTAACTCTGTGCCAGCAGCCGCGGTAAGACAGA
GGATGCAAGCGTTATCCGGAATGATTGGGCGTAAAGCGTCTGTAGGTGGCTTTTC
AAGTCCGCCGTCAAATCCCAGGGCTCAACCCTGGACAGGCGGTGGAAACTACCA
AGCTGGAGTACGGTAGGGGCAGAGGGAATTTCCGGTGGAGCGGTGAAATGCATT
GAGATCGGAAAGAACACCAACGGCGAAAGCACTCTGCTGGGCCGACACTGACAC
TGAGAGACGAAAGCTAGGGGAGCAAATGGGATTAGAGACCCCAGTAGTCCTAGC
CGTAAACGATGGATACTAGGTGCTGTGCGACTCGACCCGTGCAGTGCTGTAGCTA
ACGCGTTAAGTATCCCGCCTGGGGAGTACGTTCGCAAGAATGAAACTCAAAGGA
ATTGACGGGGGCCCGCACAAGCGGTGGAGCATGTGGTTTAATTCGATGCAAAGC
GAAGAACCTTACCAGGGCTTGACATGCCGCGAATCCTCTTGAAAGAGAGGGGTG
CCCTCGGGAACGCGGACACAGGTGGTGCATGGCTGTCGTCAGCTCGTGCCGTAAG

GTGTTGGGTTAAGTCTCGCAACGAGCGCAACCCTCGTGTTTAGTTGCCACTATGA
GTTTGGAACCCTGAACAGACCGCCGGTGTTAAGCCGGAGGAAGGAGAGGATGAG
GCCAAGTCATCATGCCCCTTATGCCCTGGGCGACACACGTGCTACAATGGGCGGG
ACAAAGGGTCGCGATCTCGCGAGGGTGAGCTAACTCCAAAAACCCGTCCTCAGTT
CGGATTGCAGGCTGCAACTCGCCTGCATGAAGCAGGAATCGCTAGTAATCGCCGG
TCAGCCATACGGCGGTGAATCCGTTCCCGGGCCTTGTACACACCGCCCGTCACAC
TATAGGAGCTGGCCATGTTTGAAGTCATTACCCTTAACCGTAAGGAGGGGGATGC
CTAAGGCTAGGCTTGCGACTGGAGTGAAGTCGTAACAAGGTAGCCGTACTGGAA
GGTGCGGCTGGATCACCTCCTTT

>4513:emu_db:49325 ["4513:ncbi:49325 ['NC_008590.92685.94176_U Horedeum vulgare 16S ribosomal RNA, chloroplast']"]

ATCTCATGGAGAGTTTGATCCTGGCTCAGGATGAACGCTGGCGGCATGCTTAACA
CATGCAAGTCGAACGGGAAGTGGTGTTTCCAGTGGCGAACGGGTGAGTAACGCG
TAAGAACCTGCCCTTGGGAGGGGAACAACAACTGGAAACGGTTGCTAATACCCC
GTAGGCTGAGGAGCAAAAGGAGAAATCCGCCCAAGGAGGGGCTCGCGTCTGATT
AGCTAGTTGGTGAGGCAATAGCTTACCAAGGCGATGATCAGTAGCTGGTCCGAG
AGGATGATCAGCCACACTGGGACTGAGACACGGCCCAGACTCCTACGGGAGGCA
GCAGTGGGGAATTTTCCGCAATGGGCGAAAGCCTGACGGAGCAATGCCGCGTGG
AGGTGGAAGGCCTACGGGTCGTCAACTTCTTTTCTCGGAGAAGAAACAATGACGG
TATCTGAGGAATAAGCATCGGCTAACTCTGTGCCAGCAGCCGCGGTAAGACAGA
GGATGCAAGCGTTATCCGGAATGATTGGGCGTAAAGCGTCTGTAGGTGGCTTTTC
AAGTCCGCCGTCAAATCCCAGGGCTCAACCCTGGACAGGCGGTGGAAACTACCA
AGCTGGAGTACGGTAGGGGCAGAGGGAATTTCCGGTGGAGCGGTGAAATGCATT
GAGATCGGAAAGAACACCAACGGCGAAAGCACTCTGCTGGGCCGACACTGACAC
TGAGAGACGAAAGCTAGGGGAGCAAATGGGATTAGAGACCCCAGTAGTCCTAGC
CGTAAACGATGGATACTAGGTGCTGTGCGACTCGACCCGTGCAGTGCTGTAGCTA
ACGCGTTAAGTATCCCGCCTGGGGAGTACGTTCGCAAGAATGAAACTCAAAGGA
ATTGACGGGGGCCCGCACAAGCGGTGGAGCATGTGGTTTAATTCGATGCAAAGC
GAAGAACCTTACCAGGGCTTGACATGCCGCGAATCCTCTTGAAAGAGAGGGGTG
CCCTCGGGAACGCGGACACAGGTGGTGCATGGCTGTCGTCAGCTCGTGCCGTAAG
GTGTTGGGTTAAGTCTCGCAACGAGCGCAACCCTCGTGTTTAGTTGCCACTATGA
GTTTGGAACCCTGAACAGACCGCCGGTGTTAAGCCGGAGGAAGGAGAGGATGAG
GCCAAGTCATCATGCCCCTTATGCCCTGGGCGACACACGTGCTACAATGGGCGGG
ACAAAGGGTCGCGATCTCGCGAGGGTGAGCTAACTCCAAAAACCCGTCCTCAGTT
CGGATTGCAGGCTGCAACTCGCCTGCATGAAGCAGGAATCGCTAGTAATCGCCGG
TCAGCCATACGGCGGTGAATCCGTTCCCGGGCCTTGTACACACCGCCCGTCACAC
TATAGGAGCTGGCCATGTTTGAAGTCATTACCCTTAACCGTAAGGAGGGGGATGC
CTAAGGCTAGGCTTGCGACTGGAGTGAAGTCGTAACAAGGTAGCCGTACTGGAA
GGTGCGGCTGGATCACCTCCTTT

>4513:emu_db:49326 ["4513:ncbi:49326 ['AC252172.60777.62190 Horedeum vulgare 16S ribosomal RNA, chloroplast']"]

AGTTCGGTCCATATCCGTTGTGGGAGTTAGAGCATTGAGAGGACCTTTCCCTAGT
ACGAGAAGACCGGGAAGGACGCACCTCTGGTGTACCAGTTATCGTGCCTACAGT
AAACACTGGGTATCCAAGTGCGGAGAGGATAACTGTTGAAAGCATATAAGTAGT
AAGACCACCCCAAGATGAGTGCTCTCTCCTCCGACTTCCCTAGAGCCTCCGGTAT
CACAGCCGAGACAGCGACGGGTTCTCCACCCATACGTTTTGGCTCAAATATGAAT
TTTCCGCAATGGACGAAAGCCTGACGAAGCAATGCCGCGTGGAGGTGGAAGGCC

TACGGGTCACCAACTTCTTTTCTCTAAGAAGAAACAATGACGGTATCTGAGGAAT
AAGCATTGGCTAACTCTATGCCAGCAGCCGCGGTAAGACAGAGGATGCAAGCGT
TATCCGAAATGATTGGGCGTAGAGCGTCTGTAGGTGGCTTTTCAAGTCCGCCGTC
AAATCCCAGGGCTCAACCCTGGATAGGCGGTGGAAACTACCAAGCTGGAGTACG
GTAGGGGCAGACGGAATTTCCGGTGGAGCGGTGAAATGCATTGAGATCGGAAAG
AACTCCAACGGCGAAAGCACTCTGCTGGGCCGACACTAACACTGAGAAACGAAA
GCTAGGGGAGCAAATGGGATTAGAGACCCCAGTAGTCCTACCTGTAAATGATGG
ATACTAGGTGATGTGCGACTCGACCCGTGCAGTGTTGTAGCTAACGCGTTAAGTA
TCCTGCCTGGGAGTACGTTTGCAAGAATGAAACTCAAAGGAATTGATGGGGGCCC
GCACAAGCGGTGGAGCATGTGGTTTAATTCGATGCAAAGAGAAGAACCTTACCA
GGGCTTGACATGTCGCGAATCCTCTTGAAAGAGAGGGGTGCCCTCGGGAACGCG
GACACAGGTGGTGCATGGTTGTCGTCAGCTCGTGTCGTAAGGTGTTGTGTTAAGT
CTCGCAACAAGCGCAACCCTCGTGTTTAGTTGCCACTAGGAGTTTGGAACCCTGA
ACAGACCGCCGGTGTTAAGCCGGAGGAAGGAGAGGATGAGGCCAAGTCATCATG
CCCCTTATGCCCTAGGCGACACACGTGCTACAATGGGCGGGGCAAAGGGTCGCG
GTCTCGCGAGGGTGAGCTAACTCCAAAAACCCATCCTCAGTTCGGATTGCAGGCT
ACAACTCGCCTGCATGAAGCAGGAATCGCTAGTAATCGCCGGTCAGCCATACGGT
GGTGAATCTGTTCCCGGGCCTTGTACACACCGCCCGTCACACTATAGTAGGTGGC
CATGTTTGAAGTCATTACCCTTAACCGTAAGGAAGGGGATGCCTAAGGCTAGGCT
TGCGACTGGAGTGAAGTCATAACAAGGTAGCCGTACTGGAAGGTGCGGCTG

>4513:emu_db:49327 ["4513:ncbi:49327 ['AC249525.19787.21256 Horedeum vulgare 16S ribosomal RNA, chloroplast']"]

AGAGTTTGATCCTGGCTCAGGATGAACGCTGGCGGCATGCTTAACACATGCAAGT
CGAACGGGAAGTGGTGTTTCCAGTGGCGAACGGGTGAGTAACGCGTAAGAACCT
GCCCTTGGGAGGGGAACAACAACTGGAAACGGTTGCTAATACCCCGTCGGCTGA
GGAGCAAAAGGAGAAATCCGCCCAAGGAGGGGCTCGCGTCTGATTAGCTAGTTG
GTGAGGAAATAGCTTACCAAGGCGATGATCAGTAGCTGGTCCGAGAGGATGATC
AGCCACACTAGGACTGAGACACGGCCCAGACTCCTACGGGAGGCAGCAGTGGGG
AATTTTCCGCAATGGGCGAAAGCCTGACGGAGCAATGCCGCGTGGAGGTGGAAG
GCCTACGGGTCGTCAACTTCTTTTCTCGGAGAAGAAACAATGACGGTATCTGAGG
AATAAGCATCGGCTAACTCTGTGCCAGCAGCCGCGGTAAGACAGAGGATGCAAG
CGTTATCCGGAATGATTGGGCGTAAAGCGTCTGTAGGTGGCTTTTCAAGTCCGCC
GTCAAATCCCAGGGCTCAACCCTGGACAGGCGGTGGAAACTACCAAGCTGGAGT
ACGGTAGGGGCAGAGGGAATTTCCGGTGGAGCGGTGAAATGCATTGAGATCGGA
AAGAACACCAACGGCGAAAGCACTCTGCTGGGCCGACACTGACACTCAGAGACG
AAAGCTAGGGGAGCAAATGGGATTAGAGACCCTAGTAGTCCTAGCCGTAAACGA
TGGATACTAGGTGCTGTGCGACTCGACCCGTGCAGTGTTGTAGCTAACGCGTTAA
GTATCCCGCCTGGGGAGTACGTTCGCAAGAATGAAACTCAAAGGAATTGACGGG
GGCCTGCACAAGCGGTGGAGCATGTGGTTTAATTCGATGCAAAGCGAAGAACCTT
ACCAGGGCTTGACATGCCGCGAATCCTCTTGAAAGAGAGGGGTGCCCTCGGGAA
CGCGGACACAGGTGGTGCATGGCTGTCGTCAGCTCGTGCCGTAAGGTGTTGGGTT
AAGTCTCGCAACGAGCGCAACCCTCGTGTTTAGTTGCCACTATGAGTTTGGAACC
CTGAACAGACCGCCGGTGTCAAGCCGGAGGAAGGAGAGGATGAGGCCAAGTCAT
CATGCCCCTTATGCCCTGGGCGACACACGTGCTACAATGGGCGGACAAAGGGTC
GCAATCTCGCGAGGGTGAGCTAACTCCAAAAACCCGTCCTCAGTTCGGATTGTAG
GCTGCAACTCGCCTGCATGAAGCAGGAATCGCTAGTAATCGCCGGTCAGCCATAC
GGCGGTGAATCCGTTCCCGGGCCTTGTACACACCGCCCGTCACACTATAGGAGCT
GACCATGTTTGAAGTCATTACCCTTAACCGTAAGGAGGGGGATGCCTAAGGCTAG
GCTTGCGACTGGAGTGAAGTCGTAACAAGGTAGCCGTACTGGAAGGTGCGGCTG
